# Supplementary material for: The Italian Osteopathic Practitioners Estimates and RAtes (OPERA) study: How osteopaths work
Source: PLoS One. 2020 Jul 2;15(7):e0235539. doi: 10.1371/journal.pone.0235539 (PMC7332305; doi:10.1371/journal.pone.0235539)
Supplement: S1 Data — (DOCX) [file pone.0235539.s001.docx]

| **Treatment technique** | **Always** | **Often** | **Regularly** | **Seldom** | **Never** | ***p*** |
| --- | --- | --- | --- | --- | --- | --- |
| Automatic shifting and fluid body approach  Alone  Associated | 426 (15.14)  274 (13.67) | 538 (19.12)  429 (21.43) | 559 (19.87)  392 (19.58) | 351 (12.47)  302 (15.09) | 940 (33.40)  605 (30.23) | 0.006 |
| Fascial techniques  Alone  Associated | 730 (25.94)  626 (31.27) | 1113 (39.55)  813 (40.61) | 646 (22.96)  361 (18.03) | 189 (6.72)  150 (7.49) | 136 (4.83)  52 (2.60) | < 0.001 |
| Fluid techniques  Alone  Associated | 189 (6.72)  166 (8.29) | 669 (23.77)  588 (29.37) | 930 (33.05)  647 (32.32) | 621 (22.07)  383 (19.13) | 405 (14.39)  218 (10.89) | < 0.001 |
| Functional techniques  Alone  Associated | 643 (22.85)  555 (27.72) | 1012 (35.96)  768 (38.36) | 703 (24.98)  475 (23.73) | 379 (13.47)  155 (7.74) | 77 (2.74)  49 (2.45) | < 0.001 |
| GOT  Alone  Associated | 710 (25.23)  571 (28.52) | 825 (29.32)  673 (33.62) | 601 (21.36)  323 (16.13) | 361 (12.82)  223 (11.14) | 317 (11.27)  212 (10.59) | < 0.001 |
| HVLA  Alone  Associated | 602 (21.39)  521 (26.02) | 953 (33.87)  646 (32.27) | 659 (23.42)  474 (23.68) | 416 (14.78)  254 (12.69) | 184 (6.54)  107 (5.34) | 0.001 |
| MET  Alone  Associated | 516 (18.34)  431 (21.53) | 908 (32.27)  709 (35.41) | 784 (27.86)  463 (23.13) | 462 (16.41)  296 (14.79) | 144 (5.12)  103 (5.14) | < 0.001 |
| Cranial techniques  Alone  Associated | 645 (22.92)  523 (26.12) | 961 (34.15)  777 (38.81) | 652 (23.17)  450 (22.48) | 320 (11.37)  123 (6.15) | 236 (8.39)  129 (6.44) | < 0.001 |
| Neurovisceral and neurolymphatic relfex  Alone  Associated | 67 (2.38)  43 (2.15) | 216 (7.68)  162 (8.09) | 474 (16.84)  452 (22.58) | 769 (27.33)  472 (23.57) | 1288 (45.77)  873 (43.61) | < 0.001 |
| Percussion and vibration  Alone  Associated | 88 (3.13)  81 (4.05) | 319 (11.34)  218 (10.89) | 660 (23.45)  468 (23.38) | 735 (26.12)  558 (27.86) | 1012 (35.96)  677 (33.82) | 0.207 |
| Trigger points  Alone  Associated | 170 (6.04)  184 (9.19) | 560 (19.89)  393 (19.63) | 844 (29.99)  552 (27.57) | 684 (24.32)  543 (27.13) | 556 (19.76)  330 (16.48) | < 0.001 |
| PINS  Alone  Associated | 86 (3.06)  108 (5.39) | 263 (9.35)  215 (10.74) | 511 (18.16)  312 (15.58) | 522 (18.54)  355 (17.74) | 1432 (50.89)  1012 (50.55) | < 0.001 |
| Soft tissue  Alone  Associated | 689 (24.48)  534 (26.67) | 1030 (36.60)  767 (38.31) | 647 (22.99)  462 (23.08) | 290 (10.32)  182 (9.09) | 158 (5.61)  57 (2.85) | < 0.001 |
| Visceral manipulation  Alone  Associated | 797 (28.32)  717 (35.81) | 1205 (42.82)  856 (42.76) | 606 (21.54)  348 (17.38) | 136 (4.83)  39 (1.95) | 70 (2.49)  42 (2.10) | < 0.001 |
| Toggle  Alone  Associated | 32 (1.14)  49 (2.45) | 183 (6.50)  115 (5.74) | 302 (10.73)  152 (7.59) | 373 (13.26)  269 (13.44) | 1924 (68.37)  1417 (70.78) | < 0.001 |
| **Other Advices** | **Always** | **Often** | **Regularly** | **Seldom** | **Never** | ***p*** |
| Active motion exercises  Alone  Associated | 1267 (45.02)  1006 (50.25) | 1155 (41.05)  762 (38.06) | 354 (12.58)  224 (11.19) | 22 (0.78)  10 (0.50) | 16 (0.57)  0 (0.00) | < 0.001 |
| ADL advices  Alone  Associated | 1223 (43.46)  1010 (50.45) | 1157 (41.12)  690 (34.47) | 360 (12.79)  247 (12.34) | 69 (2.45)  43 (2.14) | 5 (0.18)  12 (0.60) | < 0.001 |
| Dietary advices  Alone  Associated | 726 (25.80)  543 (27.12) | 877 (31.17)  638 (31.87) | 809 (28.75)  512 (25.57) | 327 (11.61)  250 (12.49) | 75 (2.67)  59 (2.95) | 0.176 |
| Ergonomic advices  Alone  Associated | 817 (29.03)  712 (35.56) | 1034 (36.74)  685 (34.22) | 651 (23.14)  381 (19.04) | 203 (7.22)  118 (5.89) | 109 (3.87)  106 (5.29) | < 0.001 |
| Pain management  Alone  Associated | 790 (28.07)  699 (34.91) | 936 (33.26)  683 (34.12) | 678 (24.09)  379 (18.93) | 289 (10.28)  176 (8.79) | 121 (4.30)  65 (3.25) | < 0.001 |

Supplementary material 1 -Treatment Techniques between *alone* and *associated* osteopaths and other advices

| **Variable** | **Alone (%)** | | | | **Associated (%)** | | | |  |
| --- | --- | --- | --- | --- | --- | --- | --- | --- | --- |
| New diagnostic examination  Yes  No  Not always | 1837 (65.28)  65 (2.31)  912 (32.41) | | | | 1377 (68.78)  30 (1.50)  595 (29.72) | | | |  |
| **Diagnostic Technique** | | **Always** | **Often** | **Regularly** | | **Seldom** | **Never** | ***p*** | |
| Assessment of visceral mobility  Alone  Associated | | 1378 (49.0)  1068 (53.3) | 763 (27.1)  547 (27.3) | 412 (14.6)  258 (12.9) | | 193 (6.8)  77 (3.9) | 68 (2.5)  52 (2.6) | < 0.001 | |
| Assessment of the cranium  Alone  Associated | | 1740 (61.8)  1210 (60.4) | 618 (22.0)  504 (25.2) | 342 (12.2)  222 (11.1) | | 86 (3.1)  43 (2.1) | 28 (0.9)  23 (1.2) | 0.032 | |
| Fascial testing  Alone  Associated | | 1306 (46.4)  1011 (50.5) | 801 (28.4)  581 (29.0) | 477 (17.0)  313 (15.6) | | 177 (6.3)  76 (3.8) | 53 (1.9)  21 (1.1) | <0.001 | |
| Inspection  Alone  Associated | | 888 (31.4)  709 (35.4) | 633 (22.4)  333 (16.6) | 482 (17.0)  400 (20.00) | | 230 (8.6)  159 (8.00) | 581 (20.6)  401 (20.0) | <0.001 | |
| Muscle function test  Alone  Associated | | 54 (1.9)  53 (2.7) | 191 (6.8)  143 (7.1) | 489 (17.4)  396 (19.8) | | 757 (26.9)  509 (25.4) | 1323 (47.0)  901 (45.0) | 0.069 | |
| Neurolymphatic reflex tests  Alone  Associated | | 1933 (68.7)  1371 (68.5) | 565 (20.1)  397 (19.8) | 190 (6.7)  197 (9.8) | | 92 (3.3)  31 (1.6) | 34 (1.2)  6 (0.3) | <0.001 | |
| Palpation of structures’ position  Alone  Associated | | 1459 (51.7)  1072 (53.6) | 593 (21.0)  447 (22.4) | 328 (11.1)  263 (13.1) | | 163 (5.7)  77 (3.8) | 271 (9.5)  143 (7.1) | <0.001 | |
| Palpation of movement  Alone  Associated | | 173 (6.1)  95 (4.8) | 273 (9.7)  211 (10.5) | 779 (27.7)  601 (30.0) | | 979 (34.8)  717 (35.8) | 610 (21.7)  378 (18.9) | 0.017 | |
| Percussion and auscultation  Alone  Associated | | 365 (13.0)  278 (13.9) | 570 (20.3)  405 (20.2) | 773 (27.5)  539 (26.9) | | 637 (22.6)  542 (27.1) | 469 (16.6)  238 (11.9) | <0.001 | |
| Tender points and trigger points  Alone  Associated | | 691 (24.6)  554 (27.7) | 933 (33.2)  710 (35.5) | 818 (29.1)  532 (26.6) | | 294 (10.4)  177 (8.8) | 78 (2.7)  29 (1.4) | <0.001 | |
| Classic orthopedic tests  Alone  Associated | | 559 (19.9)  445 (22.2) | 853 (30.3)  648 (32.4) | 878 (31.2)  587 (29.3) | | 391 (13.9)  243 (12.1) | 133 (4.7)  79 (4.0) | 0.034 | |
| Classic neurologic tests  Alone  Associated | | 484 (17.1)  410(20.5) | 899 (32.0)  604 (30.2) | 857 (30.5)  651 (32.5) | | 448 (15.9)  260 (13.0) | 126 (4.5)  77 (3.8) | 0.002 | |
| Range of motion  Alone  Associated | | 1121 (39.8)  878 (43. 9) | 936 (33.3)  624 (31.2) | 475 (16.9)  324 (16.2) | | 199 (7.1)  134 (6.6) | 83 (2.9)  42 (2.1) | 0.040 | |
| Otoscopy  Alone  Associated | | 6 (0.2)  0 (0.0) | 41 (1.5)  17 (0.9) | 139 (4.9)  127 (6.3) | | 318 (11.3)  221 (11.0) | 2310 (82.1)  1637 (81.8) | 0.016 | |
| Urine test  Alone  Associated | | 0 (0.0)  0 (0.0) | 22 (0.8)  5 (0.3) | 22 (0.8)  11 (0.5) | | 153 (5.4)  74 (3.7) | 2617 (93.0)  1912 (95.5) | 0.002 | |

Supplementary material 2. Diagnostic examination between *alone* and *associated* osteopaths and diagnostic techniques
