# Supplementary material for: The Italian Osteopathic Practitioners Estimates and RAtes (OPERA) study: How osteopaths work
Source: PLoS One. 2020 Jul 2;15(7):e0235539. doi: 10.1371/journal.pone.0235539 (PMC7332305; doi:10.1371/journal.pone.0235539)
Supplement: Attachment — Submitted filename: PONE-D-19-35428_reviewer.pdf [file pone.0235539.s002.pdf]

PLOS ONE

The Italian Osteopathic Practitioners Estimates and RAtes (OPERA) study: how osteopaths work.

--Manuscript Draft--

**Manuscript Number:**	PONE-D-19-35428
**Article Type:**	Research Article
**Full Title:**	The Italian Osteopathic Practitioners Estimates and RAtes (OPERA) study: how osteopaths work.
**Short Title:**	The Italian Osteopathic Practitioners Estimates and RAtes (OPERA) study: how osteopaths work.
**Corresponding Author:**	Giacomo Consorti<br>Centre pour l'Etude la Recherche et la Diffusion Osteopathique<br>Rome, ITALY
**Keywords:**	osteopath; scope of practice; cross-sectional; osteopathic practice; OPERA; OPERA-IT
**Abstract:**	Introduction: The scope of practice of the osteopathic profession in Italy is underreported. The first part of the present study investigated the Italian osteopaths' profile, focusing on the socio-demographic information and geographical distribution together with the main characteristics of their education. The OPERA-IT study highlighted that the majority of respondents declared to work alone (58.4%), while the remaining declared to work in association with other professionals. Since teamwork and networking are recognized as fundamental aspects of healthcare , the present study aims to compare the osteopathic practise, diagnostic, and treatment modalities of osteopaths who work alone and osteopaths who work associated to other healthcare professionals to highlight possible differences. Moreover, patients' characteristics will be presented.<br>Methods: The OPERA-IT study population was chosen to provide a representative sample. A web campaign was set up to inform the Italian osteopathic professionals before the beginning of the study. The OPERA IT study used a validated questionnaire. The questionnaire was translated into Italian following the WHO recommendation. The questionnaire was composed of 57 items grouped in five sections, namely: socio-demographics, osteopathic education, and training, working profile, organisation, and management of the clinical practice and patient profile. The survey was delivered online through a dedicated platform.<br>Results: 4,816 individuals completed the survey. Osteopaths who work alone represented the majority of the sample (n=2814; 58.4%). Osteopaths who work with other professionals declared to collaborate mostly with physiotherapists (n=1121; 23.3%), physicians with speciality (n=1040; 21.6%), and other osteopaths (n=943; 19.6%). The two groups showed heterogeneous characteristics. Significative differences were observed in all the factors, namely: geographical distribution, age, gender, training, working contract and working place, patient per day and time for each patient, fees, and the average waiting period to book an appointment. The principal component analysis supported a ten-component model and explained 80.5% of the total variance. The analysis showed that osteopaths working alone have an increased probability (OR = 0.91; CI 95%: 0.88 - 0.94; p<0.01) of using systemic diagnostic and treatment techniques and have distinct clinical features with higher probability (OR =0.92; 0.88 - 0.96; p<0.01) of spending less time with patients, being paid less but treating a higher number of patients per week. The most represented patients' age groups were 41-64 years old (n=4452; 92.4%) and 21-40 years old (n=4291; 89.1%). Similarly, the most reported new patients age groups were 41-64 years old (n=4221; 87.7%) and 21-40 years old (n=3364; 69.9%). The most common presenting complaints were back pain, cervical pain, cervicobrachialgia, sciatica, shoulder pain, and headaches.<br>Conclusions: Osteopathic practice in Italy seems to be characterised by interprofessional collaboration, mostly with physiotherapists. Our results highlighted two different profiles in terms of sociodemographic characteristics and work modalities between osteopaths who work alone and those who work associated with other professionals. Although according to the respondents, people of all ages consult Italian

Powered by Editorial Manager® and ProduXion Manager® from Aries Systems Corporation

	osteopaths, the majority of patients are adults. Most of them have been referred to osteopathy by other patients or acquaintances. Patients seek osteopathic care mostly for musculoskeletal related complaints.
**Order of Authors:**	Francesco Cerritelli
	Giacomo Consorti
	Patrick LS van Dun
	Jorge E Esteves
	Paola Sciomachen
	Massimo Valente
	Eleonora Lacorte
	Nicola Vanacore
**Additional Information:**	
**Question**	**Response**
**Financial Disclosure**<br><br>Enter a financial disclosure statement that describes the sources of funding for the work included in this submission. Review the submission guidelines for detailed requirements. View published research articles from *PLOS ONE* for specific examples.<br><br>This statement is required for submission and **will appear in the published article** if the submission is accepted. Please make sure it is accurate.<br><br>**Unfunded studies**<br>Enter: *The author(s) received no specific funding for this work.*<br><br>**Funded studies**<br>Enter a statement with the following details:<br>• Initials of the authors who received each award<br>• Grant numbers awarded to each author<br>• The full name of each funder<br>• URL of each funder website<br>• Did the sponsors or funders play any role in the study design, data collection and analysis, decision to publish, or preparation of the manuscript?<br>• **NO** - Include this sentence at the end of your statement: *The funders had no role in study design, data collection and analysis, decision to publish, or preparation of the manuscript.*<br>• **YES** - Specify the role(s) played.	FC received 1 grant from Registro degli Osteopati d'Italia https://www.registro-osteopati-italia.com/<br><br>The funders had no role in study design, data collection and analysis, decision to publish, or preparation of the manuscript.

* typeset	
**Competing Interests**<br><br>Use the instructions below to enter a competing interest statement for this submission. On behalf of all authors, disclose any competing interests that could be perceived to bias this work—acknowledging all financial support and any other relevant financial or non-financial competing interests.<br><br>This statement **will appear in the published article** if the submission is accepted. Please make sure it is accurate. View published research articles from *PLOS ONE* for specific examples.<br><br>**NO authors have competing interests**<br><br>Enter: *The authors have declared that no competing interests exist.*<br><br>**Authors with competing interests**<br><br>Enter competing interest details beginning with this statement:<br><br>*I have read the journal's policy and the authors of this manuscript have the following competing interests: [insert competing interests here]*<br><br>* typeset	The authors have declared that no competing interests exist.
**Ethics Statement**<br><br>Enter an ethics statement for this submission. This statement is required if the study involved:<br><br>• Human participants<br>• Human specimens or tissue<br>• Vertebrate animals or cephalopods<br>• Vertebrate embryos or tissues<br>• Field research<br><br>Write "N/A" if the submission does not require an ethics statement.	Participants were requested to read and understand all the information about the study and to give their informed consent by starting the survey as clearly stated in the survey presentation page. The study received the approval of the Institutional Review Board of the Foundation COME Collaboration (12/2016).

General guidance is provided below. Consult the submission guidelines for detailed instructions. **Make sure that all information entered here is included in the Methods section of the manuscript.**<br><br>**Format for specific study types**<br><br>**Human Subject Research (involving human participants and/or tissue)**<br>• Give the name of the institutional review board or ethics committee that approved the study<br>• Include the approval number and/or a statement indicating approval of this research<br>• Indicate the form of consent obtained (written/oral) or the reason that consent was not obtained (e.g. the data were analyzed anonymously)<br><br>**Animal Research (involving vertebrate animals, embryos or tissues)**<br>• Provide the name of the Institutional Animal Care and Use Committee (IACUC) or other relevant ethics board that reviewed the study protocol, and indicate whether they approved this research or granted a formal waiver of ethical approval<br>• Include an approval number if one was obtained<br>• If the study involved *non-human primates*, add *additional details* about animal welfare and steps taken to ameliorate suffering<br>• If anesthesia, euthanasia, or any kind of animal sacrifice is part of the study, include briefly which substances and/or methods were applied<br><br>**Field Research**<br><br>Include the following details if this study involves the collection of plant, animal, or other materials from a natural setting:<br>• Field permit number<br>• Name of the institution or relevant body that granted permission	
**Data Availability**<br><br>Authors are required to make all data	No - some restrictions will apply

underlying the findings described fully available, without restriction, and from the time of publication. PLOS allows rare exceptions to address legal and ethical concerns. See the PLOS Data Policy and FAQ for detailed information.<br><br>A Data Availability Statement describing where the data can be found is required at submission. Your answers to this question constitute the Data Availability Statement and **will be published in the article**, if accepted.<br><br>**Important:** Stating 'data available on request from the author' is not sufficient. If your data are only available upon request, select 'No' for the first question and explain your exceptional situation in the text box.<br><br>Do the authors confirm that all data underlying the findings described in their manuscript are fully available without restriction?	
**Describe where the data may be found in full sentences. If you are copying our sample text, replace any instances of XXX with the appropriate details.**<br><br>• If the data are **held or will be held in a public repository**, include URLs, accession numbers or DOIs. If this information will only be available after acceptance, indicate this by ticking the box below. For example: *All XXX files are available from the XXX database (accession number(s) XXX, XXX.)*.<br>• If the data are all contained **within the manuscript and/or Supporting Information files**, enter the following: *All relevant data are within the manuscript and its Supporting Information files.*<br>• If neither of these applies but you are able to provide **details of access elsewhere**, with or without limitations, please do so. For example:<br><br>*Data cannot be shared publicly because of [XXX]. Data are available from the*	Data represent third party data that cannot be made publicly available due to ethical and legal restrictions. Ethically, public availability would compromise participants confidentiality as the minimal dataset would include information on participants age, gender, workplace. Due to our permission as given by the Registro Osteopati d'Italia we are restricted not to<br>disclose data to a third party. However, data can be requested by contacting irb@comecollaboration.org.

XXX Institutional Data Access / Ethics Committee (contact via XXX) for researchers who meet the criteria for access to confidential data.<br><br>*The data underlying the results presented in the study are available from (include the name of the third party and contact information or URL).*<br>• This text is appropriate if the data are owned by a third party and authors do not have permission to share the data.<br><br>* typeset	
Additional data availability information:	

Powered by Editorial Manager® and ProduXion Manager® from Aries Systems Corporation

Manuscript Click here to download Manuscript Main Manuscript.docx

The Italian Osteopathic Practitioners Estimates and RAtes (OPERA) study: how osteopaths work.

Francesco Cerritelli[1], Giacomo Consorti[1,2,6], Patrick L. S. van Dun[3], Jorge E. Esteves[4,5], Paola Sciomachen[6], Massimo Valente[1], Eleonora Lacorte[7], Nicola Vanacore[7], OPERA-IT Group

Affiliations

1. Clinical-based Human Research Department, Research Division, COME Collaboration, Pescara, Italy
2. Research Department of the Centre pour l'Etude, la Recherche et la Diffusion Osteopathiques (C.E.R.D.O.), Rome, Italy
3. Belgium National Centre, Foundation COME Collaboration, Mechelen, Belgium
4. Gulf National Centre, Foundation COME Collaboration, Riyadh, KSA,
5. University College of Osteopathy, London, United Kingdom
6. Registro degli Osteopati d'Italia, Milan, Italy
7. National Institute of Health, Rome, Italy

Address for corrispondence

Giacomo Consorti

Largo Ceresio, 4

22100 Como (CO)

Italy

ph: +39 3405204077

email: giacomo.consorti@gmail.com

Abstract

Introduction: The scope of practice of the osteopathic profession in Italy is underreported. The first part of the present study investigated the Italian osteopaths' profile, focusing on the socio-demographic information and geographical distribution together with the main characteristics of their education. The OPERA-IT study highlighted that the majority of respondents declared to work alone (58.4%), while the remaining declared to work in association with other professionals. Since teamwork and networking are recognized as fundamental aspects of healthcare, the present study aims to compare the osteopathic practise, diagnostic, and treatment modalities of osteopaths who work alone and osteopaths who work associated to other healthcare professionals to highlight possible differences. Moreover, patients' characteristics will be presented.

Methods: The OPERA-IT study population was chosen to provide a representative sample. A web campaign was set up to inform the Italian osteopathic professionals before the beginning of the study. The OPERA IT study used a validated questionnaire. The questionnaire was translated into Italian following the WHO recommendation. The questionnaire was composed of 57 items grouped in five sections, namely: socio-demographics, osteopathic education, and training, working profile, organisation, and management of the clinical practice and patient profile. The survey was delivered online through a dedicated platform.

Results: 4,816 individuals completed the survey. Osteopaths who work alone represented the majority of the sample (n=2814; 58.4%). Osteopaths who work with other professionals declared to collaborate mostly with physiotherapists (n=1121; 23.3%), physicians with speciality (n=1040; 21.6%), and other osteopaths (n=943; 19.6%). The two groups showed heterogeneous characteristics. Significative differences were observed in all the factors, namely: geographical distribution, age, gender, training, working contract and working place,

patient per day and time for each patient, fees, and the average waiting period to book an appointment. The principal component analysis supported a ten-component model and explained 80.5% of the total variance. The analysis showed that osteopaths working alone have an increased probability (OR = 0.91; CI 95%: 0.88 - 0.94; p<0.01) of using systemic diagnostic and treatment techniques and have distinct clinical features with higher probability (OR =0.92; 0.88 - 0.96; p<0.01) of spending less time with patients, being paid less but treating a higher number of patients per week. The most represented patients' age groups were 41-64 years old (n=4452; 92.4%) and 21-40 years old (n=4291; 89.1%). Similarly, the most reported new patients age groups were 41-64 years old (n=4221; 87.7%) and 21-40 years old (n=3364; 69.9%). The most common presenting complaints were back pain, cervical pain, cervicobrachialgia, sciatica, shoulder pain, and headaches.

Conclusions: Osteopathic practice in Italy seems to be characterised by interprofessional collaboration, mostly with physiotherapists. Our results highlighted two different profiles in terms of sociodemographic characteristics and work modalities between osteopaths who work alone and those who work associated with other professionals. Although according to the respondents, people of all ages consult Italian osteopaths, the majority of patients are adults. Most of them have been referred to osteopathy by other patients or acquaintances. Patients seek osteopathic care mostly for musculoskeletal related complaints.

Introduction

Osteopathy is a growing health profession in Italy. In a recent national opinion survey conducted on a sample of 800 participants by Eumetra Monterosa (1), it has been reported that over 10 million Italians received osteopathic care, particularly for musculoskeletal related problems (70% of the reported reasons of the consultation). 90% of the sample in the

study ~~declared to be~~ satisfied with the osteopathic care provided (1). The first part of the present study investigated the Italian osteopaths' profile, focusing on the socio-demographic information and geographical distribution together with the main characteristics of their education (2). The scope of practice of the osteopathic profession in Italy is, however, significantly underreported. Therefore, other health care professionals and the general public may not be aware of the nature of the osteopathic practice, including commonly treated clinical conditions, therapeutic interventions, and patients' characteristics. This is particularly important because the osteopathic care provided may vary amongst individual clinicians and between countries (3–9). For example, American osteopathic physicians have a scope of practice equivalent to medical practitioners (10). In Europe, Denmark, Finland, France, Iceland, Italy, Liechtenstein, Malta, Portugal, Switzerland, Turkey, and the UK have regulated osteopathy (11). In contrast to their US counterparts - i.e., 'osteopathic physicians', European osteopaths have limited practice rights, and they are called 'osteopaths' (10). In Italy, with the approval of the law 3/2018, osteopathy has been recognized as a healthcare profession (12). However, the regulation process is still ongoing, and despite the recent publication of the Core Competence of the Italian Osteopaths (13), the proper scope of practice of Italian osteopaths has not yet been published.

Van Dun et al. (6) were the first authors to profile ~~the~~ osteopathic practitioners in countries without statutory regulation in osteopathy using the Benelux Osteosurvey tool. The Osteopathic Practitioners Estimates and RAtes (OPERA) project was developed starting from the Osteosurvey tool. OPERA is a European-based census aimed to profile the osteopathic profession across Europe (2). Arguably, it is a relevant tool for all the stakeholders interested in obtaining up-to-date and reliable information regarding the geo-distribution, prevalence, incidence, and profile of osteopaths and their patients in Europe. The OPERA study has been initially conducted in Italy (2) and is currently being carried out in Spain, Andorra, Belgium,

Luxembourg, and Portugal. Several studies investigated the primary reasons for consultation and the characteristics of patients receiving osteopathic care (5,8,14–20). However, none of these studies was carried out on the Italian population.

The aim of the OPERA Italy (OPERA-IT) study was to profile osteopathic practice in Italy by surveying osteopaths across the country regarding socio-demographic information (2), their practice and patients' characteristics, presenting symptoms and clinical problems, use of diagnostic and treatment modalities. The OPERA-IT study highlighted that the majority of respondents ~~declared to work~~ alone (58.4%), while the remaining declared to work in association with other professionals. Since teamwork and networking are recognized as fundamental aspects of healthcare (21), the present study aims to compare the osteopathic practise, diagnostic, and treatment modalities of osteopaths who work alone and osteopaths who work associated to other healthcare professionals to highlight possible differences. Moreover, patients' characteristics will be presented.

Methods

The SUrvey Reporting GuidelinE (SURGE) (22) was used as a reporting guideline for this article.

Population

The OPERA-IT study population was chosen to provide a representative sample. For that purpose, the recruitment strategy followed specific criteria and was as inclusive as possible without compromising the representativeness of the sample. Hence the recruitment was aimed to obtain the highest possible participation among those who fulfilled the following inclusion criteria: older than 18 years old, the successful completion of any training leading to a Diploma in Osteopathy (DO) or equivalent (23), and the participants had to be practising

as an osteopath. Participation or successful completion of any training courses on single techniques and osteopathic approaches, which did not lead to a DO or equivalent title (23), was not considered sufficient to be included in the study. Therefore individuals matching this profile were excluded. Exclusion criteria were set to prevent non-osteopaths who attended short and non-degree/professional awarding courses in, e.g., craniosacral technique or spinal manipulation to participate and to lower the representativeness of the sample. OPERA-IT used an online survey; therefore professionals with no access to the online platform were excluded. Individuals who could not understand and respond in Italian and individuals with physical or mental impairments that precluded participation in the online survey were also excluded. Participants were requested to read and understand all the information about the study and to give their informed consent by starting the survey as clearly stated in the survey presentation page. The study received the approval of the Institutional Review Board of the Foundation COME Collaboration (12/2016).

Recruitment

A website for promoting OPERA IT was created. A web campaign was set up to inform the Italian osteopathic professionals before the beginning of the study. The campaign was structured as a combined social media and newsletter strategy. The largest osteopathic national voluntary registering body (ROI) took part in the promotion by sending a newsletter to all its current members. At the time in which the study was carried out, ROI included approximately 2,500 members. Since it was estimated that the ROI members alone were not representative of the Italian osteopaths population, an additional e-campaign was established to reach the osteopathic education institutions, the other voluntary registering bodies and professional associations and the known osteopathic internet providers/specialised websites

(i.e., tuttosteopatia.it) asking them to advertise the study to all of their members through the official OPERA IT e-flyer. In addition to the e-flyer, all the participating osteopathic education institutions were provided with a physical flyer and other advertising material to be displayed at their location. Furthermore, a manual based search on white-pages was conducted to identify other sources of information. The promotion strategy was carried in twelve steps. Each step consisted of the dispatch of the e-flyer to all the different mailing lists. The time interval for the promotion strategy, recruitment, and data collection were five-months. All participants, upon the completion of the survey, received an invitation containing the credential to attend free continuous professional development (CPD) webinars on a dedicated online platform. Participants were able to log in at any time during the study period and follow the pre-recorded webinars

Survey tool

The OPERA IT study used a validated questionnaire (6). The questionnaire was translated into Italian following the WHO recommendation. Therefore, a forward-backwards translation was performed by two bilingual English-Italian translators with experience in the field of demographic health research. The questionnaire is composed of 57 items grouped in five sections, namely: socio-demographics, osteopathic education, and training, working profile, organisation, and management of the clinical practice and patient profile. A pilot survey was delivered to twenty Italian-speaking osteopaths. The pilot aimed to gather information about the degree of comprehensibility of the items. For that purpose face-to-face interviews were conducted by the research team and the survey was modified in accordance with the suggestions of the participants. The first OPERA IT publication reported the results of the

first three sections of the survey (2). The present study will report the results from the remaining two sections.

The OPERA survey online platform, the symmetric keys data encryption, and the certified data centre were the same used for the first part of the present study (2). Therefore, all of the gathered information was processed and hosted following data protection regulations, the answers were anonymised, and the IP addresses were not accessible to the research team. The system automatically managed the link between the StudyID and the email address of respondents so that double response was not allowed. Only OPERA research personnel had access to the complete, anonymised dataset.

Privacy

The anonymity and privacy of data were respected following the European directive 2002/58/CE of the European Parliament. Gathered data will be stored for 5 years to allow benchmarking and further analyses.

~~**Information guidelines**~~

~~In this study will be reported participants answers regarding their practice and patients' characteristics, presenting symptoms and clinical problems, use of diagnostic and treatment modalities~~

Statistical analysis

Data were analysed using mean, median, mode, point estimates, range, standard deviation, and 95% confidence interval. For dichotomous measures, relative risk was used. Statistical analyses were based on a univariate and multivariate approach. R statistical programme (v.

3.1.3) was used to perform statistical analysis. A value of alpha less than 0.05 was considered as significant.

Principal-Component Analysis (PCA) and logistic analysis

The examination of the data indicated that items had non-normal distributions, which is common for categorical data. Categorical PCA, a form of PCA specifically geared to discrete ordinal values, was run using R Statistical program (v3.5). The fundamental idea of PCA is to examine the matrix of item correlations to reduce the information into a smaller set of components. These components can form the basis for hypotheses about latent factors. In the presence of high intercorrelation, items are assumed to be measuring the same latent component. All items are assumed to load onto all components.

Component eigenvalues represent the relative share of total variance accounted for by that component and can, therefore, be used to select the number of components. We selected components being greater than 1, in order to determine the dimensions underlying the pattern of interrelationships among the scores considered. Thus, reducing the number of the original variables and increasing the interpretability of the summary components. To aid interpretability, the component matrix was rotated using Promax oblique rotation, which assumes that components are correlated. Rotations are a change in the coordinate of the component solution that makes the pattern of loadings more pronounced and, therefore clearer. Components loadings, which are the correlation coefficients between the items and the identified components, are reported. The square of component loadings represents the amount of variance in the item explained by the component.

The resulting components of PCA were used as independent variables in a logistic regression model with the dependent variable "alone" yes/no. The interpretation of the meaning of each factor was defined in a collaborative way among the authors. In general, all items were

categorised into (1) musculoskeletal; (2) systemic; (3) clinical. Each category was characterized by a number of affine elements (clusters). The systemic category included both diagnostic items, as the visceral, cranium, and fascial diagnostic techniques, and treatment items, as neurovisceral and neurolymphatic reflex techniques and fascial techniques. The musculoskeletal category included as well both diagnostic and treatment items, as palpation of the position of the anatomical structures, and trigger points treatment. The "clinical" category was characterized by items which describe the clinical practice of the osteopathic professional, such as the duration and the fees of the first and follow-up clinical encounters, the average waiting period to schedule an appointment or the number of patient per week encountered by the practitioner.

Results

4,816 individuals completed the survey. 196 questionnaires, corresponding to a 4% attrition rate, were left uncompleted. Composition and geographical distribution of the whole sample are reported by Cerritelli et al. (2). Osteopaths who work alone represented the majority of the sample (n=2814; 58.4%). Osteopaths who work with other professionals ~~declared to collaborate~~ mostly with physiotherapists (n=1121; 23.3%), physicians with speciality (n=1040; 21.6%), and other osteopaths (n=943; 19.6%). A ~~comprehensive~~ description of osteopaths' collaborations is available in Table 1.

	N	%
Alone	2814	58.4
Associated	2002	41.6
osteopath	943	19.6
GP	390	8.1
physiotherapist	1121	23.3
occupational therapist	74	1.5
psychologist	746	15.5
speech therapist	317	6.6
dietician	671	13.9
dentistry	433	9.0
massage therapist	446	9.3
physician with specialty	1040	21.6
optometrist	162	3.4

other	493	10.2

Table 1. Working collaborations of osteopaths

Comparison between osteopaths who work alone and associated

The two groups showed heterogeneous characteristics. ~~Significative~~ differences were observed in all the factors, namely: geographical distribution, age, gender, training, working contract and working place, patient per day and time for each patient, fees, and the average waiting period to book an appointment. In particular, referring to the geographical distribution, osteopaths who work in the macro-region "centre" have the highest odd to work associated with other professionals (OR = 1.37). Younger osteopaths (20-29 years old) have the highest odd to work associated compared to other age groups (OR of other age groups compared to the 20-29 age group < 1). Female osteopaths have 59% more likely to work associated compared to male ones (OR = 1.59). Osteopaths who graduated through a full-time curriculum (T1) have a higher chance of working associated compared to those from a part-time one (T2) (OR for T2 compared to T1 = 0.71). Osteopaths who work as self-employed in their clinic have the highest probability of working in association with other professionals (OR. 1.23). Osteopaths who work in a university have a 77% increased probability of working associated than osteopaths who work in other places (OR = 1.77). Osteopaths who have 11 to 15 clinical encounters per day are more likely to work in association than others (OR = 1.50) as well those whose clinical encounter lasts 46-60 minutes (OR = 2.01). Osteopaths who charge between 51 and 60 euros per both first consultation and follow-ups have more than the double probability to work in association than others (OR = 2.37; OR = 2.94). Osteopaths who have a waiting period for the booking in between 2 and 3 weeks have almost a probability almost three times higher to work in association compared to the others (OR = 2.93). Extensive data about the comparison between the characteristics of the two groups are available in table 2.

Variable		Alone (%)	Associated (%)	*p*	OR (Alo/Ass)*
Geographical distribution					
	North-west	883 (31.4)	610 (30.5)		
	North-east	714 (25.4)	442 (22.1)	<0.001	0.90 (0.77 – 1.05)
	Centre	618 (21.9)	586 (29.2)		1.37 (1.18 – 1.60)
	South	503 (17.9)	310 (15.5)		0.89 (0.75 – 1.06)
	Islands	96 (3.4)	54 (2.7)		0.81 (0.54 – 1.15)
Age					
	20-29	527 (18.7)	518 (25.9)		
	30-39	1083 (38.5)	845 (42.2)		0.79 (0.68 – 0.92)
	40-49	699 (24.8)	420 (21.0)	<0.001	0.61 (0.52 – 0.73)
	50-59	395 (14.0)	201 (10.0)		0.52 (0.42 – 0.64)
	60-65	94 (3.4)	18 (0.9)		0.19 (0.12 – 0.33)
	>65	16 (0.6)	0 (0.0)		NA
Gender					
	Male	1999 (71.0)	1215 (60.7)	<0.001	
	Female	815 (29.0)	787 (39.3)		1.59 (1.41 – 1.79)
Training					
	T1	851 (30.2)	758 (37.9)	<0.001	
	T2	1963 (69.8)	1244 (62.1)		0.71 (0.63 – 0.80)
Work					
	DO employed	31 (1.1)	34 (1.7)	<0.001	
	DO self-employed in own clinic	2511 (89.2)	1600 (79.9)		0.58 (0.36 – 0.95)
	DO self-employed not in own clinic	272 (9.7)	368 (18.4)		1.23 (0.74 – 2.06)
Working Place					
	Private practice	2510 (92.1)	1547 (77.3)	<0.001	
	Clinic/hospital	482 (17.1)	510 (25.5)	<0.001	1.72 (1.49 – 1.97)
	Osteopathy School	557 (19.8)	495 (24.7)	<0.001	1.44 (1.26 – 1.65)
	University	79 (2.8)	86 (4.3)	0.005	1.77 (1.29 – 2.41)
	Other	374 (13.3)	356 (17.8)	<0.001	1.54 (1.32 – 1.81)
Patients/day					
	0-5	1396 (49.6)	867 (43.3)		
	<0.0016-10	1142 (40.6)	909 (45.4)	<0.001	1.28 (1.13 – 1.45)
	11-15	225 (8.0)	210 (10.5)		1.50 (1.22 – 1.85)
	16-20	39 (1.4)	10 (0.5)		0.41 (0.21 – 0.83)
	>20	12 (0.4)	6 (0.3)		0.81 (0.30 – 2.15)
Time/patient					
	<30 minutes	57 (2.0)	23 (1.2)		
	30-45 minutes	484 (17.2)	331 (16.5)	<0.001	1.69 (1.02 – 2.81)
	46-60 minutes	1651 (58.8)	1338 (66.8)		2.01 (1.23 – 3.28)
	>60 minutes	622 (22.1)	310 (15.5)		1.24 (0.75 – 2.04)
Fee first consultation					
	<25 euros	27 (1.0)	11 (0.6)		
	26-30 euros	73 (2.6)	23 (1.2)		0.77 (0.33 – 1.80)
	31-40 euros	198 (7.0)	103 (5.2)		1.28 (0.61 – 2.68)
	41-50 euros	907 (32.2)	574 (28.6)		1.55 (0.76 – 3.16)
	51-60 euros	671 (23.8)	648 (32.4)	<0.001	2.37 (1.17 – 4.82)
	61-70 euros	405 (14.4)	352 (17.5)		2.13 (1.04 – 4.36)
	71-80 euros	285 (10.1)	163 (8.1)		1.40 (0.68 – 2.90)
	81-90 euros	113 (4.1)	61 (3.1)		1.33 (0.62 – 2.85)
	91-100 euros	77 (2.7)	39 (1.9)		1.24 (0.56 – 2.77)
	>100 euros	58 (2.1)	28 (1.4)		1.18 (0.51 – 2.73)
Fee following consultations					
	<25 euros	43 (1.5)	12 (0.60)		
	26-30 euros	100 (3.5)	50 (2.50)		1.79 (0.87 – 3.70)
	31-40 euros	340 (12.1)	229 (11.4)		2.41 (1.25 – 4.68)
	41-50 euros	944 (33.6)	673 (33.6)		2.55 (1.34 – 4.88)
	51-60 euros	676 (24.0)	555 (27.8)	<0.001	2.94 (1.54 – 5.63)
	61-70 euros	370 (13.2)	292 (14.6)		2.83 (1.46 – 5.46)
	71-80 euros	184 (6.6)	125 (6.3)		2.43 (1.23 – 4.80)
	81-90 euros	59 (2.0)	38 (1.9)		2.31 (1.08 – 4.93)
	91-100 euros	75 (2.7)	28 (1.4)		1.34 (0.62 – 2.90)
	>100 euros	23 (0.8)	0 (0.00)		NA
Average waiting period					
	Same day	69 (2.5)	20 (1.00)		
	Within 1 week	1559 (55.4)	1136 (56.7)		2.51 (1.52 – 4.16)
	1 week < X ≤ 2 weeks	827 (29.4)	612 (30.6)	<0.001	2.55 (1.54 – 4.25)
	2 weeks < X ≤ 3 weeks	126 (4.5)	107 (5.3)		2.93 (1.67 – 5.13)
	3 weeks < X ≤ 4 weeks	97 (3.4)	62 (3.1)		2.21 (1.22 – 3.98)
	> 4 weeks	136 (4.8)	65 (3.3)		1.65 (0.92 – 2.94)

Table 2. characteristics of the two groups (*alone* vs *associated*).
*OR is computed for the probability of working alone using the first value of each variable as the exposure factor.

PCA and logistic analysis

The principal component analysis supported a ten-component model (Table 3), based on eigenvalues included between 6.8 (PC-1) to 1.1 (PC-10). This model explained 80.5% of the total variance and appeared interpretable and therefore was retained. Components emerging from the analysis included all items referred to the 3 categories. Few items have found to have loading values below -0.40, whereas a distinct number of items had values above 0.30 or below -0.30. Collectively items that correlated the most were those related to the category clinical, i.e. time to patient and fees.

Following the PCA, the ten-components model was loaded into a logistic regression in order to identify those components that associated significantly with the Alone/Associated dependent variable.

As shown in Table 4, the logistic analysis demonstrated that only seven factors were significantly related to being "alone". This result means that those components that resulted significantly associated include items characterising the difference between being "alone or associated" in practice. Among those, there is clear evidence that osteopaths working alone have an increased probability (OR = 0.91; CI 95%: 0.88 - 0.94; p<0.01) of using systemic diagnostic and treatment techniques (see PC-3 items in Table 3) and have distinct clinical features with higher probability (OR =0.92; 0.88 - 0.96; p<0.01) of spending less time with patients, being paid less but treating a higher number of patients per week (see PC-6 items in Table 3).

	**PC1**	**PC2**	**PC3**	**PC4**	**PC5**	**PC6**	**PC7**	**PC8**	**PC9**	**PC10**
**Region**	0.00	-0.03	**0.28**	**-0.20**	-0.08	**-0.41**	0.16	**-0.35**	0.01	**0.30**
**Gender**	0.00	-0.07	**0.28**	-0.01	0.06	-0.13	**0.24**	-0.09	0.14	**-0.29**
**Age**	0.07	**0.30**	-0.19	-0.13	-0.12	-0.07	0.01	**0.30**	0.07	-0.04
**Training_type**	-0.03	**-0.23**	0.13	0.07	0.12	-0.12	**0.22**	**-0.56**	-0.08	-0.03
**Time for new patient**	0.01	-0.11	**0.24**	0.11	**0.20**	**-0.44**	0.05	**0.22**	0.02	**0.24**
**Time for returning patient**	0.03	-0.08	**0.26**	0.08	**0.20**	**-0.40**	0.02	**0.25**	0.07	**0.29**
**Fee first consultation**	-0.02	**0.30**	-0.14	**-0.25**	0.12	**-0.35**	**0.20**	-0.06	-0.11	**-0.22**
**Fee following consultation**	0.00	**0.29**	-0.12	**-0.31**	0.16	**-0.34**	0.16	-0.03	-0.10	**-0.24**
**Average waiting period**	0.01	**0.24**	-0.10	-0.16	0.12	0.07	**0.24**	-0.05	0.17	**0.46**
**N patients per working week**	-0.02	**0.25**	-0.18	**-0.20**	0.06	**0.23**	**0.21**	-0.11	0.07	**0.32**

Diagnostic techniques - assessment of visceral mobility	-0.16	0.11	**0.27**	**-0.23**	-0.05	-0.04	**-0.28**	-0.14	0.11	0.04
Diagnostic techniques - assessment of the cranium (neuro- and viscerocranium)	-0.04	**0.21**	**0.35**	-0.04	0.10	0.03	-0.17	-0.01	-0.02	-0.05
Diagnostic techniques - fascial testing	-0.11	0.17	**0.28**	**-0.20**	-0.09	0.15	-0.02	0.10	0.13	-0.04
Diagnostic techniques - inspection	-0.12	0.10	-0.05	0.02	0.04	-0.02	**-0.38**	-0.06	**-0.23**	**0.21**
Diagnostic techniques - muscle function testing	-0.16	0.18	-0.07	**0.29**	0.07	-0.01	-0.08	-0.13	-0.10	0.03
Diagnostic techniques - neurolymphatic reflex tests	**-0.20**	-0.08	-0.04	**-0.24**	0.04	0.02	-0.11	0.03	**-0.24**	-0.08
Diagnostic techniques - palpation of position/structures	-0.05	0.14	0.09	**0.20**	**0.23**	0.13	0.11	**0.20**	**-0.38**	-0.04
Diagnostic techniques - palpation of movement	-0.19	0.13	-0.06	0.17	0.01	-0.12	**-0.23**	0.04	0.16	0.03
Diagnostic techniques - percussion and auscultation	**-0.24**	-0.13	-0.11	0.05	-0.10	-0.04	0.17	0.13	**0.26**	-0.09
Diagnostic techniques - tender points and trigger points	**-0.24**	-0.12	-0.11	-0.07	**0.39**	0.11	-0.07	0.04	0.17	0.00
Diagnostic techniques - classic orthopedic tests	**-0.24**	-0.06	-0.12	-0.05	**0.39**	0.04	-0.09	0.02	0.18	0.00
Diagnostic techniques - classic neurologic tests	**-0.26**	-0.12	-0.12	0.02	**0.23**	0.10	0.00	0.11	0.10	-0.06
Diagnostic techniques - Range Of Motion (ROM)	**-0.20**	-0.14	-0.04	-0.06	**0.30**	0.13	0.00	0.06	-0.09	-0.01
Diagnostic techniques - Otoscopy	-0.09	0.18	-0.13	**0.23**	0.00	-0.12	-0.13	**-0.20**	0.13	-0.16
Diagnostic techniques - urine test	-0.05	0.13	-0.13	0.12	0.04	-0.13	**-0.22**	-0.16	**0.38**	-0.19
Treatment techniques - automatic shifting and fluid body approach	0.03	**0.28**	0.18	**0.22**	**0.22**	0.16	0.16	-0.02	-0.04	0.02
Treatment techniques - fascial techniques	-0.17	0.07	**0.27**	-0.04	-0.08	**0.25**	0.17	-0.01	0.12	-0.08
Treatment techniques - fluid techniques	-0.17	0.13	0.11	0.15	-0.03	0.17	**0.21**	0.15	0.06	-0.04
Treatment techniques - functional techniques	-0.15	0.09	0.18	0.04	0.08	0.06	0.14	-0.08	-0.08	-0.16
Treatment techniques - GOT/TBA	**-0.23**	-0.07	-0.04	-0.02	-0.12	0.01	0.09	0.03	**-0.27**	-0.08
Treatment techniques - HVLA	**-0.23**	-0.10	-0.13	-0.17	-0.07	-0.06	-0.03	-0.09	**-0.27**	0.09
Treatment techniques - MET	**-0.22**	-0.12	-0.04	-0.05	-0.10	-0.10	-0.02	**0.22**	-0.15	-0.10
Treatment techniques - neurocranial and viscerocranial techniques	-0.16	0.12	**0.22**	-0.02	-0.07	0.00	-0.08	-0.01	-0.11	-0.03
Treatment techniques - neurovisceral and neurolymphatic reflex techniques	-0.17	**0.20**	-0.04	**0.33**	-0.13	-0.04	0.02	-0.03	-0.10	-0.06
Treatment techniques - percussion and vibration techniques	-0.18	0.15	0.00	0.12	**-0.22**	-0.09	-0.05	**0.21**	0.01	0.06
Treatment techniques - trigger points	**-0.23**	-0.13	-0.08	0.02	**-0.22**	-0.07	**0.27**	0.09	**0.21**	-0.04
Treatment techniques - Progressive Inhibition of Neuromuscular Structures (PINS)	**-0.20**	0.05	-0.05	0.16	-0.12	-0.14	0.16	0.00	-0.09	0.16
Treatment techniques - soft and connective tissue techniques	**-0.21**	-0.09	0.10	-0.12	-0.18	-0.06	0.12	-0.02	0.01	0.18
Treatment techniques - visceral manipulations	**-0.20**	0.01	**0.22**	**-0.25**	-0.16	-0.06	-0.16	-0.15	0.08	0.10
Treatment techniques - toggle-techniques	-0.16	0.03	-0.08	0.12	-0.10	-0.13	0.13	**-0.33**	-0.04	**0.29**

Factor loadings above 0.20 (positive or negative) are in bold

Table 3. Principal-Component Analysis results

Coefficients	Estimated	Std. Error	z value	Pr(>|z|)	OR	95% CI
**(intercept)**	0.35	0.03	11.84	**<0.01**	1.42	1.34 - 1.51
**PC1**	0.07	0.01	6.39	**<0.01**	1.08	1.05 - 1.10
**PC2**	0.01	0.02	0.98	0.33	1.02	0.99 - 1.05
**PC3**	-0.10	0.02	-5.72	**<0.01**	0.91	0.88 - 0.94
**PC4**	0.03	0.02	1.22	0.22	1.03	0.98 - 1.07
**PC5**	-0.03	0.02	-1.24	0.21	0.97	0.93 - 1.02
**PC6**	-0.09	0.02	-3.51	**<0.01**	0.92	0.88 - 0.96
**PC7**	-0.12	0.03	-4.60	**<0.01**	0.89	0.84 - 0.93
**PC8**	0.13	0.03	4.91	**<0.01**	1.14	1.08 - 1.21
**PC9**	0.07	0.03	2.47	**0.01**	1.07	1.02 - 1.14
**PC10**	0.09	0.03	2.97	**<0.01**	1.09	1.03 - 1.16

Table 4. Logistic Analysis of the principal components

Patients characteristics

The most represented age groups treated within a six months period prior to the census were 41-64 years old (n=4452; 92.4%) and 21-40 years old (n=4291; 89.1%). Similarly, the most reported new patients age groups were 41-64 years old (n=4221; 87.7%) and 21-40 years old (n=3364; 69.9%). Respondents reported that the majority of their patients were self-referred, whether this was based on advice from other patients or acquaintances. The most common body regions requiring osteopathic care were the cervical and lumbar spine. The most common presenting complaints were back pain, cervical pain, cervicobrachialgia, sciatica, shoulder pain, and headaches. The majority of respondents indicated to have no preference of specific patients groups to work with (e.g., paediatrics, athletes, artists) (n=4106; 85.26%).

Discussion

The variables studied are part of the OPERA questionnaire, which evaluates the characteristics of the osteopathic population in a representative national sample. After an initial evaluation of their distribution (2), the scores were modulated with a statistical analysis procedure, in order to further identify the profile of the osteopathic practitioner with peculiar characteristics (components), which allowed better defining the profiles of the studied population.

The OPERA-IT was the first national census relevant to osteopathy in Italy. Data provided by the participants might represent critical new findings relating to osteopathic practice and patients characteristics that have not been observed through other national healthcare data sets (e.g. *Istituto Nazionale di Statistica, Istituto Superiore di Sanità)*. The results of this study provide a comparison between the osteopathic professionals who work alone and those who work in association with other professionals to highlight possible differences in terms of geographical distribution, age, gender, type of training, working place and modalities, patients per day, time of the treatment, fees, and average waiting period for booking an

appointment. Moreover, it describes patients' characteristics in terms of age, referring modalities. Our results highlighted two different profiles between osteopaths who work alone and those who work associated with other professionals. The former have an increased probability of the 8% (PC-1; $p < 0.01$) to not deliver musculoskeletal related diagnostic and treatment techniques, in particular, tender and trigger points assessment, orthopaedic tests, neurologic tests, range of motion tests, General Osteopathic Treatment (GOT), High Velocity and Low Amplitude techniques (HVLA), Muscle Energy Techniques (MET) (table 3). Moreover, osteopaths who work alone are 9% more likely (PC-3; $p < 0.01$) to perform systemic diagnostic and treatment techniques such as the assessment of visceral mobility, cranium assessment, fascial testing, and cranial and visceral manipulations (table 3). Osteopaths who work in association with other professionals have a higher probability (PC-6; 8%; $p < 0.01$) to have a short duration of the treatment and low treatment fees and to have more average patients per week (table 3).

Emerging evidence on the added value of effective interprofessional healthcare teams has created new perspectives on interprofessional collaboration (24–26).

The interprofessional practice has been described as a process that can affect three domains in healthcare; namely, enhancing patient experience with treatment, improving population health and decreasing healthcare costs per capita (27).

Capacity shortages, an ageing population with numerous chronic conditions and new scientific discoveries, require the cooperation of both clinicians and non-clinical members of the healthcare team (28,29).

Contemporary healthcare strategies accept interprofessional practice as an irremissible method to address complex issues. While interprofessional cooperation is beneficial to both practitioners and patients (30), it is still not fully in place (31). Whitehead (32) identified several advantages in applying interprofessional practice for the management of complex

conditions. The author argued that interprofessional practice creates an environment in which the group exceeds the parts' number; common goals are set, and everyone is working towards common goals; the chance to discuss with peers highlights the strengths and weaknesses of the working group through the exchange of experiences and knowledge. This helps break down distrust walls and reduces rivalry. Hierarchies become flatter and more accessible. Moreover, various professional experiences offer the possibility of innovative and creative activities and to identify gaps in practice; partnerships and partnerships result in a more productive way to distribute and use resources effectively; patients can see a more positive, focused and coordinated approach to their health needs and have more faith in it. Finally, there is a higher likelihood of more intensive and holistic approach, which is particularly relevant to the osteopathic practice. Whitehead (32) also highlighted different disadvantages of not engaging in interprofessional practice. The author stated that lone practitioners often act in an individualistic way. This means that weaknesses and mistakes are not solved, and probably they are perpetuated, there is no acknowledgement of good practice, and there are no opportunities to enhance practice. Environments are competitive in a destructive way, the hierarchies are strict, and the position of power is held through manipulative and aggressive behaviour. Perspectives and attitudes are kept isolated and limited. This suppresses the dissemination of information and ideas, fostering a practitioner centred practice. In lone practice, professional groups are protective, guarded, and mistrustful, and this may lead to professional disputes (33). The competitive climate fosters fights for resources. This might lead to a less efficient and less successful practice (32). Moreover, the author argues that in lone practice, there is a greater likelihood of clinical, reductionist, and mechanistic treatment being provided, particularly in terms of health services.

Our findings confirmed a well-established trend among other relevant surveys (5,6,8,15–17,19) showing that primary reasons for osteopathic consultation are musculoskeletal disorders mostly related to the spine.

In general, although the scope of practice of osteopathic profession might be influenced by the regulation status, professional profile, and cultural factors related to the country, our study found several similarities with the other European and international surveys. Our findings supported some of the already known trends about the scope of practice of osteopathy, helping to strengthen what might start to be considered an international shared descriptive framework of the profession.

Results from OPERA-IT might help to define the profile of osteopathic professionals through the perspective of Italian osteopaths. This could be of use in supporting the regulation process providing materials for constructive and informed discussions with policymakers and other stakeholders. Current data might be used to tailor regulatory strategies based on policy outcomes. Moreover, professional associations and registers may benefit from present study data in terms of understanding of the working modalities of their associates and to monitor the national trends of the primary reasons for the osteopathic consultation. Lastly, there are advantages for osteopathic practitioners: to be able to tailor their continuous professional development to the needings of the Italian population and to assess their practice is up to date with the current trend of the profession on the national ground.

Strengths and weaknesses of this study

To the best of our knowledge, this study is the first to highlight the differences between the clinical profile of osteopathic practitioners who work alone and those who work in association with other professionals in Italy. However, this study showed estimates that might not be completely representative of the osteopathic Italian population. Moreover, self-

reporting data might be influenced by response bias. Furthermore, data reported is from a nation-wide survey and thus might not be generalisable to other socio-cultural contexts.

Conclusions

Osteopathic practice in Italy seems to be characterised by interprofessional collaboration, mostly with physiotherapists. Our results highlighted two different profiles in terms of sociodemographic characteristics and work modalities between osteopaths who work alone and those who work associated with other professionals. Although according to the respondents, people of all ages consult Italian osteopaths, the majority of patients are adults. Most of them have been referred to osteopathy by other patients or acquaintances. Patients seek osteopathic care mostly for musculoskeletal related complaints.

The findings of the present study provide valuable insights into the osteopathic profession in Italy, which might be taken into consideration during the regulation process about the professional profile of competencies of the osteopathic profession in Italy. Follow-up studies have been planned to track future changes within the osteopathic profession.

Acknowledgments

The authors sincerely thank Prof. Angelo Manfredi and Prof. Fabrizio Consorti for their help in reviewing the paper. The OPERA-IT group is composed by: Marcello Luca Marasco (ABEos), Alberto Maggiani (AIMO), Antonio Cavallaro (AISERCO), Dario Silvestri (ASOMI), Joseph Zurlo and Marco Petracca (CERDO), Mauro Fornari (CIO), Alessandro Rapisarda (CSDOI), Giacomo Lo Voi (CSOT), Liria Papa (ICOM), Sandro Tamagnini (ICOMM), Roberta Filipazzi (IEMO), Tatiana Stirpe (Meta Osteopatia), Saverio Colonna (OSCE), Alessandro Gavazzi (SOFI), Andrea Manzotti and Andrea Bergna (SOMA), Sbarbaro Marco (SSOI), Federico Franscini (APO), Guglielmo Donnaquio (Osteopatia per

Bambini), Alessandro Parisi (SIOS), Massimo Valente (Tuttosteopatia), Emanuele Botti (Advanced Osteopathy), osteopatiriconosciuti.

Author Contributions

Conceptualization: Francesco Cerritelli, Patrick L. S. van Dun, Jorge E. Esteves, Paola Sciomachen, Eleonora Lacorte, Nicola Vanacore.

Data curation: Francesco Cerritelli, Giacomo Consorti.

Formal analysis: Francesco Cerritelli, Jorge E. Esteves, Giacomo Consorti.

Funding acquisition: Francesco Cerritelli.

Investigation: Francesco Cerritelli, Patrick L. S. van Dun, Jorge E. Esteves.

Methodology: Francesco Cerritelli, Patrick L. S. van Dun, Jorge E. Esteves, Giacomo Consorti, Paola Sciomachen, Eleonora Lacorte, Nicola Vanacore.

Project administration: Francesco Cerritelli, Patrick L. S. van Dun, Jorge E. Esteves, Eleonora Lacorte.

Resources: Francesco Cerritelli, Massimo Valente

Software: Francesco Cerritelli, Massimo Valente

Supervision: Francesco Cerritelli, Patrick L. S. van Dun, Jorge E. Esteves, Paola Sciomachen, Eleonora Lacorte, Nicola Vanacore, Massimo Valente

Validation: Francesco Cerritelli, Patrick L. S. van Dun, Jorge E. Esteves, Nicola Vanacore.

Visualization: Francesco Cerritelli, Jorge E. Esteves, Giacomo Consorti.

Writing – original draft: Francesco Cerritelli, Patrick L. S. van Dun, Jorge E. Esteves, Giacomo Consorti.

Writing – review & editing: Francesco Cerritelli, Patrick L. S. van Dun, Jorge E. Esteves, Giacomo Consorti, Eleonora Lacorte, Nicola Vanacore.

References

1. Eumetra Monterosa. L’opinione degli italiani sull’osteopatia. 2017.
2. Cerritelli F, van Dun PLS, Esteves JE, Consorti G, Sciomachen P, Lacorte E, et al. The Italian Osteopathic Practitioners Estimates and RAtes (OPERA) study: A cross sectional survey. PloS one [Internet]. 2019 [cited 2019 Dec 20];14(1):e0211353. Available from: http://www.ncbi.nlm.nih.gov/pubmed/30682169
3. GOsC. Results – snapshot survey 2001. London; 2001.
4. KPMG. Report A: how do osteopaths practice? Zurich; 2011.
5. Burke SR, Myers R, Zhang AL. A profile of osteopathic practice in Australia 2010-2011: a cross sectional survey. BMC musculoskeletal disorders [Internet]. 2013 Aug 1 [cited 2019 Dec 20];14:227. Available from: http://www.ncbi.nlm.nih.gov/pubmed/23915239
6. Dun P van, Nicolaie M, of AVM-IJ, 2016 undefined. State of affairs of osteopathy in the Benelux: Benelux Osteosurvey 2013. Elsevier [Internet]. [cited 2019 Dec 20]; Available from: https://www.sciencedirect.com/science/article/pii/S1746068916000043
7. CIULLA L, FOUCART J, LEPERS Y, CULLUS P. Enquête d’opinions sur la perception de l’ostéopathie au sein de la population belge francophone. mainslibres.ch [Internet]. [cited 2019 Dec 20]; Available from: http://www.mainslibres.ch/pdf/sommaire2016Resumes/sommaire_3_2016/resume_ciullia.pdf
8. Alvarez Bustins G, López Plaza P-V, Carvajal SR. Profile of osteopathic practice in Spain: results from a standardized data collection study. BMC complementary and alternative medicine [Internet]. 2018 Apr 11 [cited 2019 Dec 20];18(1):129. Available from: http://www.ncbi.nlm.nih.gov/pubmed/29642901

9. Vaucher P, Macdonald RJD, Carnes D. The role of osteopathy in the Swiss primary health care system: A practice review. Vol. 8, BMJ Open. BMJ Publishing Group; 2018.

10. Carreiro J FC. Foundations of Osteopathic Medicine. Third. Chila A editor, editor. Philadelphia: Wolters Kluwer/Lippincott Williams & Wilkins; 2011. 46–52.

11. Forum for Osteopathic Regulation in Europe (FORE). Regulation. 2018.

12. Senate of Republic of Italy. Law 3/2018. Rome: Senate of Republic of Italy ; 2018.

13. Sciomachen P, … CA-I, 2018 undefined. Core competencies in osteopathy: Italian register of osteopaths proposal. journalofosteopathicmedicine.com [Internet]. [cited 2019 Dec 20]; Available from: https://www.journalofosteopathicmedicine.com/article/S1746-0689(18)30035-X/abstract

14. Johnson SM, Kurtz ME. Conditions and diagnoses for which osteopathic primary care physicians and specialists use osteopathic manipulative treatment. Journal of the American Osteopathic Association. 2002;102(10):527–40.

15. Sibbritt D, Adams J, Young AF. A profile of middle-aged women who consult a chiropractor or osteopath: findings from a survey of 11,143 Australian women. Journal of manipulative and physiological therapeutics [Internet]. 2006 Jun [cited 2019 Dec 20];29(5):349–53. Available from: http://www.ncbi.nlm.nih.gov/pubmed/16762661

16. Snider KT, Snider EJ, DeGooyer BR, Bukowski AM, Fleming RK, Johnson JC. Retrospective medical record review of an osteopathic manipulative medicine hospital consultation service. The Journal of the American Osteopathic Association [Internet]. 2013 Oct [cited 2019 Dec 20];113(10):754–67. Available from: http://www.ncbi.nlm.nih.gov/pubmed/24084802

17. Fawkes CA, Leach CMJ, Mathias S, Moore AP. A profile of osteopathic care in private practices in the United Kingdom: A national pilot using standardised data collection. Manual Therapy. 2014;19(2):125–30.

18. Morin C, Aubin A. Primary reasons for osteopathic consultation: A prospective survey in Quebec. Vol. 9, PLoS ONE. Public Library of Science; 2014.
19. Channell MK, Wang Y, McLaughlin MH, Ciesielski J, Pomerantz SC. Osteopathic Manipulative Treatment for Older Patients: A National Survey of Osteopathic Physicians. The Journal of the American Osteopathic Association [Internet]. 2016 Mar [cited 2019 Dec 20];116(3):136–43. Available from: http://www.ncbi.nlm.nih.gov/pubmed/26927907
20. Adams J, Sibbritt D, Steel A, Peng W. A workforce survey of Australian osteopathy: analysis of a nationally-representative sample of osteopaths from the Osteopathy Research and Innovation Network (ORION) project. BMC health services research [Internet]. 2018 [cited 2019 Dec 20];18(1):352. Available from: http://www.ncbi.nlm.nih.gov/pubmed/29747647
21. Rosen MA, DiazGranados D, Dietz AS, Benishek LE, Thompson D, Pronovost PJ, et al. Teamwork in healthcare: Key discoveries enabling safer, high-quality care. The American psychologist [Internet]. [cited 2019 Dec 20];73(4):433–50. Available from: http://www.ncbi.nlm.nih.gov/pubmed/29792459
22. Bennett C, Khangura S, Brehaut JC, Graham ID, Moher D, Potter BK, et al. Reporting guidelines for survey research: An analysis of published guidance and reporting practices. Vol. 8, PLoS Medicine. 2011.
23. Giusti R. Glossary of Osteopathic Terminology. Third. American association of colleges of osteopathic medicine, editor. American association of colleges of osteopathic medicine; 2017. 15.
24. (US) I of M. Educating for the Health Team: Report of the Conference on the Interrelationships of Educational Programs for Health Professionals, October 2-3, 1972. 1972 [cited 2019 Dec 20]; Available from: https://books.google.com/books?hl=it&lr=&id=8GArAAAAYAAJ&oi=fnd&pg=PP10&dq=%E2%97%8F%09Institute+of+Medicine.+(1972).+Educating+for+the+health+team.+

Washington,+DC:+National+Academy+of+Sciences&ots=BXtJVenum7&sig=dtHtWQmQE6CFmWDOg5ri8J4iF1Q

25. Institute of Medicine Committee on the Health Professions Education. The core competencies needed for health care professionals. In: A. C. Greiner & E. Knebel (Eds.), editor. Health professions education: A bridge to quality. Washington, DC: Washington, DC: National Academies Press.; 2003. p. 45–73.

26. World Health Organization. Framework for action on interprofessional education and collaborative practice. Geneva, Switzerland; 2010.

27. Berwick DM, Nolan TW, Whittington J. The triple aim: Care, health, and cost. Vol. 27, Health Affairs. 2008. p. 759–69.

28. Interprofessional Education Collaborative (2011). Core Competencies for Interprofessional Collaborative Practice [Internet]. 2011 [cited 2019 Nov 10]. Available from: https://www.aacom.org/docs/default-source/insideome/ccrpt05-10-11.pdf?sfvrsn=77937f97_2

29. Nandan M, Scott P. An innovative interprofessional education model to engage community and nonclinical participants. Journal of interprofessional care [Internet]. 2014 Jul [cited 2019 Dec 20];28(4):376–8. Available from: http://www.ncbi.nlm.nih.gov/pubmed/24617846

30. Miccolo MA, Spanier AH. Critical care management in the 1990s. Making collaborative practice work. Critical care clinics [Internet]. 1993 Jul [cited 2019 Dec 20];9(3):443–53. Available from: http://www.ncbi.nlm.nih.gov/pubmed/8353783

31. Hojat M, Fields SK, Veloski JJ, Griffiths M, Cohen MJM, Plumb JD. Psychometric properties of an attitude scale measuring physician-nurse collaboration. Evaluation and the Health Professions. 1999 Jun;22(2):208–20.

32. Whitehead D. Applying collaborative practice to health promotion. Vol. 15, Nursing standard (Royal College of Nursing (Great Britain) : 1987). 2001. p. 33–7.

33. King O, Nancarrow SA, Borthwick AM, Grace S. Contested professional role boundaries in health care: A systematic review of the literature. Journal of Foot and Ankle Research. 2015 Feb 5;8(1).

Click here to access/download
Supporting Information
supplementary material.docx
