# Supplementary material for: The Italian Osteopathic Practitioners Estimates and RAtes (OPERA) study: How osteopaths work
Source: PLoS One. 2020 Jul 2;15(7):e0235539. doi: 10.1371/journal.pone.0235539 (PMC7332305; doi:10.1371/journal.pone.0235539)
Supplement: Attachment — Submitted filename: PONE-D-19-35428_R1_reviewer.pdf [file pone.0235539.s004.pdf]

PLOS ONE

The Italian Osteopathic Practitioners Estimates and RAtes (OPERA) study: how osteopaths work.

--Manuscript Draft--

**Manuscript Number:**	PONE-D-19-35428R1
**Article Type:**	Research Article
**Full Title:**	The Italian Osteopathic Practitioners Estimates and RAtes (OPERA) study: how osteopaths work.
**Short Title:**	The Italian Osteopathic Practitioners Estimates and RAtes (OPERA) study: how osteopaths work.
**Corresponding Author:**	Giacomo Consorti<br>Centre pour l'Etude la Recherche et la Diffusion Osteopathique<br>Rome, ITALY
**Keywords:**	osteopath; scope of practice; Cross-sectional; osteopathic practice; OPERA; OPERA-IT
**Abstract:**	The scope of practice of the osteopathic profession in Italy is underreported. The first part of the present study investigated the Italian osteopaths' profile, focusing on the socio-demographic information and geographical distribution together with the main characteristics of their education. The OPERA-IT study highlighted that the majority of respondents declared to work as sole practitioners (58.4%), while the remaining declared to work as part of a team. Since teamwork and networking are recognized as fundamental aspects of healthcare, the present study aims to compare the osteopathic practice, diagnostic and treatment modalities of osteopaths who work as a sole practitioner and osteopaths who work as part of a team to highlight possible differences. Moreover, patients' characteristics will be presented.<br>The OPERA-IT study population was chosen to provide a representative sample. A web campaign was set up to inform the Italian osteopaths before the beginning of the study. The OPERA IT study used a previously tested questionnaire. The questionnaire was translated into Italian following the World Health Organization recommendation. The questionnaire was composed of 57 items grouped in five sections, namely: socio-demographics, osteopathic education and training, working profile, organization, and management of the clinical practice and patient profile. The survey was delivered online through a dedicated platform.<br>The survey was completed by 4,816 individuals. Osteopaths who work as sole practitioners represented the majority of the sample (n=2814; 58.4%). Osteopaths who work as part of a team declared to collaborate mostly with physiotherapists (n=1121; 23.3%), physicians with speciality (n=1040; 21.6%), and other osteopaths (n=943; 19.6%). The two groups showed heterogeneous characteristics. Significative differences were observed in all the factors, namely: geographical distribution, age, gender, training, working contract and working place, daily consultations and time for each consultation, fees, and the average waiting period to book an appointment. The principal component analysis supported a ten-component model and explained 80.5% of the total variance. The analysis showed that osteopaths working as sole practitioners have an increased probability (OR = 0.91; CI 95%: 0.88 - 0.94; p<0.01) of using systemic diagnostic and treatment techniques and have distinct clinical features with higher probability (OR =0.92; 0.88 - 0.96; p<0.01) of spending less time with patients, being paid less but treating a higher number of patients per week. The most represented patients' age groups were 41-64 years old (n=4452; 92.4%) and 21-40 years old (n=4291; 89.1%). Similarly, the most reported new patients' age groups were 41-64 years old (n=4221; 87.7%) and 21-40 years old (n=3364; 69.9%). The most common presenting complaints were back pain, neck pain, cervical radiculopathy, sciatica, shoulder pain, and headaches.<br>Osteopathic practice in Italy seems to be characterised by interprofessional collaboration, mostly with physiotherapists. Our results highlighted two different profiles in terms of sociodemographic characteristics and work modalities between osteopaths who work as sole practitioners and those who work as part of a team. The majority of patients are adults and most of them have been referred to osteopathy by other patients or acquaintances. Patients seek osteopathic care mostly for musculoskeletal

Powered by Editorial Manager® and ProduXion Manager® from Aries Systems Corporation

	related complaints.
**Order of Authors:**	Francesco Cerritelli
	Giacomo Consorti
	Patrick LS van Dun
	Jorge E Esteves
	Paola Sciomachen
	Massimo Valente
	Eleonora Lacorte
	Nicola Vanacore
**Response to Reviewers:**	Review of the manuscript<br><br>Manuscript number PONE-D-19-35428, entitled “The Italian Osteopathic Practitioners Estimates and RAtes (OPERA) study: how osteopaths work”<br><br><br><br>Dear editor,<br>Dear reviewers,<br><br>We greatly appreciate your readiness to have read our paper and to provide us with relevant feedback and useful suggestions to further improve the quality of our paper. A detailed description of all changes has been provided below.<br><br>For any further information, please do not hesitate to contact us.<br><br><br><br>Editor’s comments<br><br>Journal Requirements:<br>When submitting your revision, we need you to address these additional requirements:<br>1. Please ensure that your manuscript meets PLOS ONE's style requirements, including those for file naming. The PLOS ONE style templates can be found at http://www.plosone.org/attachments/PLOSOne_formatting_sample_main_body.pdf and http://www.plosone.org/attachments/PLOSOne_formatting_sample_title_authors_affiliations.pdf<br>Response: Thank you, done<br><br>2. We note that you have indicated that data from this study are available upon request. PLOS only allows data to be available upon request if there are legal or ethical restrictions on sharing data publicly. For information on unacceptable data access restrictions, please see http://journals.plos.org/plosone/s/data-availability#loc-unacceptable-data-access-restrictions.<br>In your revised cover letter, please address the following prompts:<br>a) If there are ethical or legal restrictions on sharing a de-identified data set, please explain them in detail (e.g., data contain potentially identifying or sensitive patient information) and who has imposed them (e.g., an ethics committee). Please also provide contact information for a data access committee, ethics committee, or other institutional body to which data requests may be sent.<br>b) If there are no restrictions, please upload the minimal anonymized data set necessary to replicate your study findings as either Supporting Information files or to a stable, public repository and provide us with the relevant URLs, DOIs, or accession numbers. Please see http://www.bmj.com/content/340/bmj.c181.long for guidelines on how to de-identify and prepare clinical data for publication. For a list of acceptable repositories, please see http://journals.plos.org/plosone/s/data-availability#loc-recommended-repositories.<br>Response: There was an error during the submission process, all the relevant data are available in the manuscript. In any case please refer to the revised version of the cover letter.

3. One of the noted authors is a group or consortium OPERA-IT group. In addition to naming the author group, please list the individual authors and affiliations within this group in the acknowledgments section of your manuscript. Please also indicate clearly a lead author for this group along with a contact email address.
Response: The list of authors are presented in the Acknowledgments. The lead author is Francesco Cerritelli and was included as suggested

4. Please include captions for your Supporting Information files at the end of your manuscript, and update any in-text citations to match accordingly. Please see our Supporting Information guidelines for more information:
http://journals.plos.org/plosone/s/supporting-information.
Response: All the relevant data are available in the manuscript.

Reviewer 2

Reviewer #2: Thank you for the opportunity to review this important research. I have some suggestions to strengthen the article.
1. In general, it is best to avoid starting a sentence with numeric characters (e.g. 4916 or 90%). Instead, you would need to restructure the sentence so it does not start with a numeric value or write the number in words (e.g. ninety percent).

Response: Thank you for your suggestion. The manuscript has been changed accordingly.

2. Line 71: What is osteopathy "growing' from? What is the evidence it is "growing"? Are you referring to an increase in number of practitioners? Or consultations? or something else?

Response: Thank you for your suggestion. We rephrased the sentence to make it less prone to interpretation as follow: “Osteopathy is a widespread health profession in Italy”.

3. Lines 152-158: the recruitment process needs to be clarified. What are the 12 steps? What 'other sources of information' are you referring to? Other contacts for osteopaths?

Response: Thank you for your comments. We added an example of what we meant with “different sources ”(e.g. promotional databases for healthcare professionals)” and we rephrased the promotional strategy sentence as follow: “The promotion strategy consisted of the dispatch of the e-flyer to all the different mailing lists”.

4. Results: this entire section needs English editing - particularly the first section "Comparison between osteopaths who work alone and associated". The term 'associated' is not self-evident and should probably be revised.

Response:
Thank you for the comment. The section has been reviewed and improved for clarity

5. There is no need to dedicate so much of the discussion to repeating the results. Further, the attempts to contextualise the discussion with external research is evident, but not entirely successful. Lines 331-338 are sentences without a paragraph. While Lines 341-365 appear to be dedicated to one body of work. Meanwhile, the significance of many of the key findings (as outlined in your conclusion) have not been positioned alongside existing relevant research.

Response: Thank you for your comment. Discussion have been changed accordingly.

Reviewer 3

Reviewer #3: Thank you for the opportunity to review this manuscript. Overall the premise of the work is interesting however there are some significant limitations with the statistical analysis and the description of the results. Further, there is little discussion of the work in the Discussion section of the manuscript. I have made comments and suggestions throughout the attached version of the manuscript and I

Powered by Editorial Manager® and ProduXion Manager® from Aries Systems Corporation

hope that the authors find these useful in revising the work.

The outcomes of the previous OPERA study should be described in the Introduction as they appear to be pertinent to the current study. There also needs to be greater consideration of other European studies and what they describe as the profile of osteopaths in those countries.

Response: Thanks for your advice. We provided more detailed information both on OPERA and on the other EU and international studies.

It would be valuable to describe who these participants are. Are they member of the general public?

Response: Thank you for your comment. The missing information has been added as follow “In a recent national opinion survey conducted on a sample of 800 participants from the general public by Eumetra Monterosa “

It is not clear here as to the purpose of this sentence. It makes reference to a previous study by describes the current work as the "present study". It may be better to remove this sentence however.

Response: Thank you for your comment, the term “present study” has been replaced by “OPERA study”

Regulation is also in New Zealand and Australia.

Response: Thank you for your comment, we listed just the European countries since it gives a more accurate picture of the specific context.

Please clarify what is meant by "proper" in this context.

Response: Thank you for your comment, the term “proper” has been replaced by “official”

What do these studies generally suggest are the main reasons for consultation with an osteopath? Other common characteristics across jurisdictions?

Response: Thank you for your comment, a brief report of the primary reasons for osteopathic consultation reported in those studies has been added.

Please provide some examples of the type of health professional they work with

Response: Thank you for your comment, an example has been added.

Additional references here would also be useful. One reference for a fundamental aspect of healthcare is likely insufficient.

Response: Thank you for your comment, more references supporting the concept have been added.

Please clarify is this in relation to practicing alone or with others.

Response: Thank you for your comment, the sentence has been removed because it was not pertinent.

Please provide additional detail here about the recrutiment of participants to the OPERA-IT study population. How was it determined that this was a representative sample?
It would also be valuable to clarify if the recruitment is different to the 2019 OPERA study. At present, the manuscript reads as though there is a different recruitment strategy for the current work.

Response: Thank you for your comment. We clarified that the data were collected from

	the same database used in the previous study. So the data collection was only 1 for both studies. Furthermore, we specified that "the theoretical representativeness" were addressed through the eligibility criteria.<br><br>It would appear that this is the entire OPERA-IT sample? Please clarify how these would be inclusion criteria for the current work.<br><br>Response: Thank for your comment. As per the comment above we clarified that the database was the same.<br><br>Were these people eligible to be in the OPERA-IT database?<br><br>Response: Thank for your question. Those criteria are the very same of the OPERA-IT study. We added few examples to clarify the statement.<br><br>Please ensure that the terminology is consistent throughout. Osteopath, osteopathic practitioner, osteopathic professional.<br><br>Response: Thanks for your comment. done<br><br>Please clarify what this abbreviation refers to.<br><br>Response: Thanks for your comment. done<br><br>Please provide the dates for this here.<br><br>Response: Thanks for your comment. done<br><br>Not necessarily "validated" but has been used and reported on previously. This does not constitute validation.<br><br>Response: Thanks for your comment. We rephrased accordingly<br><br>Assuming this is the World Health Organisation?<br><br>Response: Good guess! We added an explanation of the abbreviation.<br><br>Please provide a rationale for the use of relative risk over an odds ratio - the latter being more common in study designs such as the current one, particularly if logistic regression is used. RRs are not able to be used in logistic regression.<br><br>Response: Thank you for this comment. Erroneously the relative risk was included in the methods section but then in the results the odds ratio was used as suggested. Thus, we corrected the methods accordingly.<br><br>The purpose of the PCA in relation to the study is not entirely clear here. What was the purpose of identifying the components that comprised the questionnaire given that a number of variables are reported here? How was a score created for each component to be entered into the regression model?<br><br>Response: The following sentence was added in the methods section "PCA was used as a method to reduce the number of variables by extracting important elements from the large pool of variables we collected. This process aims to retain as much information as possible bringing out strong patterns in a dataset. The patterns were, then, identified in the three major areas based on similarities of variables." Concerning the score, the explanation was detailed in the section PCA and logistic regression.<br><br>It would be valuable to provide a rationale for the use of the components in the logistic regression versus the individual items on the questionnaire. The process of the logistic regression also need to be described so readers can understand how the model was built.<br><br>Response: A detailed description was added and summarised as follows: by transforming a large set of variables into a smaller one that still contains most of the

information of the large set, we could include, indeed, the majority of the variables into the logistic regression. On the contrary, if we did not use this approach, this process could not have been taken as the excessive number of variables would not be statistically appropriate to be included in the analysis.
The logistic model was also included

Please clarify the purpose of these groupings given that a PCA is to be performed.

Response: Thank you, PCA and logistic regression section was improved accordingly.

This would just be missing data rather than attrition.

Response: Well, actually the 196 questionnaires that were incomplete, that is participants started but then not finished, can be referred to as attrition, or better respondent attrition.

They also appear to be reported in Table 2?

Response: Thank you for your comment. The sentence has been deleted

It would be useful to ensure that the terminology is consistent throughout. Either 'collaborations' or 'associated'

Response: Thanks for your comment. done

Given this, a reader may ask about the value of the PCA. The components being used in the logistic regression may lose the nuance in the data.

Response: Thank you for the comment. Please refer to the previous amendments. Hopefully we improved the methods section in order to clarify better this point

Relative risk was described in the statistical analysis section however ORs are reported here. Please clarify.

Response: Thank you. Correction made

This doesn't appear to be a complete sentence.

Response: Thanks for your comment. The sentence has been rephrased

Why was 'north-west' chosen as the exposure variable?

Response: It was arbitrarily chosen but based on the rationale that the north-west region was the most representative in terms of number of osteopaths

It may not be necessary to report the ORs that are not significant and where the CI crosses 1

Response: Thank you for the comment. However, it might be useful to have a full spectrum of the data as they might be useful for further studies. Indeed, it is true that we need to refer to the statistically significant values, but the direction of effect might be a useful element to report.

Please clarify the meaning of T1 and T2 here as most readers will not understand this.

Response: Thanks for your comment. done

Assuming this should be 6?

Response: Thanks for your comment. Well...yes. My apologies.

How do these relate to the working relationship with other health professionals? If this is background for the reader, it may be better placed either in the beginning of the results.

Response: Thank you for your comment. The paragraph has been moved at the beginning of results.

Please clarify the basis on which the sample is considered to be nationally representative.

Response: Thank for your comment. We added an explanatory sentence in the method to clarify why we address the sample as "representative".
"The sample size was arbitrarily estimated and measured summing all practitioners owning a Diploma in Osteopathy or equivalent released from an Italian or an international osteopathic educational institution up to December 2016. That provided an estimated 5,100 osteopaths sample. Considering a standard deviation of 10%, the number of osteopaths in Italy was expected to range from 4,600 to 5,600. Assuming a response rate between 10 and 60 percent of those receiving the questionnaire the number of osteopaths taking part in the survey was estimated to be between 460 and 3,300."

Please clarify this part of the sentence. Is it referring to geographical distribution?

Response: Thank you for your comment. Done.

This aspect of the paragraph is likely not required as it is already part of the Methods.

Response: Thank you for your comment. The sentence has been deleted.

it would be useful to include the reference to the original study here.

Response: Thank you for your comment. Done.

Not sure if 'might' is the best word here. The work certainly contributes to the understanding of Italian osteopathic practice.

Response: Thank you for your comment. Changed accordingly.

These sentences could be removed as the essentially restating what is already in the Introduction and Method

Response: Thank you for your comment. Deleted.

Please clarify if the exposure variable is 'alone'? If so, then these osteopaths are 8% more likely. It would be difficult to categorically state they are not delivering these aspects of practice.

Response: The exposure variable is type of practice (sole practitioner vs group of practice), thus the discussion focuses on the comparison between the two groups. Therefore, the 8% is relative to the group of practice as compared to the alone [which was considered the reference category]. Then it is more likely that they are using those aspects but it does not imply they do not use them.

As per the comment above about the exposure variable, the descriptions should be in relation to the exposure variable.

Response: Please see the comment above

These are all reasonable statements but they need to be described in the context of the current work.

Response: Thank you for your comment. Discussion has been changed accordingly.

As above, these paragraphs need to be described in the context of the findings of the study.

Response: Thank you for your comment. Discussion have been changed accordingly.

Powered by Editorial Manager® and ProduXion Manager® from Aries Systems Corporation

	This should be related to working alone or with<br><br>Response: Thank you for your comment. The reported data refers to the whole sample.<br><br>Which findings of the current study are relevant here?<br><br>Response: Thank you for your comment. We specified.<br><br>These are reasonable comments however it is not clear how they relate to the current study.<br><br>Response: Thank you for your comment. Discussion have been changed accordingly.<br><br>We hope that our answers and the revision of our manuscript is meeting your expectations. We want to thank the reviewers again for providing us with the feedback and useful suggestions.<br><br>Sincerely,<br><br>The authors
**Additional Information:**	
**Question**	**Response**
**Financial Disclosure**<br><br>Enter a financial disclosure statement that describes the sources of funding for the work included in this submission. Review the submission guidelines for detailed requirements. View published research articles from *PLOS ONE* for specific examples.<br><br>This statement is required for submission and **will appear in the published article** if the submission is accepted. Please make sure it is accurate.	FC received 1 grant from Registro degli Osteopati d'Italia https://www.registro-osteopati-italia.com/<br><br>The funders had no role in study design, data collection and analysis, decision to publish, or preparation of the manuscript.

Powered by Editorial Manager® and ProduXion Manager® from Aries Systems Corporation

**Unfunded studies**<br>Enter: *The author(s) received no specific funding for this work.*<br><br>**Funded studies**<br>Enter a statement with the following details:<br>• Initials of the authors who received each award<br>• Grant numbers awarded to each author<br>• The full name of each funder<br>• URL of each funder website<br>• Did the sponsors or funders play any role in the study design, data collection and analysis, decision to publish, or preparation of the manuscript?<br>• **NO** - Include this sentence at the end of your statement: *The funders had no role in study design, data collection and analysis, decision to publish, or preparation of the manuscript.*<br>• **YES** - Specify the role(s) played.<br><br>* typeset	
**Competing Interests**<br><br>Use the instructions below to enter a competing interest statement for this submission. On behalf of all authors, disclose any competing interests that could be perceived to bias this work—acknowledging all financial support and any other relevant financial or non-financial competing interests.<br><br>This statement **will appear in the published article** if the submission is accepted. Please make sure it is accurate. View published research articles from *PLOS ONE* for specific examples.	The authors have declared that no competing interests exist.

**NO authors have competing interests**<br><br>Enter: *The authors have declared that no competing interests exist.*<br><br>**Authors with competing interests**<br><br>Enter competing interest details beginning with this statement:<br><br>*I have read the journal's policy and the authors of this manuscript have the following competing interests: [insert competing interests here]*<br><br>* typeset	
**Ethics Statement**<br><br>Enter an ethics statement for this submission. This statement is required if the study involved:<br><br>• Human participants<br>• Human specimens or tissue<br>• Vertebrate animals or cephalopods<br>• Vertebrate embryos or tissues<br>• Field research<br><br>Write "N/A" if the submission does not require an ethics statement.<br><br>General guidance is provided below. Consult the submission guidelines for detailed instructions. **Make sure that all information entered here is included in the Methods section of the manuscript.**	Participants were requested to read and understand all the information about the study and to give their informed consent by starting the survey as clearly stated in the survey presentation page. The study received the approval of the Institutional Review Board of the Foundation COME Collaboration (12/2016).

**Format for specific study types**<br><br>**Human Subject Research (involving human participants and/or tissue)**<br>• Give the name of the institutional review board or ethics committee that approved the study<br>• Include the approval number and/or a statement indicating approval of this research<br>• Indicate the form of consent obtained (written/oral) or the reason that consent was not obtained (e.g. the data were analyzed anonymously)<br><br>**Animal Research (involving vertebrate animals, embryos or tissues)**<br>• Provide the name of the Institutional Animal Care and Use Committee (IACUC) or other relevant ethics board that reviewed the study protocol, and indicate whether they approved this research or granted a formal waiver of ethical approval<br>• Include an approval number if one was obtained<br>• If the study involved *non-human primates*, add *additional details* about animal welfare and steps taken to ameliorate suffering<br>• If anesthesia, euthanasia, or any kind of animal sacrifice is part of the study, include briefly which substances and/or methods were applied<br><br>**Field Research**<br><br>Include the following details if this study involves the collection of plant, animal, or other materials from a natural setting:<br>• Field permit number<br>• Name of the institution or relevant body that granted permission	
**Data Availability**<br><br>Authors are required to make all data underlying the findings described fully available, without restriction, and from the time of publication. PLOS allows rare exceptions to address legal and ethical concerns. See the PLOS Data Policy and FAQ for detailed information.	Yes - all data are fully available without restriction

A Data Availability Statement describing where the data can be found is required at submission. Your answers to this question constitute the Data Availability Statement and **will be published in the article**, if accepted.<br><br>**Important:** Stating 'data available on request from the author' is not sufficient. If your data are only available upon request, select 'No' for the first question and explain your exceptional situation in the text box.<br><br>Do the authors confirm that all data underlying the findings described in their manuscript are fully available without restriction?	
**Describe where the data may be found in full sentences. If you are copying our sample text, replace any instances of XXX with the appropriate details.**<br><br>• If the data are **held or will be held in a public repository**, include URLs, accession numbers or DOIs. If this information will only be available after acceptance, indicate this by ticking the box below. For example: *All XXX files are available from the XXX database (accession number(s) XXX, XXX.*).<br>• If the data are all contained **within the manuscript and/or Supporting Information files**, enter the following: *All relevant data are within the manuscript and its Supporting Information files.*<br>• If neither of these applies but you are able to provide **details of access elsewhere**, with or without limitations, please do so. For example:<br><br>*Data cannot be shared publicly because of [XXX]. Data are available from the XXX Institutional Data Access / Ethics Committee (contact via XXX) for researchers who meet the criteria for access to confidential data.*<br><br>*The data underlying the results presented in the study are available from (include the name of the third party*	All relevant data are available in the manuscript

and contact information or URL).<br>• This text is appropriate if the data are owned by a third party and authors do not have permission to share the data.<br><br>* typeset	
Additional data availability information:	

Manuscript
Click here to access/download;Manuscript;R1 - Revised Manuscript clean.docx

The Italian Osteopathic Practitioners Estimates and RAtes (OPERA) study: how osteopaths work.

Francesco Cerritelli[1^], Giacomo Consorti[1,2,6], Patrick L. S. van Dun[3], Jorge E. Esteves[4,5], Paola Sciomachen[6], Massimo Valente[1], Eleonora Lacorte[7], Nicola Vanacore[7], on behalf of the OPERA-IT Group^

Affiliations

1. Clinical-based Human Research Department, Foundation COME Collaboration, Pescara, Italy
2. Research Department of the Centre pour l'Etude, la Recherche et la Diffusion Osteopathiques (C.E.R.D.O.), Rome, Italy
3. Belgium National Centre, Foundation COME Collaboration, Mechelen, Belgium
4. Gulf National Centre, Foundation COME Collaboration, Riyadh, KSA,
5. University College of Osteopathy, London, United Kingdom
6. Registro degli Osteopati d'Italia, Milan, Italy
7. National Institute of Health, Rome, Italy

¶ Membership of the OPERA-IT group is provided in the Acknowledgments.

Corresponding author

mail: giacomo.consorti@gmail.com

Abstract

The scope of practice of the osteopathic profession in Italy is underreported. The first part of the present study investigated the Italian osteopaths' profile, focusing on the socio-demographic information and geographical distribution together with the main characteristics of their education. The OPERA-IT study highlighted that the majority of respondents declared to work as sole practitioners (58.4%), while the remaining declared to work as part of a team. Since teamwork and networking are recognized as fundamental aspects of healthcare, the present study aims to compare the osteopathic practice, diagnostic and treatment modalities of osteopaths who work as a sole practitioner and osteopaths who work as part of a team to highlight possible differences. Moreover, patients' characteristics will be presented.

The OPERA-IT study population was chosen to provide a representative sample. A web campaign was set up to inform the Italian osteopaths before the beginning of the study. The OPERA IT study used a previously tested questionnaire. The questionnaire was translated into Italian following the World Health Organization recommendation. The questionnaire was composed of 57 items grouped in five sections, namely: socio-demographics, osteopathic education and training, working profile, organization, and management of the clinical practice and patient profile. The survey was delivered online through a dedicated platform.

The survey was completed by 4,816 individuals. Osteopaths who work as sole practitioners represented the majority of the sample (n=2814; 58.4%). Osteopaths who work as part of a team declared to collaborate mostly with physiotherapists (n=1121; 23.3%), physicians with speciality (n=1040; 21.6%), and other osteopaths (n=943; 19.6%). The two groups showed heterogeneous characteristics. Significative differences were observed in all the factors, namely: geographical distribution, age, gender, training, working contract and working place, daily consultations and time for each consultation, fees, and the average waiting period to book an appointment. The principal component analysis supported a ten-component model and

explained 80.5% of the total variance. The analysis showed that osteopaths working as sole practitioners have an increased probability (OR = 0.91; CI 95%: 0.88 - 0.94; p<0.01) of using systemic diagnostic and treatment techniques and have distinct clinical features with higher probability (OR =0.92; 0.88 - 0.96; p<0.01) of spending less time with patients, being paid less but treating a higher number of patients per week. The most represented patients' age groups were 41-64 years old (n=4452; 92.4%) and 21-40 years old (n=4291; 89.1%). Similarly, the most reported new patients' age groups were 41-64 years old (n=4221; 87.7%) and 21-40 years old (n=3364; 69.9%). The most common presenting complaints were back pain, neck pain, cervical radiculopathy, sciatica, shoulder pain, and headaches.

Osteopathic practice in Italy seems to be characterised by interprofessional collaboration, mostly with physiotherapists. Our results highlighted two different profiles in terms of sociodemographic characteristics and work modalities between osteopaths who work as sole practitioners and those who work as part of a team. Although according to the respondents, people of all ages consult Italian osteopaths, the majority of patients are adults. Most of them have been referred to osteopathy by other patients or acquaintances. Patients seek osteopathic care mostly for musculoskeletal related complaints.

Introduction

Osteopathy is a widespread health profession in Italy. In a recent national opinion survey conducted on a sample of 800 participants from the general public by Eumetra Monterosa[1], it has been reported that over 10 million Italians received osteopathic care, particularly for musculoskeletal related problems (70% of the reported reasons of the consultation). Ninety per cent of the sample in the study ~~declared to be~~ satisfied with the osteopathic care provided[1]. The first part of the OPERA study investigated the Italian osteopaths' ~~profile~~, focusing on the socio-demographic information and geographical distribution together with the main characteristics of their education[2]. The scope of practice of osteopathy in Italy is, however, significantly underreported. Therefore, other healthcare professionals and the general public may not be aware of the nature of the osteopathic practice, including commonly treated clinical conditions, therapeutic interventions, and patients' characteristics. This is particularly important because the osteopathic care provided may vary amongst individual clinicians and between countries [3-9]. For example, American osteopathic physicians have a scope of practice equivalent to medical practitioners [10]. In Europe, Denmark, Finland, France, Iceland, Italy, Liechtenstein, Malta, Portugal, Switzerland, Turkey, and the UK have regulated osteopathy [11]. In contrast to their US counterparts - i.e., 'osteopathic physicians', European osteopaths have limited practice rights, and they are called 'osteopaths' [10]. In Italy, with the approval of the law 3/2018, osteopathy has been recognized as a healthcare profession [12]. However, the regulation process is still ongoing, and despite the recent publication of the Core Competence of the Italian Osteopaths [13], the official scope of practice of Italian osteopaths has not yet been published.

Van Dun et al. [6] were the first authors to profile the osteopaths in countries without statutory regulation in osteopathy using the Benelux Osteosurvey tool. In their study about one-third of

respondents were women. The vast majority of respondents were self-employed in private practice and they declared to visit an average of 9 patients a day spending 30 to 60 minutes with each patient. The five most commonly reported used treatment techniques were: articulatory/mobilisation, visceral, soft and connective tissue techniques, cranial and high velocity low amplitude (HVLA) techniques. The Osteopathic Practitioners Estimates and RAtes (OPERA) project was developed starting from the Benelux Osteosurvey tool. OPERA is a European-based census aimed to profile the osteopathic profession across Europe (2). Arguably, it is a relevant tool for all the stakeholders interested in obtaining up-to-date and reliable information regarding the geo-distribution, prevalence, incidence, and profile of osteopaths and their patients in Europe. The OPERA study has been initially conducted in Italy [2] and is currently being carried out in Spain, Andorra, Belgium, Luxembourg, Portugal and Austria. Several studies investigated the primary reasons for consultation and the characteristics of patients receiving osteopathic care [5,8,14–20]. The most commonly reported reasons for osteopathic consultation were musculoskeletal complaints [8,9,17,18,20], in particular spinal complaints [8,9,17,18,20]. The aim of the OPERA Italy (OPERA-IT) study was to profile osteopathic practice in Italy by surveying osteopaths across the country regarding socio-demographic information, their practice and patients' characteristics, presenting symptoms and clinical problems, use of diagnostic and treatment modalities. The OPERA-IT study showed the profile of Italian osteopaths to be one of a young self-employed male, usually working as a sole practitioner, qualified as an osteopath through a part-time program with an earlier degree mainly in sports science or physiotherapy [2]. ~~Nevertheless, 41.6% of~~ respondents ~~stated to~~ work as part of a team with other professionals (especially physiotherapist and medical specialists). As teamwork and networking are recognized as fundamental aspects of healthcare [21-23], this study aims to compare the characteristics of osteopathic practice and the diagnostic and

treatment modalities of osteopaths working as sole practitioners and those working as part of a team.

Methods

The SUrvey Reporting GuidelinE (SURGE) [24] was used as a reporting guideline for this article.

Population

The data of the present study were collected from the OPERA-IT database [2]. The sample size was arbitrarily estimated and measured, summing all practitioners in the possession of a Diploma in Osteopathy or equivalent released from an Italian or an international osteopathic educational institution up to December 2016. That provided an estimated 5,100 osteopaths sample. Considering a standard deviation of 10%, the number of osteopaths in Italy was expected to range from 4,600 to 5,600. Assuming a response rate between 10 and 60 per cent of those receiving the questionnaire the number of osteopaths taking part in the survey was estimated to be between 460 and 3,300. The recruitment strategy followed specific criteria and was as inclusive as possible without compromising the theoretical representativeness of the sample. Hence, the recruitment was aimed to obtain the highest possible participation among those who fulfilled the following inclusion criteria: older than 18 years old, the successful completion of any training leading to a Diploma in Osteopathy (DO) or equivalent [25], and the participants had to be practising as an osteopath. Participation or successful completion of any sole training courses on single techniques and osteopathic approaches (e.g. cranial techniques course; high velocity low amplitude techniques course; biodynamic approach course), which did not lead to a DO or equivalent title [25], was not considered sufficient to be included in the study. Therefore, individuals matching this profile were excluded. Exclusion criteria were set to prevent non-osteopaths who attended short and non-degree/professional awarding courses

to participate and to lower the representativeness of the sample. OPERA-IT used an online survey; therefore, professionals with no access to the online platform were excluded. Individuals who could not understand and respond in Italian and individuals with physical or mental impairments that precluded participation in the online survey were also excluded. Participants were requested to read and understand all the information about the study and to give their informed consent by starting the survey as clearly stated in the survey presentation page. The study received the approval of the Institutional Review Board of the Foundation COME Collaboration (12/2016).

Recruitment

A website for promoting OPERA-IT was created. A web campaign was set up to inform the Italian osteopaths before the beginning of the study. The campaign was structured as a combined social media and newsletter strategy. The largest osteopathic national voluntary registering body (Italian Register of Osteopaths; ROI) took part in the promotion by sending a newsletter to all its current members. At the time in which the data gathering was carried out (February to June 2017), ROI included approximately 2,500 members. Since it was estimated that the ROI members alone were not representative of the Italian osteopaths' population, an additional e-campaign was established to reach the osteopathic education institutions, the other voluntary registering bodies and professional associations and the known osteopathic internet providers/specialised websites (i.e., tuttosteopatia.it) asking them to advertise the study to all of their members through the official OPERA IT e-flyer. In addition to the e-flyer, all the participating osteopathic education institutions were provided with a physical flyer and other advertising material to be displayed at their location. Furthermore, a manual based search on white-pages was conducted to identify other sources of information (e.g. promotional databases for healthcare professionals). The promotion strategy consisted of the dispatch of the e-flyer to

all the different mailing lists. The time interval for the promotion strategy, recruitment, and data collection was five-months. All participants, upon the completion of the survey, received an invitation containing the credential to attend free continuous professional development (CPD) webinars on a dedicated online platform. Participants were able to log in at any time during the study period and follow the pre-recorded webinars.

Survey tool

The OPERA-IT study used a questionnaire already used and reported in a previous study [6]. The questionnaire was translated into Italian following the World Health Organization (WHO) recommendation. Therefore, a forward-backwards translation was performed by two bilingual English-Italian translators with experience in the field of demographic health research. The questionnaire is composed of 57 items grouped in five sections, namely: socio-demographics, osteopathic education and training, working profile, organisation, and management of the clinical practice and patient profile. A pilot survey was delivered to twenty Italian-speaking osteopaths. The pilot aimed to gather information about the degree of comprehensibility of the items. For that purpose face-to-face interviews were conducted by the research team and the survey was modified in accordance with the suggestions of the participants. The first OPERA-IT publication reported the results of the first three sections of the survey [2]. The present study will report the results from the remaining two sections.

The OPERA survey online platform, the symmetric keys data encryption, and the certified data centre were the same used for the first part of the present study [2]. Therefore, all of the gathered information was processed and hosted following data protection regulations, the answers were anonymised, and the IP addresses were not accessible to the research team. The system automatically managed the link between the StudyID and the email address of respondents so

that double response was not allowed. Only OPERA research personnel had access to the complete, anonymised dataset.

Privacy

The anonymity and privacy of data were respected following the European directive 2002/58/CE of the European Parliament. Gathered data will be stored for 5 years to allow benchmarking and further analyses.

Statistical analysis

Data were analysed using mean, median, mode, point estimates, range, standard deviation, and 95% confidence interval. For dichotomous measures, odds ratio (OR) was used. Statistical analyses were based on a univariate and multivariate approach. R statistical programme (v. 3.1.3) was used to perform statistical analysis. A value of alpha less than 0.05 was considered as significant.

Principal-Component Analysis (PCA) and logistic analysis

The examination of the data indicated that items had non-normal distributions, which is common for categorical data. Categorical PCA, a form of PCA specifically geared to discrete ordinal values, was run using R Statistical program (v3.5). The fundamental idea of PCA is to examine the matrix of item correlations to reduce the information into a smaller set of components. These components can form the basis for hypotheses about latent factors. In the presence of high intercorrelation, items are assumed to be measuring the same latent component. All items are assumed to load onto all components.

Component eigenvalues represent the relative share of total variance accounted for by that

component and can, therefore, be used to select the number of components. We selected components being greater than 1, in order to determine the dimensions underlying the pattern of interrelationships among the scores considered. Thus, reducing the number of the original variables and increasing the interpretability of the summary components. To aid interpretability, the component matrix was rotated using Promax oblique rotation, which assumes that components are correlated. Rotations are a change in the coordinate of the component solution that makes the pattern of loadings more pronounced and, therefore clearer. Components loadings, which are the correlation coefficients between the items and the identified components, are reported. The square of component loadings represents the amount of variance in the item explained by the component.

In the present study, PCA was used as a method to reduce the number of variables by extracting important elements from the large pool of variables collected. This process aims to retain as much information as possible bringing out strong patterns in a dataset. The patterns were, then, identified in major areas based on similarities of variables and used in the regression model, as detailed below.

The rationale of applying a logistic regression is based on the fact that by transforming a large set of variables into a smaller one that still contains most of the information of the large set, we could include the majority of the variables into the logistic regression. On the contrary, if an individual questionnaire item approach was applied, the logistic regression might be biased by the large number of variables to be included in the model. This process would significantly impair the quality of the statistical analysis producing unreliable results.

The resulting components of PCA were used as independent variables in a logistic regression model with the dependent variable “working as a sole practitioner” yes/no. The regression model applied to PCA was composed of all principal components that had an eigenvalue greater than 1.

The interpretation of the meaning of each factor was defined in a collaborative way among the authors. In general, all items were categorised into (1) musculoskeletal; (2) systemic; (3) clinical. Each category was characterized by a number of affine elements (clusters). The systemic category included both diagnostic items, as visceral, cranial and fascial diagnostic techniques, and treatment items, such as neurovisceral and neurolymphatic reflex techniques and fascial techniques. The musculoskeletal category included both diagnostic and treatment items, such as palpation of the position of anatomical structures, and trigger points treatment. The "clinical" category was characterized by items which describe the clinical practice of the osteopath, such as the duration and the fees of the first and follow-up clinical encounters, the average waiting period to schedule a first appointment or the number of patients per week encountered by the practitioner.

Results

The survey was completed by 4,816 individuals. A cumulative number of 196 questionnaires, corresponding to a 4% respondent attrition rate, were left uncompleted. Osteopaths who work as sole practitioners represented the majority of the sample (n=2814; 58.4%). Osteopaths who work as part of a team ~~declared to collaborate mostly~~ with physiotherapists (n=1121; 23.3%), medical specialists (n=1040; 21.6%), and other osteopaths (n=943; 19.6%). A description of osteopaths' working collaborations is presented in Table 1.

Table 1. Working collaborations of osteopaths

	N	%
Sole practitioner	2814	58.4
Part of a team	2002	41.6
Osteopath	943	19.6
GP	390	8.1
Physiotherapist	1121	23.3

Occupational therapist	74	1.5
Psychologist	746	15.5
Speech therapist	317	6.6
Dietician	671	13.9
Dentistry	433	9.0
Massage therapist	446	9.3
Physician with speciality	1040	21.6
Optometrist	162	3.4
Other	493	10.2

Patients characteristics

The most represented age groups treated within a six months period prior to the census were 41-64 years old (n=4452; 92.4%) and 21-40 years old (n=4291; 89.1%). Similarly, the most reported new patients' age groups were 41-64 years old (n=4221; 87.7%) and 21-40 years old (n=3364; 69.9%). Respondents reported that the majority of their patients were self-referred, whether this was based on advice from other patients or acquaintances. The most common body regions requiring osteopathic care were the cervical and lumbar spine. The most common presenting complaints were back pain, neck pain, cervical radiculopathy, sciatica, shoulder pain, and headaches. The majority of respondents indicated not to have a preference of specific patients groups to work with (e.g., paediatrics, athletes, artists) (n=4106; 85.26%).

Comparison between osteopaths working as sole practitioners or as part of a team

The comparison between osteopaths working as sole practitioners and osteopaths working as part of a team showed ~~significative~~ differences in the following factors: geographical distribution, age, gender, training, working contract and working place, patients per day and time for each patient, fees, as well as the average waiting period to book an appointment. In

particular, referring to the geographical distribution, osteopaths who work in the macro-region "centre" have the highest probability to work as part of a team (OR = 1.37). Younger osteopaths (20-29 years old) as compared to other age groups showed a higher chance to work as part of a team (OR of other age groups compared to the 20-29 age group < 1). Female osteopaths are 59% more likely to work in a team compared to male colleagues (OR = 1.59). Osteopaths who graduated with a full-time curriculum (T1) have a higher chance of working in a team compared to those having a part-time diploma (T2) (OR T2 vs T1 = 0.71). Osteopaths who work as self-employed in their clinic have the highest probability of working in a team with other professionals (OR. 1.23). Osteopaths who work in a university have a 77% increased probability of working in a team compared to osteopaths who work in other places (OR = 1.77). Osteopaths who have 11 to 15 clinical encounters per day and those whose clinical encounter lasts 46-60 minutes are more likely to work in a team than others (OR = 1.50 and OR = 2.01 respectively). Osteopaths who charge between 51 and 60 euros per both first consultation and follow-ups have more than double the probability to work in a team than others (OR = 2.37; OR = 2.94). Osteopaths who have a waiting period for booking between 2 and 3 weeks have almost threefold more to the likelihood of working in a team (OR = 2.93). Extensive data about the comparison between the characteristics of the two groups are available in table 2.

Table 2. Characteristics of the two groups (sole practitioner vs as part of a team).

Variable	Sole	Part of a team	p	OR (Sole/Team)*
Geographical distribution			<0.001	
North-west	883 (31.4)	610 (30.5)		
North-east	714 (25.4)	442 (22.1)		0.90 (0.77 – 1.05)
Centre	618 (21.9)	586 (29.2)		1.37 (1.18 – 1.60)
South	503 (17.9)	310 (15.5)		0.89 (0.75 – 1.06)
Islands	96 (3.4)	54 (2.7)		0.81 (0.54 – 1.15)
Age			<0.001	
20-29	527 (18.7)	518 (25.9)		
30-39	1083 (38.5)	845 (42.2)		0.79 (0.68 – 0.92)
40-49	699 (24.8)	420 (21.0)		0.61 (0.52 – 0.73)
50-59	395 (14.0)	201 (10.0)		0.52 (0.42 – 0.64)
60-65	94 (3.4)	18 (0.9)		0.19 (0.12 – 0.33)
>65	16 (0.6)	0 (0.0)		NA

Gender				
Male	1999 (71.0)	1215 (60.7)	<0.001	
Female	815 (29.0)	787 (39.3)		1.59 (1.41 – 1.79)
Training				
Full Time (T1)	851 (30.2)	758 (37.9)	<0.001	
Part-Time (T2)	1963 (69.8)	1244 (62.1)		0.71 (0.63 – 0.80)
Work				
DO employ	31 (1.1)	34 (1.7)	<0.001	
DO self-employed in own clinic	2511 (89.2)	1600 (79.9)		0.58 (0.36 – 0.95)
DO self-employed not in own clinic	272 (9.7)	368 (18.4)		1.23 (0.74 – 2.06)
Working Place				
Private practice	2510 (92.1)	1547 (77.3)	<0.001	
Clinic/hospital	482 (17.1)	510 (25.5)	<0.001	1.72 (1.49 – 1.97)
Osteopathy School	557 (19.8)	495 (24.7)	<0.001	1.44 (1.26 – 1.65)
University	79 (2.8)	86 (4.3)	0.005	1.77 (1.29 – 2.41)
Other	374 (13.3)	356 (17.8)	<0.001	1.54 (1.32 – 1.81)
Patients/day				
0-5	1396 (49.6)	867 (43.3)		
6-10	1142 (40.6)	909 (45.4)	<0.001	1.28 (1.13 – 1.45)
11-15	225 (8.0)	210 (10.5)		1.50 (1.22 – 1.85)
16-20	39 (1.4)	10 (0.5)		0.41 (0.21 – 0.83)
>20	12 (0.4)	6 (0.3)		0.81 (0.30 – 2.15)
Time/patient				
<30 minutes	57 (2.0)	23 (1.2)		
30-45 minutes	484 (17.2)	331 (16.5)	<0.001	1.69 (1.02 – 2.81)
46-60 minutes	1651 (58.8)	1338 (66.8)		2.01 (1.23 – 3.28)
>60 minutes	622 (22.1)	310 (15.5)		1.24 (0.75 – 2.04)
Fee at the first consultation				
<25 euros	27 (1.0)	11 (0.6)		
26-30 euros	73 (2.6)	23 (1.2)		0.77 (0.33 – 1.80)
31-40 euros	198 (7.0)	103 (5.2)		1.28 (0.61 – 2.68)
41-50 euros	907 (32.2)	574 (28.6)		1.55 (0.76 – 3.16)
51-60 euros	671 (23.8)	648 (32.4)	<0.001	2.37 (1.17 – 4.82)
61-70 euros	405 (14.4)	352 (17.5)		2.13 (1.04 – 4.36)
71-80 euros	285 (10.1)	163 (8.1)		1.40 (0.68 – 2.90)
81-90 euros	113 (4.1)	61 (3.1)		1.33 (0.62 – 2.85)
91-100 euros	77 (2.7)	39 (1.9)		1.24 (0.56 – 2.77)
>100 euros	58 (2.1)	28 (1.4)		1.18 (0.51 – 2.73)
Fee following consultations				
<25 euros	43 (1.5)	12 (0.60)		
26-30 euros	100 (3.5)	50 (2.50)		1.79 (0.87 – 3.70)
31-40 euros	340 (12.1)	229 (11.4)		2.41 (1.25 – 4.68)
41-50 euros	944 (33.6)	673 (33.6)		2.55 (1.34 – 4.88)
51-60 euros	676 (24.0)	555 (27.8)	<0.001	2.94 (1.54 – 5.63)
61-70 euros	370 (13.2)	292 (14.6)		2.83 (1.46 – 5.46)
71-80 euros	184 (6.6)	125 (6.3)		2.43 (1.23 – 4.80)
81-90 euros	59 (2.0)	38 (1.9)		2.31 (1.08 – 4.93)
91-100 euros	75 (2.7)	28 (1.4)		1.34 (0.62 – 2.90)
>100 euros	23 (0.8)	0 (0.00)		NA
Average waiting period				
Same day	69 (2.5)	20 (1.00)		
Within 1 week	1559 (55.4)	1136 (56.7)		2.51 (1.52 – 4.16)
Between 1 and 2 weeks	827 (29.4)	612 (30.6)	<0.001	2.55 (1.54 – 4.25)
Between 2 and 3 weeks	126 (4.5)	107 (5.3)		2.93 (1.67 – 5.13)
Between 3 and 4 weeks	97 (3.4)	62 (3.1)		2.21 (1.22 – 3.98)
> 4 weeks	136 (4.8)	65 (3.3)		1.65 (0.92 – 2.94)

Numbers are N (%). *OR (95% confidence interval) is computed for the probability of working as a sole practitioner using the first value of each variable as the reference category.

PCA and logistic analysis

The principal component analysis supported a ten-component model (Table 3), based on eigenvalues included between 6.8 (PC-1) to 1.1 (PC-10). This model explained 80.5% of the total variance and appeared interpretable and therefore was retained. Components emerging from the analysis included all items referred to the 3 categories. Few items have been found to have loading values below -0.40, whereas a distinct number of items had values above 0.30 or below -0.30. Collectively items that correlated the most were those related to the category clinical, i.e. time to patient and fees.

Following the PCA, the ten-components model was loaded into a logistic regression in order to identify those components that associated significantly with the Sole/Team dependent variable.

As shown in Table 4, the logistic analysis demonstrated that only seven factors were significantly related to being "sole". ~~This result means that those components that resulted significantly associated include items characterising the difference between being "sole or teamed" in practice.~~ Among those, there is clear evidence that osteopaths working as a sole practitioner have an increased probability (OR = 0.91; CI 95%: 0.88 - 0.94; p<0.01) of using systemic diagnostic and treatment techniques (see PC-3 items in Table 3) and have distinct clinical features with higher probability (OR =0.92; 0.88 - 0.96; p<0.01) of spending less time with patients, being paid less but treating a higher number of patients per week (see PC-6 items in Table 3).

Table 3. Principal-Component Analysis results

	PC1	PC2	PC3	PC4	PC5	PC6	PC7	PC8	PC9	PC10
Region	0.00	-0.03	**0.28**	**-0.20**	-0.08	**-0.41**	0.16	**-0.35**	0.01	**0.30**
Gender	0.00	-0.07	**0.28**	-0.01	0.06	-0.13	**0.24**	-0.09	0.14	**-0.29**
Age	0.07	**0.30**	-0.19	-0.13	-0.12	-0.07	0.01	0.30	0.07	-0.04
Training_type	-0.03	-0.23	0.13	0.07	0.12	-0.12	**0.22**	**-0.56**	-0.08	-0.03

Time for new patient	0.01	-0.11	**0.24**	0.11	**0.20**	**-0.44**	0.05	**0.22**	0.02	**0.24**
Time for returning patient	0.03	-0.08	0.26	0.08	**0.20**	**-0.40**	0.02	**0.25**	0.07	**0.29**
Fee at first consultation	-0.02	**0.30**	-0.14	**-0.25**	0.12	**-0.35**	**0.20**	-0.06	-0.11	**-0.22**
Fee at following consultation	0.00	**0.29**	-0.12	**-0.31**	0.16	**-0.34**	0.16	-0.03	-0.10	**-0.24**
Average waiting period	0.01	**0.24**	-0.10	-0.16	0.12	0.07	**0.24**	-0.05	0.17	**0.46**
N patients per working week	-0.02	**0.25**	-0.18	**-0.20**	0.06	**0.23**	**0.21**	-0.11	0.07	**0.32**
Diagnostic techniques - assessment of visceral mobility	-0.16	0.11	**0.27**	**-0.23**	-0.05	-0.04	**-0.28**	-0.14	0.11	0.04
Diagnostic techniques - assessment of the cranium (neuro- and viscerocranium)	-0.04	**0.21**	**0.35**	-0.04	0.10	0.03	-0.17	-0.01	-0.02	-0.05
Diagnostic techniques - fascial testing	-0.11	0.17	**0.28**	**-0.20**	-0.09	0.15	-0.02	0.10	0.13	-0.04
Diagnostic techniques - inspection	-0.12	0.10	-0.05	0.02	0.04	-0.02	**-0.38**	-0.06	**-0.23**	**0.21**
Diagnostic techniques - muscle function testing	-0.16	0.18	-0.07	**0.29**	0.07	-0.01	-0.08	-0.13	-0.10	0.03
Diagnostic techniques - neurolymphatic reflex tests	**-0.20**	-0.08	-0.04	**-0.24**	0.04	0.02	-0.11	0.03	**-0.24**	-0.08
Diagnostic techniques - palpation of position/structures	-0.05	0.14	0.09	**0.20**	**0.23**	0.13	0.11	**0.20**	**-0.38**	-0.04
Diagnostic techniques - palpation of movement	-0.19	0.13	-0.06	0.17	0.01	-0.12	**-0.23**	0.04	0.16	0.03
Diagnostic techniques - percussion and auscultation	**-0.24**	-0.13	-0.11	0.05	-0.10	-0.04	0.17	0.13	**0.26**	-0.09
Diagnostic techniques - tender points and trigger points	**-0.24**	-0.12	-0.11	-0.07	**0.39**	0.11	-0.07	0.04	0.17	0.00
Diagnostic techniques - classic orthopedic tests	**-0.24**	-0.06	-0.12	-0.05	**0.39**	0.04	-0.09	0.02	0.18	0.00
Diagnostic techniques - classic neurologic tests	**-0.26**	-0.12	-0.12	0.02	**0.23**	0.10	0.00	0.11	0.10	-0.06
Diagnostic techniques - Range Of Motion (ROM)	**-0.20**	-0.14	-0.04	-0.06	**0.30**	0.13	0.00	0.06	-0.09	-0.01
Diagnostic techniques - Otoscopy	-0.09	0.18	-0.13	**0.23**	0.00	-0.12	-0.13	**-0.20**	0.13	-0.16
Diagnostic techniques - urine test	-0.05	0.13	-0.13	0.12	0.04	-0.13	**-0.22**	-0.16	**0.38**	-0.19
Treatment techniques - automatic shifting and fluid body approach	0.03	**0.28**	0.18	**0.22**	**0.22**	0.16	0.16	-0.02	-0.04	0.02
Treatment techniques - fascial techniques	-0.17	0.07	0.27	-0.04	-0.08	**0.25**	0.17	-0.01	0.12	-0.08
Treatment techniques - fluid techniques	-0.17	0.13	0.11	0.15	-0.03	0.17	**0.21**	0.15	0.06	-0.04
Treatment techniques - functional techniques	-0.15	0.09	0.18	0.04	0.08	0.06	0.14	-0.08	-0.08	-0.16
Treatment techniques - GOT/TBA	**-0.23**	-0.07	-0.04	-0.02	-0.12	0.01	0.09	0.03	**-0.27**	-0.08
Treatment techniques - HVLA	**-0.23**	-0.10	-0.13	-0.17	-0.07	-0.06	-0.03	-0.09	**-0.27**	0.09
Treatment techniques - MET	**-0.22**	-0.12	-0.04	-0.05	-0.10	-0.10	-0.02	**0.22**	-0.15	-0.10
Treatment techniques - neurocranial and viscerocranial techniques	-0.16	0.12	**0.22**	-0.02	-0.07	0.00	-0.08	-0.01	-0.11	-0.03

Treatment techniques - neurovisceral and neurolymphatic reflex techniques	-0.17	**0.20**	-0.04	0.33	-0.13	-0.04	0.02	-0.03	-0.10	-0.06
Treatment techniques - percussion and vibration techniques	-0.18	0.15	0.00	0.12	**-0.22**	-0.09	-0.05	**0.21**	0.01	0.06
Treatment techniques - trigger points	**-0.23**	-0.13	-0.08	0.02	**-0.22**	-0.07	0.27	0.09	**0.21**	-0.04
Treatment techniques - Progressive Inhibition of Neuromuscular Structures (PINS)	**-0.20**	0.05	-0.05	0.16	-0.12	-0.14	0.16	0.00	-0.09	0.16
Treatment techniques - soft and connective tissue techniques	**-0.21**	-0.09	0.10	-0.12	-0.18	-0.06	0.12	-0.02	0.01	0.18
Treatment techniques - visceral manipulations	**-0.20**	0.01	**0.22**	**-0.25**	-0.16	-0.06	-0.16	-0.15	0.08	0.10
Treatment techniques - toggle-techniques	-0.16	0.03	-0.08	0.12	-0.10	-0.13	0.13	**-0.33**	-0.04	**0.29**

Factor loadings above 0.20 (positive or negative) are in bold

Table 4. Logistic Analysis of the principal components

Coefficients	Estimated	Std. Error	z value	Pr(>|z|)	OR	95% CI
(intercept)	0.35	0.03	11.84	<0.01	1.42	1.34 - 1.51
PC1	0.07	0.01	6.39	<0.01	1.08	1.05 - 1.10
PC2	0.01	0.02	0.98	0.33	1.02	0.99 - 1.05
PC3	-0.10	0.02	-5.72	<0.01	0.91	0.88 - 0.94
PC4	0.03	0.02	1.22	0.22	1.03	0.98 - 1.07
PC5	-0.03	0.02	-1.24	0.21	0.97	0.93 - 1.02
PC6	-0.09	0.02	-3.51	<0.01	0.92	0.88 - 0.96
PC7	-0.12	0.03	-4.60	<0.01	0.89	0.84 - 0.93
PC8	0.13	0.03	4.91	<0.01	1.14	1.08 - 1.21
PC9	0.07	0.03	2.47	0.01	1.07	1.02 - 1.14
PC10	0.09	0.03	2.97	<0.01	1.09	1.03 - 1.16

OR=Odds Ratio, 95%CI= 95% confidence interval

Discussion

The variables studied are part of the OPERA questionnaire, which evaluates the characteristics of the osteopathic population. The number of respondents exceeded the theoretical estimate, therefore our sample can be considered a representative national sample.

The OPERA-IT was the first national census relevant to osteopathy in Italy [2]. Data provided by the participants represent critical new findings relating to osteopathic practice and patients

characteristics that have not been observed through other national healthcare data sets (e.g. *Istituto Nazionale di Statistica, Istituto Superiore di Sanità)*. Our results highlighted two different profiles between osteopaths who work as sole practitioners and those who work as part of a team. Osteopaths who work as part of a team are significantly younger than their colleagues who work as sole practitioners. That might represent a trend of the new osteopathic generation to work as an interprofessional team with the other healthcare professionals and to recognize the added value that interprofessional care provides to the patients. If this trend ~~will continue, soon,~~ osteopaths in Italy~~,~~ might be integrated within the already existing healthcare professional teams. Emerging evidence on the added value of effective interprofessional healthcare teams has created new perspectives on interprofessional collaboration [26–28]. Interprofessional practice has been described as a process that can affect three domains in healthcare; namely, enhancing patient experience with treatment, improving population health and decreasing healthcare costs per capita [29].

Since the resources of the healthcare system are limited and since there is an increase of ageing population with numerous chronic conditions, it is required that both clinicians and non-clinician members of the healthcare team collaborate to optimize the cost/effectiveness of their intervention [30,31]. That is particularly important since, as shown in the present study results, one of the most represented categories of professionals who collaborate with the osteopaths in Italy are medical specialists. Howev[illegible] our results showed that osteopaths who work as sole practitioners have a higher probability (PC-6; 8%; $p < 0.01$) to have a shorter duration of treatment and lower treatment fees as well as to have more average patients per week (table 3). This result suggests that further investigations on the cost/effectiveness ratio of interprofessional practice in the osteopathic field are required. ~~Contemporary healthcare strategies accept interprofessional practice as an irremissible method to address complex issues.~~ While interprofessional cooperation is beneficial to both practitioners and patients [32], it

is still not fully in place [33]. In this respect, it could be beneficial for patients, osteopaths and other stakeholders if policymakers would promote the emerging trend of working as an interprofessional team during the transition of osteopathy to a healthcare profession. Whitehead [34] identified several advantages in applying interprofessional practice for the management of complex conditions. The author argued that interprofessional practice creates an environment in which the group exceeds the parts' number; common goals are set, and everyone is working towards common goals. The chance to discuss with peers highlights the strengths and weaknesses of the working group through the exchange of experiences and knowledge. This helps to break down distrust walls and reduces rivalry. Hierarchies become flatter and more accessible. Moreover, various professional experiences offer the possibility of innovative and creative activities and to identify gaps in practice. Partnerships result in a more productive way to distribute and use resources effectively. Patients can see a more positive, focused and coordinated approach to their health needs and have more faith in it. Finally, there is a higher likelihood of a more intensive and holistic approach, which is particularly relevant to osteopathic practice. The difference in the clinical approach was one of the highlighted findings of the present study. In fact, osteopaths who work as sole practitioners have an increased probability of the 8% (PC-1; $p < 0.01$) to not deliver musculoskeletal related diagnostic and treatment techniques, in particular, tender and trigger points assessment, orthopaedic tests, neurologic tests, range of motion tests, articulatory/mobilisation techniques, High Velocity and Low Amplitude techniques, Muscle Energy Techniques (table 3). Moreover, osteopaths who work as sole practitioners are 9% more likely (PC-3; $p < 0.01$) to perform systemic diagnostic and treatment techniques such as the assessment of visceral mobility, cranium assessment, fascial testing, and cranial and visceral techniques (table 3).

Whitehead [34] also highlighted different disadvantages of not engaging in interprofessional practice. The author stated that sole practitioners often act in an individualistic way. This means

that weaknesses and mistakes are not solved, and probably they are perpetuated, there is no acknowledgement of good practice, and there are no opportunities to enhance practice. Environments are competitive in a destructive way, the hierarchies are strict, and the position of power is held through manipulative and aggressive behaviour. Perspectives and attitudes are kept isolated and limited. This suppresses the dissemination of information and ideas, fostering a practitioner centred practice. In lone practice, professional groups are protective, guarded, and mistrustful, and this may lead to professional disputes [35]. The competitive climate fosters fights for resources. This might lead to a less efficient and less successful practice [34]. Moreover, the author argues that in sole practice, there is a greater likelihood of clinical, reductionist, and mechanistic treatment being provided, particularly in terms of health services.

In general, although the scope of practice of the osteopathic profession might be influenced by the regulation status, professional profile, and cultural factors related to the country, our findings confirmed a well-established trend among other relevant surveys [5,6,8,15–17,19] showing that the primary reasons for osteopathic consultation are musculoskeletal disorders usually related to the spine. This can support the development of what might start to be considered an international shared descriptive framework of the profession.

Results from the OPERA-IT might help to define the profile of the osteopathic profession through the perspective of Italian osteopaths. This could be of use in supporting the regulation process providing materials for constructive and informed discussions with policymakers and other stakeholders. Current data might be used to tailor regulatory strategies based on policy outcomes. Moreover, professional associations and registers may benefit from present study data in terms of understanding of the working modalities of their associates and to monitor the national trends of the primary reasons for the osteopathic consultation. Finally, there are advantages for osteopaths to adapt their continuous professional development to the needs of

the Italian population and to assess their practice is up to date with the current trend of the profession on the national ground.

Strengths and weaknesses of this study

To the best of our knowledge, this study is the first to highlight the differences between the clinical profile of osteopaths who work as sole practitioners and those who work as part of a team in Italy. However, it cannot be excluded that this study showed estimates that might not be completely representative of the osteopathic Italian population. Moreover, self-reporting data might be influenced by response bias. Furthermore, data reported is from a nation-wide survey and thus might not be generalisable to other socio-cultural contexts.

Conclusions

Osteopathic practice in Italy seems to be characterised by interprofessional collaboration, mostly with physiotherapists. Our results highlighted two different profiles in terms of sociodemographic characteristics and work modalities between osteopaths who work as a sole practitioner and those who work as part of a team. Although according to the respondents, people of all ages consult Italian osteopaths, the majority of patients are adults. Most of them have been referred to osteopathy by other patients or acquaintances. Patients seek osteopathic care mostly for musculoskeletal related complaints.

The findings of the present study provide valuable insights into the osteopathic profession in Italy, which might be taken into consideration during the regulation process about the professional profile of competencies of the osteopathic profession in Italy. Follow-up studies have been planned to track future changes within the osteopathic profession.

Acknowledgements

The authors sincerely thank Prof. Angelo Manfredi and Prof. Fabrizio Consorti for their help in reviewing the paper. The OPERA-IT group is composed by: Marcello Luca Marasco (ABEos), Alberto Maggiani (AIMO), Antonio Cavallaro (AISERCO), Dario Silvestri (ASOMI), Joseph Zurlo and Marco Petracca (CERDO), Mauro Fornari (CIO), Alessandro Rapisarda (CSDOI), Giacomo Lo Voi (CSOT), Liria Papa (ICOM), Sandro Tamagnini (ICOMM), Roberta Filipazzi (IEMO), Tatiana Stirpe (Meta Osteopatia), Saverio Colonna (OSCE), Alessandro Gavazzi (SOFI), Andrea Manzotti and Andrea Bergna (SOMA), Sbarbaro Marco (SSOI), Federico Franscini (APO), Guglielmo Donnaquio (Osteopatia per Bambini), Alessandro Parisi (SIOS), Massimo Valente (Tuttosteopatia), Emanuele Botti (Advanced Osteopathy), osteopatiriconosciuti.

Author Contributions

Conceptualization: Francesco Cerritelli, Patrick L. S. van Dun, Jorge E. Esteves, Paola Sciomachen, Eleonora Lacorte, Nicola Vanacore.

Data curation: Francesco Cerritelli, Giacomo Consorti.

Formal analysis: Francesco Cerritelli, Jorge E. Esteves, Giacomo Consorti.

Funding acquisition: Francesco Cerritelli.

Investigation: Francesco Cerritelli, Patrick L. S. van Dun, Jorge E. Esteves.

Methodology: Francesco Cerritelli, Patrick L. S. van Dun, Jorge E. Esteves, Giacomo Consorti, Paola Sciomachen, Eleonora Lacorte, Nicola Vanacore.

Project administration: Francesco Cerritelli, Patrick L. S. van Dun, Jorge E. Esteves, Eleonora Lacorte.

Resources: Francesco Cerritelli, Massimo Valente

Software: Francesco Cerritelli, Massimo Valente

Supervision: Francesco Cerritelli, Patrick L. S. van Dun, Jorge E. Esteves, Paola Sciomachen, Eleonora Lacorte, Nicola Vanacore, Massimo Valente

Validation: Francesco Cerritelli, Patrick L. S. van Dun, Jorge E. Esteves, Nicola Vanacore.

Visualization: Francesco Cerritelli, Jorge E. Esteves, Giacomo Consorti.

Writing – original draft: Francesco Cerritelli, Patrick L. S. van Dun, Jorge E. Esteves, Giacomo Consorti.

Writing – review & editing: Francesco Cerritelli, Patrick L. S. van Dun, Jorge E. Esteves, Giacomo Consorti, Eleonora Lacorte, Nicola Vanacore.

References

1. Eumetra Monterosa. L'opinione degli italiani sull'osteopatia. 2017.
2. Cerritelli F, van Dun PLS, Esteves JE, Consorti G, Sciomachen P, Lacorte E, Vanacore N; OPERA-IT Group. The Italian Osteopathic Practitioners Estimates and RAtes (OPERA) study: A cross sectional survey. PLoS One. 2019 Jan 25;14(1):e0211353.
3. GOsC. Results – snapshot survey 2001. London; 2001.
4. KPMG. Report A: how do osteopaths practice? Zurich; 2011.
5. Burke SR, Myers R, Zhang AL. A profile of osteopathic practice in Australia 2010-2011: a cross sectional survey. BMC Musculoskelet Disord. 2013 Aug 1;14:227.

6. Van Dun, P. L., Nicolaie, M. A., & Van Messem, A. State of affairs of osteopathy in the Benelux: Benelux Osteosurvey 2013. International Journal of Osteopathic Medicine, 2016; 20, 3-17.

7. Ciulla, L., Foucart, J., Lepers, Y., Cullus, P., & Milabi, A. K. Enquête d'opinions sur la perception de l'ostéopathie au sein de la population belge francophone. Mémoire présenté en vue de l'obtention du grade de Master en Sciences de la motricité, à finalité Ostéopathie, ULB, Belgique. 2014

8. Alvarez Bustins G, López Plaza PV, Carvajal SR. Profile of osteopathic practice in Spain: results from a standardized data collection study. BMC Complement Altern Med. 2018 Apr 11;18(1):129.

9. Vaucher P, Macdonald RJD, Carnes D. The role of osteopathy in the Swiss primary health care system: a practice review. BMJ Open. 2018 Sep 1;8(8):e023770.

10. Carreiro J FC. Foundations of Osteopathic Medicine. Third. Chila A editor, editor. Philadelphia: Wolters Kluwer/Lippincott Williams & Wilkins; 2011. 46–52.

11. Forum for Osteopathic Regulation in Europe (FORE). Regulation. 2018.

12. Senate of Republic of Italy. Law 3/2018. Rome: Senate of Republic of Italy ; 2018.

13. Sciomachen, P., Arienti, C., Bergna, A., Castagna, C., Consorti, G., Lotti, A., ... & Longobardi, M. Core competencies in osteopathy: Italian register of osteopaths proposal. International Journal of Osteopathic Medicine, 2018, 27, 1-5.https://www.journalofosteopathicmedicine.com/article/S1746-0689(18)30035-X/abstract

14. Johnson SM, Kurtz ME. Conditions and diagnoses for which osteopathic primary care physicians and specialists use osteopathic manipulative treatment. Journal of the American Osteopathic Association. 2002;102(10):527–40.

15. Sibbritt D, Adams J, Young AF. A profile of middle-aged women who consult a chiropractor or osteopath: findings from a survey of 11,143 Australian women. J Manipulative Physiol Ther. 2006 Jun;29(5):349-53.

16. Snider KT, Snider EJ, DeGooyer BR, Bukowski AM, Fleming RK, Johnson JC. Retrospective medical record review of an osteopathic manipulative medicine hospital consultation service. J Am Osteopath Assoc. 2013 Oct;113(10):754-67.

17. Fawkes CA, Leach CMJ, Mathias S, Moore AP. A profile of osteopathic care in private practices in the United Kingdom: A national pilot using standardised data collection. Manual Therapy. 2014;19(2):125–30.

18. Morin C, Aubin A. Primary reasons for osteopathic consultation: a prospective survey in Quebec. PLoS One. 2014 Sep 3;9(9):e106259. Erratum in: PLoS One. 2015;10(3):e0121180.

19. Channell MK, Wang Y, McLaughlin MH, Ciesielski J, Pomerantz SC. Osteopathic Manipulative Treatment for Older Patients: A National Survey of Osteopathic Physicians. J Am Osteopath Assoc. 2016 Mar;116(3):136-43.

20. dams J, Sibbritt D, Steel A, Peng W. A workforce survey of Australian osteopathy: analysis of a nationally-representative sample of osteopaths from the Osteopathy Research and Innovation Network (ORION) project. BMC Health Serv Res. 2018 May 10;18(1):352.

21. Rosen MA, DiazGranados D, Dietz AS, Benishek LE, Thompson D, Pronovost PJ, Weaver SJ. Teamwork in healthcare: Key discoveries enabling safer, high-quality care. Am Psychol. 2018 May-Jun;73(4):433-450.

22. Hammick M, Olckers L, Campion-Smith C. Learning in interprofessional teams: AMEE Guide no 38. Med Teach. 2009 Jan;31(1):1-12.

23. Hean S, Craddock D, Hammick M, Hammick M. Theoretical insights into interprofessional education: AMEE Guide No. 62. Med Teach. 2012;34(2):e78-101.

24. Bennett C, Khangura S, Brehaut JC, Graham ID, Moher D, Potter BK, Grimshaw JM. Reporting guidelines for survey research: an analysis of published guidance and reporting practices. PLoS Med. 2010 Aug;8(8):e1001069.

25. Giusti R. Glossary of Osteopathic Terminology. Third. American association of colleges of osteopathic medicine, editor. American association of colleges of osteopathic medicine; 2017. 15.

26. Institute of Medicine (US). Educating for the Health Team: Report of the Conference on the Interrelationships of Educational Programs for Health Professionals, 1972 Oct 2-3. National Academies.

27. Institute of Medicine Committee on the Health Professions Education. The core competencies needed for health care professionals. In: A. C. Greiner & E. Knebel (Eds.), editor. Health professions education: A bridge to quality. Washington, DC: Washington, DC: National Academies Press.; 2003. p. 45–73.

28. World Health Organization. Framework for action on interprofessional education and collaborative practice. Geneva, Switzerland; 2010.

29. Berwick DM, Nolan TW, Whittington J. The triple aim: Care, health, and cost. Vol. 27, Health Affairs. 2008. p. 759–69.

30. Schmitt M, Blue A, Aschenbrener CA, Viggiano TR. Core competencies for interprofessional collaborative practice: reforming health care by transforming health professionals' education. Acad Med. 2011 Nov;86(11):1351.

31. Nandan M, Scott P. An innovative interprofessional education model to engage community and nonclinical participants. J Interprof Care. 2014 Jul;28(4):376-8.

32. Miccolo MA, Spanier AH. Critical care management in the 1990s. Making collaborative practice work. Crit Care Clin. 1993 Jul;9(3):443-53.

33. Hojat M, Fields SK, Veloski JJ, Griffiths M, Cohen MJM, Plumb JD. Psychometric properties of an attitude scale measuring physician-nurse collaboration. Evaluation and the Health Professions. 1999 Jun;22(2):208–20.
34. Whitehead D. Applying collaborative practice to health promotion. Nurs Stand. 2001 Jan 31-Feb 6;15(20):33-7.
35. King O, Nancarrow SA, Borthwick AM, Grace S. Contested professional role boundaries in health care: A systematic review of the literature. Journal of Foot and Ankle Research. 2015 Feb 5;8(1).

Click here to access/download
Supporting Information
supplementary material.docx

The Italian Osteopathic Practitioners Estimates and RAtes (OPERA) study: how osteopaths work.

Francesco Cerritelli[1^], Giacomo Consorti[1,2,6], Patrick L. S. van Dun[3], Jorge E. Esteves[4,5], Paola Sciomachen[6], Massimo Valente[1], Eleonora Lacorte[7], Nicola Vanacore[7], on behalf of thefor the OPERA-IT Group^

Affiliations

1. Clinical-based Human Research Department, Foundation Research Division, COME Collaboration, Pescara, Italy
2. Research Department of the Centre pour l'Etude, la Recherche et la Diffusion Osteopathiques (C.E.R.D.O.), Rome, Italy
3. Belgium National Centre, Foundation COME Collaboration, Mechelen, Belgium
4. Gulf National Centre, Foundation COME Collaboration, Riyadh, KSA,
5. University College of Osteopathy, London, United Kingdom
6. Registro degli Osteopati d'Italia, Milan, Italy
7. National Institute of Health, Rome, Italy

¶ Membership of the OPERA-IT group is provided in the Acknowledgments.

Corresponding author**Address for corrispondence**

Giacomo Consorti

Largo Ceresio, 4

22100 Como (CO)

Italy

ph: +39 3405204077

E-email: giacomo.consorti@gmail.com

Abstract

~~Introduction:~~ The scope of practice of the osteopathic profession in Italy is underreported. The first part of the present study investigated the Italian osteopaths' profile, focusing on the socio-demographic information and geographical distribution together with the main characteristics of their education. The OPERA-IT study highlighted that the majority of respondents declared to work as sole practitioners~~alone~~ (58.4%), while the remaining declared to work as part of a team~~in a team~~~~association with other professionals~~. Since teamwork and networking are recognized as fundamental aspects of healthcare, the present study aims to compare the osteopathic practic~~s~~e, diagnostic~~,~~ and treatment modalities of osteopaths who work as a sole practitioner~~alone~~ and osteopaths who work as part of a team~~associated to other healthcare professionals~~ to highlight possible differences. Moreover, patients' characteristics will be presented.

~~Methods:~~ The OPERA-IT study population was chosen to provide a representative sample. A web campaign was set up to inform the Italian osteopath~~ic professional~~s before the beginning of the study. The OPERA IT study used a previously tested questionnaire. The questionnaire was translated into Italian following the World Health Organization recommendation. The questionnaire was composed of 57 items grouped in five sections, namely: socio-demographics, osteopathic education and training, working profile, organization, and management of the clinical practice and patient profile. The survey was delivered online through a dedicated platform.

~~Results:~~ The survey was completed by 4,816 individuals~~ completed the survey~~. Osteopaths who work as sole practitioners~~alone~~ represented the majority of the sample (n=2814; 58.4%). Osteopaths who work as part of a team~~with other professionals~~ declared to collaborate mostly with physiotherapists (n=1121; 23.3%), physicians with speciality (n=1040; 21.6%), and other osteopaths (n=943; 19.6%). The two groups showed heterogeneous characteristics.

Formatted: Not Highlight

Formatted: Heading 1

Formatted: Font: Not Bold

Significative differences were observed in all the factors, namely: geographical distribution, age, gender, training, working contract and working place, daily consultations and time for each consultation~~patients per day and time for each patient~~, fees, and the average waiting period to book an appointment. The principal component analysis supported a ten-component model and explained 80.5% of the total variance. The analysis showed that osteopaths working as sole practitioners~~alone~~ have an increased probability (OR = 0.91; CI 95%: 0.88 - 0.94; p<0.01) of using systemic diagnostic and treatment techniques and have distinct clinical features with higher probability (OR =0.92; 0.88 - 0.96; p<0.01) of spending less time with patients, being paid less but treating a higher number of patients per week. The most represented patients' age groups were 41-64 years old (n=4452; 92.4%) and 21-40 years old (n=4291; 89.1%). Similarly, the most reported new patients' age groups were 41-64 years old (n=4221; 87.7%) and 21-40 years old (n=3364; 69.9%). The most common presenting complaints were back pain, neck~~cervical~~ pain, cervical radiculopathy~~cervicobrachialgia~~, sciatica, shoulder pain, and headaches.

~~**Conclusions:**~~ Osteopathic practice in Italy seems to be characterised by interprofessional collaboration, mostly with physiotherapists. Our results highlighted two different profiles in terms of sociodemographic characteristics and work modalities between osteopaths who work as sole practitioners~~alone~~ and those who work as part of a team~~associated with other professionals~~. Although according to the respondents, people of all ages consult Italian osteopaths, the majority of patients are adults. Most of them have been referred to osteopathy by other patients or acquaintances. Patients seek osteopathic care mostly for musculoskeletal related complaints.

Introduction

Osteopathy is a widespread~~growing~~ health profession in Italy. In a recent national opinion survey conducted on a sample of 800 participants from the general public by Eumetra Monterosa[1], it has been reported that over 10 million Italians received osteopathic care, particularly for musculoskeletal related problems (70% of the reported reasons of the consultation). Ninety per cent~~90%~~ of the sample in the study declared to be satisfied with the osteopathic care provided[1]. The first part of the OPERA~~present~~ study investigated the Italian osteopaths' profile, focusing on the socio-demographic information and geographical distribution together with the main characteristics of their education[2]. The scope of practice of ~~the~~ osteopathy~~ic profession~~ in Italy is, however, significantly underreported. Therefore, other healthcare professionals and the general public may not be aware of the nature of the osteopathic practice, including commonly treated clinical conditions, therapeutic interventions, and patients' characteristics. This is particularly important because the osteopathic care provided may vary amongst individual clinicians and between countries [3-9]. For example, American osteopathic physicians have a scope of practice equivalent to medical practitioners [10]. In Europe, Denmark, Finland, France, Iceland, Italy, Liechtenstein, Malta, Portugal, Switzerland, Turkey, and the UK have regulated osteopathy [11]. In contrast to their US counterparts - i.e., 'osteopathic physicians', European osteopaths have limited practice rights, and they are called 'osteopaths' [10]. In Italy, with the approval of the law 3/2018, osteopathy has been recognized as a healthcare profession [12]. However, the regulation process is still ongoing, and despite the recent publication of the Core Competence of the Italian Osteopaths [13], the official~~proper~~ scope of practice of Italian osteopaths has not yet been published.

Formatted: Not Highlight

Formatted: Heading 1

Van Dun et al. [6] were the first authors to profile the osteopath~~ic practitioner~~s in countries without statutory regulation in osteopathy using the Benelux Osteosurvey tool. In their study about one-third of respondents were women. The vast majority of respondents were self-employed in private practice and they declared to visit an average of 9 patients a day spending 30 to 60 minutes with each patient. The five most commonly reported used treatment techniques were: articulatory~~artticulatory~~/mobilisation, visceral~~manipulation~~, soft and connective tissue techniques, cranial and high velocity low amplitude (HVLA) ~~-~~techniques~~ and general osteopathic mobilisations~~. The Osteopathic Practitioners Estimates and RAtes (OPERA) project was developed starting from the Benelux Osteosurvey tool. OPERA is a European-based census aimed to profile the osteopathic profession across Europe (2). Arguably, it is a relevant tool for all the stakeholders interested in obtaining up-to-date and reliable information regarding the geo-distribution, prevalence, incidence, and profile of osteopaths and their patients in Europe. The OPERA study has been initially conducted in Italy [2] and is currently being carried out in Spain, Andorra, Belgium, Luxembourg, ~~and~~ Portugal and Austria. Several studies investigated the primary reasons for consultation and the characteristics of patients receiving osteopathic care [5,8,14–20]. The most commonly reported reasons for ~~the~~ osteopathic consultation were ~~clinical~~ musculoskeletal complaints ~~presentat~~[8,9,17,18,20], ~~. I~~in particular~~,~~ ~~the disorders affecting the~~ spinal complaints ~~e anatomic region, are the most documented among clinical musculoskeletal presentations~~ [8,9,17,18,20]. ~~However, none of these studies was carried out on the Italian population.~~

The aim of the OPERA Italy (OPERA-IT) study was to profile osteopathic practice in Italy by surveying osteopaths across the country regarding socio-demographic information~~(2)~~, their practice and patients' characteristics, presenting symptoms and clinical problems, use of diagnostic and treatment modalities. The OPERA-IT study showed~~highlighted that~~ the profile of Italian osteopaths~~in Italy seems~~ to be one ~~defined by~~of a young self-employed male, usually

Formatted: Space Before: 8 pt, After: 8 pt

working mostly as a sole practitioner, who was qualified as an osteopath through via a part-time program with an earlierand had a previous degree mainly in sports science or physiotherapy [2] fields. Nevertheless, 41.6% of respondents stated to workTherefore, the majority of respondents declared to work alone (58.4%), while the remaining declared to work as part of a teamin association with other professionals (especially.g. physiotherapist and, medical specialistsphysicians with specialiststy). AsSince teamwork and networking are recognized as fundamental aspects of healthcare [21-23], thise present study aims to compare the characteristics ofchararteristics osteopathic practicse, and the diagnostic, and treatment modalities of osteopaths who working as sole practitionersalone and thoseosteopaths who working as part of a teamin association withed to other healthcare professionals. to highlight possible differences. Moreover, patients' characteristics will be presented.

Methods

The SUrvey Reporting GuidelinE (SURGE) [242] was used as a reporting guideline for this article.

Population

The data of the present study werehave been collected from tThe OPERA-IT database [2]. The sample size was arbitrarily estimated and measured, summing all practitioners in the possession ofowning a Diploma in Osteopathy or equivalent released from an Italian or an international osteopathic educational institution up to December 2016. That provided an estimated 5,100 osteopaths sample. Considering a standard deviation of 10%, the number of osteopaths in Italy was expected to range from 4,600 to 5,600. Assuming a response rate between 10 and 60 per cent of those receiving the questionnaire the number of osteopaths taking part in the survey

Formatted: Font: Not Bold

Formatted: Heading 1

was estimated to be between 460 and 3,300. The ~~study population was chosen to provide a representative sample. For that purpose, the~~ recruitment strategy followed specific criteria and was as inclusive as possible without compromising the theoretical representativeness of the sample. Hence, the recruitment was aimed to obtain the highest possible participation among those who fulfilled the following inclusion criteria: older than 18 years old, the successful completion of any training leading to a Diploma in Osteopathy (DO) or equivalent [25]~~3~~, and the participants had to be practising as an osteopath. Participation or successful completion of any sole training courses on single techniques and osteopathic approaches (e.g. cranial techniques course; high velocity low amplitude techniques course; biodynamic approach course), which did not lead to a DO or equivalent title [25]~~3~~, was not considered sufficient to be included in the study. Therefore, individuals matching this profile were excluded. Exclusion criteria were set to prevent non-osteopaths who attended short and non-degree/professional awarding courses to participate and to lower the representativeness of the sample. OPERA-IT used an online survey; therefore, professionals with no access to the online platform were excluded. Individuals who could not understand and respond in Italian and individuals with physical or mental impairments that precluded participation in the online survey were also excluded. Participants were requested to read and understand all the information about the study and to give their informed consent by starting the survey as clearly stated in the survey presentation page. The study received the approval of the Institutional Review Board of the Foundation COME Collaboration (12/2016).

Recruitment

A website for promoting OPERA-IT was created. A web campaign was set up to inform the Italian osteopath~~ic professional~~s before the beginning of the study. The campaign was structured as a combined social media and newsletter strategy. The largest osteopathic national

Formatted: Font:

Formatted: Left, Space After: 6 pt, Line spacing: Multiple 1.15 li, Keep with next, Keep lines together

voluntary registering body (Italian Register of Osteopaths; ROI) took part in the promotion by sending a newsletter to all its current members. At the time in which the data gatheringstudy was carried out (February to June 2017), ROI included approximately 2,500 members. Since it was estimated that the ROI members alone were not representative of the Italian osteopaths' population, an additional e-campaign was established to reach the osteopathic education institutions, the other voluntary registering bodies and professional associations and the known osteopathic internet providers/specialised websites (i.e., tuttosteopatia.it) asking them to advertise the study to all of their members through the official OPERA IT e-flyer. In addition to the e-flyer, all the participating osteopathic education institutions were provided with a physical flyer and other advertising material to be displayed at their location. Furthermore, a manual based search on white-pages was conducted to identify other sources of information (e.g. promotional databases for healthcare professionals). The promotion strategy was carried in twelve steps. Each step consisted of the dispatch of the e-flyer to all the different mailing lists. The time interval for the promotion strategy, recruitment, and data collection was five-months. All participants, upon the completion of the survey, received an invitation containing the credential to attend free continuous professional development (CPD) webinars on a dedicated online platform. Participants were able to log in at any time during the study period and follow the pre-recorded webinars.

Survey tool

The OPERA-IT study used a questionnaire already used and reported ion a previous studyvalidated questionnaire [6]. The questionnaire was translated into Italian following the World Health Organization (WHO) recommendation. Therefore, a forward-backwards translation was performed by two bilingual English-Italian translators with experience in the field of demographic health research. The questionnaire is composed of 57 items grouped in

Formatted: Font:

Formatted: Left, Space After: 6 pt, Line spacing: Multiple 1.15 li, Keep with next, Keep lines together

five sections, namely: socio-demographics, osteopathic education and training, working profile, organisation, and management of the clinical practice and patient profile. A pilot survey was delivered to twenty Italian-speaking osteopaths. The pilot aimed to gather information about the degree of comprehensibility of the items. For that purpose face-to-face interviews were conducted by the research team and the survey was modified in accordance with the suggestions of the participants. The first OPERA-IT publication reported the results of the first three sections of the survey [2]. The present study will report the results from the remaining two sections.

The OPERA survey online platform, the symmetric keys data encryption, and the certified data centre were the same used for the first part of the present study [2]. Therefore, all of the gathered information was processed and hosted following data protection regulations, the answers were anonymised, and the IP addresses were not accessible to the research team. The system automatically managed the link between the StudyID and the email address of respondents so that double response was not allowed. Only OPERA research personnel had access to the complete, anonymised dataset.

Privacy

The anonymity and privacy of data were respected following the European directive 2002/58/CE of the European Parliament. Gathered data will be stored for 5 years to allow benchmarking and further analyses.

~~**Information guidelines**~~

Formatted: Font:

Formatted: Left, Space After: 6 pt, Line spacing: Multiple 1.15 li, Keep with next, Keep lines together

In this study will be reported participants answers regarding their practice and patients' characteristics, presenting symptoms and clinical problems, use of diagnostic and treatment modalities

Statistical analysis

Data were analysed using mean, median, mode, point estimates, range, standard deviation, and 95% confidence interval. For dichotomous measures, odds ratio (OR)relative risk was used. Statistical analyses were based on a univariate and multivariate approach. R statistical programme (v. 3.1.3) was used to perform statistical analysis. A value of alpha less than 0.05 was considered as significant.

Formatted: Font:

Formatted: Left, Space After: 6 pt, Line spacing: Multiple 1.15 li, Keep with next, Keep lines together

Principal-Component Analysis (PCA) and logistic analysis

Formatted: Font color: Black

Formatted: Heading 3

The examination of the data indicated that items had non-normal distributions, which is common for categorical data. Categorical PCA, a form of PCA specifically geared to discrete ordinal values, was run using R Statistical program (v3.5). The fundamental idea of PCA is to examine the matrix of item correlations to reduce the information into a smaller set of components. These components can form the basis for hypotheses about latent factors. In the presence of high intercorrelation, items are assumed to be measuring the same latent component. All items are assumed to load onto all components.

Component eigenvalues represent the relative share of total variance accounted for by that component and can, therefore, be used to select the number of components. We selected components being greater than 1, in order to determine the dimensions underlying the pattern of interrelationships among the scores considered. Thus, reducing the number of the original variables and increasing the interpretability of the summary components. To aid interpretability, the component matrix was rotated using Promax oblique rotation, which

assumes that components are correlated. Rotations are a change in the coordinate of the component solution that makes the pattern of loadings more pronounced and, therefore clearer. Components loadings, which are the correlation coefficients between the items and the identified components, are reported. The square of component loadings represents the amount of variance in the item explained by the component.

In the present study, PCA was used as a method to reduce the number of variables by extracting important elements from the large pool of variables collected. This process aims to retain as much information as possible bringing out strong patterns in a dataset. The patterns were, then, identified in major areas based on similarities of variables and used in the regression model, as detailed below.

~~The resulting components of PCA were used as independent variables in a logistic regression model with the dependent variable "alone" yes/no.~~ The rationale of applying a logistic regression is based on the fact that by transforming a large set of variables into a smaller one that still contains most of the information of the large set, we could include the majority of the variables into the logistic regression. On the contrary, if an individual questionnaire item approach was applied, the logistic regression might be biased by the large number of variables to be included in the model. This process would significantly impair~~impared~~ the quality of the statistical analysis producing unreliable results.

The resulting components of PCA were used as independent variables in a logistic regression model with the dependent variable "working as a sole practitioner~~alone~~" yes/no. The regression model applied to PCA was composed of all principal components that had an eigenvalue greater than 1.

The interpretation of the meaning of each factor was defined in a collaborative way among the authors. In general, all items were categorised into (1) musculoskeletal; (2) systemic; (3) clinical. Each category was characterized by a number of affine elements (clusters). The

systemic category included both diagnostic items, as the visceral, cranialum, and fascial diagnostic techniques, and treatment items, such as neurovisceral and neurolymphatic reflex techniques and fascial techniques. The musculoskeletal category included as well both diagnostic and treatment items, such as palpation of the position of the anatomical structures, and trigger points treatment. The "clinical" category was characterized by items which describe the clinical practice of the osteopathic professional, such as the duration and the fees of the first and follow-up clinical encounters, the average waiting period to schedule a firstn appointment or the number of patients per week encountered by the practitioner.

Results

Formatted: Heading 1

The survey was completed by 4,816 individuals completed the survey. A cumulative number of 196 questionnaires, corresponding to a 4% respondent attrition rate, were left uncompleted. Composition and geographical distribution of the whole sample are reported by Cerritelli et al. (2). Osteopaths who work as sole practitionersalone represented the majority of the sample (n=2814; 58.4%). Osteopaths who work as part of a teamwith other professionals declared to collaborate mostly with physiotherapists (n=1121; 23.3%), medical specialistsphysicians with speciality (n=1040; 21.6%), and other osteopaths (n=943; 19.6%). A comprehensive description of osteopaths' working collaborations is presented in Table 1.collaborations is available in Table 1.

	N	%
Alone	2814	58.4
Associated	2002	41.6
osteopath	943	19.6

GP	390	8.1
physiotherapist	1121	23.3
occupational therapist	74	1.5
psychologist	746	15.5
speech therapist	317	6.6
dietician	671	13.9
dentistry	433	9.0
massage therapist	446	9.3
physician with specialty	1040	21.6
optometrist	162	3.4
other	493	10.2

Table 1. Working collaborations of osteopaths

Table 1. Working collaborations of osteopaths

	N	%
Sole practitioner	2814	58.4
Part of a teamgroup of practice	2002	41.6
Osteopath	943	19.6
GP	390	8.1
Physiotherapist	1121	23.3
Occupational therapist	74	1.5
Psychologist	746	15.5
Speech therapist	317	6.6
Dietician	671	13.9
Dentistry	433	9.0
Massage therapist	446	9.3
Physician with speciality	1040	21.6

Optometrist	162	3.4
Other	493	10.2

Patients characteristics

The most represented age groups treated within a six months period prior to the census were 41-64 years old (n=4452; 92.4%) and 21-40 years old (n=4291; 89.1%). Similarly, the most reported new patients' age groups were 41-64 years old (n=4221; 87.7%) and 21-40 years old (n=3364; 69.9%). Respondents reported that the majority of their patients were self-referred, whether this was based on advice from other patients or acquaintances. The most common body regions requiring osteopathic care were the cervical and lumbar spine. The most common presenting complaints were back pain, neck pain, cervical radiculopathy, sciatica, shoulder pain, and headaches. The majority of respondents indicated not to have~~ no~~ a preference of specific patients groups to work with (e.g., paediatrics, athletes, artists) (n=4106; 85.26%).

Comparison between osteopaths working as sole practitioners or as part of a team~~group of practice~~ ~~who work alone and in collaboration with other professionals~~~~associated~~

The comparison between osteopaths working as sole practitioners and osteopaths working as part of a team~~group of practice~~ showed significative differences in the following factors~~group two groups showed heterogeneous characteristics. Significative differences were observed in all the factors, namely~~: geographical distribution, age, gender, training, working contract and working place, patients per day and time for each patient, fees, as well as~~and~~ the average waiting period to book an appointment. In particular, referring to the geographical distribution, osteopaths who work in the macro-region "centre" have the highest probability to ~~ratio~~~~odd to~~ work as part of a team ~~group of practice~~~~associated with other professionals~~ (OR = 1.37).

Formatted: Font: (Default) Times New Roman, 12 pt, Bold

Formatted: Heading 2

Formatted: Font: Not Bold

Formatted: Font:

Formatted: Heading 2

Formatted: Font:

Younger osteopaths (20-29 years old) as compared to other age groups showed a higher chance to work as part of a team~~in aas group of practice~~ ~~have the highest odd to work associated compared to other age groups~~ (OR of other age groups compared to the 20-29 age group < 1). Female osteopaths are~~have~~ 59% more likely to work in ~~as~~ a team~~group of practiceassociated~~ compared to male colleagues ~~ones~~ (OR = 1.59). Osteopaths who graduated with~~through~~ a full-time curriculum (T1) have a higher chance of working in ~~a~~ a team~~group of practice~~ ~~associated~~ compared to those having ~~from~~ a part-time diploma~~one~~ (T2) (OR ~~for~~ T2 vs~~compared to~~ T1 = 0.71). Osteopaths who work as self-employed in their clinic have the highest probability of working in~~as~~ a team~~group of practicein association~~ with other professionals (OR. 1.23). Osteopaths who work in a university have a 77% increased probability of working in ~~as~~ a team~~group of practiceassociated~~ compared to osteopaths who work in other places (OR = 1.77). Osteopaths who have 11 to 15 clinical encounters per day and those whose clinical encounter lasts 46-60 minutes are more likely to work in ~~as~~ a team~~group of practicein association~~ than others (OR = 1.50 and~~;~~ OR = 2.01 respectively) ~~as well those whose clinical encounter lasts 46-60 minutes (OR = 2.01)~~. Osteopaths who charge between 51 and 60 euros per both first consultation and follow-ups have more than ~~the~~ double the probability to work in~~as~~ a team~~group of practice~~ ~~in association~~ than others (OR = 2.37; OR = 2.94). Osteopaths who have a waiting period for ~~the~~ booking ~~in~~ between 2 and 3 weeks have ~~almost a probability~~ almost threefold more to thet~~times the~~ likelihood of working in~~as~~ a team~~group of practicehigher to work in association compared to the others~~ (OR = 2.93). Extensive data about the comparison between the characteristics of the two groups are available in table 2.

Table 2. Characteristics of the two groups (sole practitioner vs as part of a team~~group of practice~~).

Variable	Sole	Part of a team~~group~~	p	OR (Sole/Team~~e/Grou~~)*

Geographical distribution				
North-west	883 (31.4)	610 (30.5)	<0.001	
North-east	714 (25.4)	442 (22.1)		0.90 (0.77 – 1.05)
Centre	618 (21.9)	586 (29.2)		1.37 (1.18 – 1.60)
South	503 (17.9)	310 (15.5)		0.89 (0.75 – 1.06)
Islands	96 (3.4)	54 (2.7)		0.81 (0.54 – 1.15)
Age				
20-29	527 (18.7)	518 (25.9)	<0.001	
30-39	1083 (38.5)	845 (42.2)		0.79 (0.68 – 0.92)
40-49	699 (24.8)	420 (21.0)		0.61 (0.52 – 0.73)
50-59	395 (14.0)	201 (10.0)		0.52 (0.42 – 0.64)
60-65	94 (3.4)	18 (0.9)		0.19 (0.12 – 0.33)
>65	16 (0.6)	0 (0.0)		NA
Gender				
Male	1999 (71.0)	1215 (60.7)	<0.001	
Female	815 (29.0)	787 (39.3)		1.59 (1.41 – 1.79)
Training				
Full Time (T1)	851 (30.2)	758 (37.9)	<0.001	
Part-Time (T2)	1963 (69.8)	1244 (62.1)		0.71 (0.63 – 0.80)
Work				
DO employ	31 (1.1)	34 (1.7)	<0.001	
DO self-employed in own clinic	2511 (89.2)	1600 (79.9)		0.58 (0.36 – 0.95)
DO self-employed not in own clinic	272 (9.7)	368 (18.4)		1.23 (0.74 – 2.06)
Working Place				
Private practice	2510 (92.1)	1547 (77.3)	<0.001	
Clinic/hospital	482 (17.1)	510 (25.5)	<0.001	1.72 (1.49 – 1.97)
Osteopathy School	557 (19.8)	495 (24.7)	<0.001	1.44 (1.26 – 1.65)
University	79 (2.8)	86 (4.3)	0.005	1.77 (1.29 – 2.41)
Other	374 (13.3)	356 (17.8)	<0.001	1.54 (1.32 – 1.81)
Patients/day				
0-5	1396 (49.6)	867 (43.3)	<0.001	
6-10	1142 (40.6)	909 (45.4)		1.28 (1.13 – 1.45)
11-15	225 (8.0)	210 (10.5)		1.50 (1.22 – 1.85)
16-20	39 (1.4)	10 (0.5)		0.41 (0.21 – 0.83)
>20	12 (0.4)	6 (0.3)		0.81 (0.30 – 2.15)
Time/patient				
<30 minutes	57 (2.0)	23 (1.2)	<0.001	
30-45 minutes	484 (17.2)	331 (16.5)		1.69 (1.02 – 2.81)
46-60 minutes	1651 (58.8)	1338 (66.8)		2.01 (1.23 – 3.28)
>60 minutes	622 (22.1)	310 (15.5)		1.24 (0.75 – 2.04)
Fee at the first consultation				
<25 euros	27 (1.0)	11 (0.6)	<0.001	
26-30 euros	73 (2.6)	23 (1.2)		0.77 (0.33 – 1.80)
31-40 euros	198 (7.0)	103 (5.2)		1.28 (0.61 – 2.68)
41-50 euros	907 (32.2)	574 (28.6)		1.55 (0.76 – 3.16)
51-60 euros	671 (23.8)	648 (32.4)		2.37 (1.17 – 4.82)
61-70 euros	405 (14.4)	352 (17.5)		2.13 (1.04 – 4.36)
71-80 euros	285 (10.1)	163 (8.1)		1.40 (0.68 – 2.90)
81-90 euros	113 (4.1)	61 (3.1)		1.33 (0.62 – 2.85)
91-100 euros	77 (2.7)	39 (1.9)		1.24 (0.56 – 2.77)
>100 euros	58 (2.1)	28 (1.4)		1.18 (0.51 – 2.73)
Fee following consultations				
<25 euros	43 (1.5)	12 (0.60)	<0.001	
26-30 euros	100 (3.5)	50 (2.50)		1.79 (0.87 – 3.70)
31-40 euros	340 (12.1)	229 (11.4)		2.41 (1.25 – 4.68)
41-50 euros	944 (33.6)	673 (33.6)		2.55 (1.34 – 4.88)
51-60 euros	676 (24.0)	555 (27.8)		2.94 (1.54 – 5.63)
61-70 euros	370 (13.2)	292 (14.6)		2.83 (1.46 – 5.46)
71-80 euros	184 (6.6)	125 (6.3)		2.43 (1.23 – 4.80)
81-90 euros	59 (2.0)	38 (1.9)		2.31 (1.08 – 4.93)
91-100 euros	75 (2.7)	28 (1.4)		1.34 (0.62 – 2.90)
>100 euros	23 (0.8)	0 (0.00)		NA

Average waiting period				
Same day	69 (2.5)	20 (1.00)		
Within 1 week	1559 (55.4)	1136 (56.7)		2.51 (1.52 – 4.16)
Between 1 and 2 weeks	827 (29.4)	612 (30.6)	<0.001	2.55 (1.54 – 4.25)
Between 2 and 3 weeks	126 (4.5)	107 (5.3)		2.93 (1.67 – 5.13)
Between 3 and 4 weeks	97 (3.4)	62 (3.1)		2.21 (1.22 – 3.98)
> 4 weeks	136 (4.8)	65 (3.3)		1.65 (0.92 – 2.94)

Numbers are N (%). *OR (95% confidence interval) is computed for the probability of working as a sole practitioner using the first value of each variable as the reference category.

Variable	Alone (%)	Associated (%)	*p*	OR (Alo/Ass)*
Geographical distribution				
North-west	883 (31.4)	610 (30.5)		
North-east	714 (25.4)	442 (22.1)		0.90 (0.77 – 1.05)
Centre	618 (21.9)	586 (29.2)	<0.001	1.37 (1.18 – 1.60)
South	503 (17.9)	310 (15.5)		0.89 (0.75 – 1.06)
Islands	96 (3.4)	54 (2.7)		0.81 (0.54 – 1.15)
Age				
20-29	527 (18.7)	518 (25.9)		
30-39	1083 (38.5)	845 (42.2)		0.79 (0.68 – 0.92)
40-49	699 (24.8)	420 (21.0)		0.61 (0.52 – 0.73)
50-59	395 (14.0)	201 (10.0)	<0.001	0.52 (0.42 – 0.64)
60-65	94 (3.4)	18 (0.9)		0.19 (0.12 – 0.33)
>65	16 (0.6)	0 (0.0)		NA
Gender				
Male	1999 (71.0)	1215 (60.7)	<0.001	
Female	815 (29.0)	787 (39.3)		1.59 (1.41 – 1.79)
Training				
Full Time (T1)	851 (30.2)	758 (37.9)	<0.001	
Part Time (T2)	1963 (69.8)	1244 (62.1)		0.71 (0.63 – 0.80)
Work				
DO employed	31 (1.1)	34 (1.7)		
DO self-employed in own clinic	2511 (89.2)	1600 (79.9)	<0.001	0.58 (0.36 – 0.95)
DO self-employed not in own clinic	272 (9.7)	368 (18.4)		1.23 (0.74 – 2.06)
Working Place				
Private practice	2510 (92.1)	1547 (77.3)	<0.001	
Clinic/hospital	482 (17.1)	510 (25.5)	<0.001	1.72 (1.49 – 1.97)
Osteopathy School	557 (19.8)	495 (24.7)	<0.001	
University	79 (2.8)	86 (4.3)	0.005	

Other	374 (13.3)	356 (17.8)	<0.001	1.44 (1.26–1.65)<br>1.77 (1.29–2.41)<br>1.54 (1.32–1.81)
Patients/day				
0-5	1396 (49.6)	867 (43.3)	<0.001	
<0.0016-10	1142 (40.6)	909 (45.4)		1.28 (1.13–1.45)
11-15	225 (8.0)	210 (10.5)		1.50 (1.22–1.85)
16-20	39 (1.4)	10 (0.5)		0.41 (0.21–0.83)
>20	12 (0.4)	6 (0.3)		0.81 (0.30–2.15)
Time/patient				
<30 minutes	57 (2.0)	23 (1.2)	<0.001	
30-45 minutes	484 (17.2)	331 (16.5)		1.69 (1.02–2.81)
46-60 minutes	1651 (58.8)	1338 (66.8)		2.01 (1.23–3.28)
>60 minutes	622 (22.1)	310 (15.5)		1.24 (0.75–2.04)
Fee first consultation				
<25 euros	27 (1.0)	11 (0.6)	<0.001	
26-30 euros	73 (2.6)	23 (1.2)		0.77 (0.33–1.80)
31-40 euros	198 (7.0)	103 (5.2)		1.28 (0.61–2.68)
41-50 euros	907 (32.2)	574 (28.6)		1.55 (0.76–3.16)
51-60 euros	671 (23.8)	648 (32.4)		2.37 (1.17–4.82)
61-70 euros	405 (14.4)	352 (17.5)		2.13 (1.04–4.36)
71-80 euros	285 (10.1)	163 (8.1)		1.40 (0.68–2.90)
81-90 euros	113 (4.1)	61 (3.1)		1.33 (0.62–2.85)
91-100 euros	77 (2.7)	39 (1.9)		1.24 (0.56–2.77)
>100 euros	58 (2.1)	28 (1.4)		1.18 (0.51–2.73)
Fee following consultations				
<25 euros	43 (1.5)	12 (0.60)	<0.001	
26-30 euros	100 (3.5)	50 (2.50)		1.79 (0.87–3.70)
31-40 euros	340 (12.1)	229 (11.4)		2.41 (1.25–4.68)
41-50 euros	944 (33.6)	673 (33.6)		
51-60 euros	676 (24.0)	555 (27.8)		

61-70 euros	370 (13.2)	292 (14.6)		2.55 (1.34 – 4.88)
71-80 euros	184 (6.6)	125 (6.3)		2.94 (1.54 – 5.63)
81-90 euros	59 (2.0)	38 (1.9)		2.83 (1.46 – 5.46)
91-100 euros	75 (2.7)	28 (1.4)		2.43 (1.23 – 4.80)
>100 euros	23 (0.8)	0 (0.00)		2.31 (1.08 – 4.93)
				1.34 (0.62 – 2.90)
				NA
Average waiting period				
Same day	69 (2.5)	20 (1.00)		
Within 1 week	1559 (55.4)	1136 (56.7)		2.51 (1.52 – 4.16)
1 week < X ≤ 2 weeks	827 (29.4)	612 (30.6)		2.55 (1.54 – 4.25)
2 weeks < X ≤ 3 weeks	126 (4.5)	107 (5.3)	<0.001	2.93 (1.67 – 5.13)
3 weeks < X ≤ 4 weeks	97 (3.4)	62 (3.1)		2.21 (1.22 – 3.98)
> 4 weeks	136 (4.8)	65 (3.3)		1.65 (0.92 – 2.94)

Table 2. characteristics of the two groups (*alone* vs *associated*).

*OR is computed for the probability of working alone using the first value of each variable as the exposure factor.

Formatted: Justified, Line spacing: single

PCA and logistic analysis

Formatted: Font:

The principal component analysis supported a ten-component model (Table 3), based on eigenvalues included between 6.8 (PC-1) to 1.1 (PC-10). This model explained 80.5% of the total variance and appeared interpretable and therefore was retained. Components emerging from the analysis included all items referred to the 3 categories. Few items have been foundhave found to have loading values below -0.40, whereas a distinct number of items had values above 0.30 or below -0.30. Collectively items that correlated the most were those related to the category clinical, i.e. time to patient and fees.

Following the PCA, the ten-components model was loaded into a logistic regression in order to identify those components that associated significantly with the SoleAlone/TeamAssociated dependent variable.

As shown in Table 4, the logistic analysis demonstrated that only seven factors were significantly related to being "solealone". This result means that those components that resulted significantly associated include items characterising the difference between being "solealone or teamedassociated" in practice. Among those, there is clear evidence that osteopaths working as a sole practitioneralone have an increased probability (OR = 0.91; CI 95%: 0.88 - 0.94; p<0.01) of using systemic diagnostic and treatment techniques (see PC-3 items in Table 3) and have distinct clinical features with higher probability (OR =0.92; 0.88 - 0.96; p<0.01) of spending less time with patients, being paid less but treating a higher number of patients per week (see PC-6 items in Table 3).

Table 3. Principal-Component Analysis results

	PC1	PC2	PC3	PC4	PC5	PC6	PC7	PC8	PC9	PC10
Region	0.00	-0.03	**0.28**	**-0.20**	-0.08	**-0.41**	0.16	**-0.35**	0.01	**0.30**
Gender	0.00	-0.07	**0.28**	-0.01	0.06	-0.13	**0.24**	-0.09	0.14	**-0.29**
Age	0.07	**0.30**	-0.19	-0.13	-0.12	-0.07	0.01	0.30	0.07	-0.04
Training_type	-0.03	-0.23	0.13	0.07	0.12	-0.12	**0.22**	**-0.56**	-0.08	-0.03
Time for new patient	0.01	-0.11	**0.24**	0.11	**0.20**	**-0.44**	0.05	**0.22**	0.02	**0.24**
Time for returning patient	0.03	-0.08	0.26	0.08	**0.20**	**-0.40**	0.02	**0.25**	0.07	**0.29**
Fee at first consultation	-0.02	**0.30**	-0.14	**-0.25**	0.12	**-0.35**	**0.20**	-0.06	-0.11	**-0.22**
Fee at following consultation	0.00	**0.29**	-0.12	**-0.31**	0.16	**-0.34**	0.16	-0.03	-0.10	**-0.24**
Average waiting period	0.01	**0.24**	-0.10	-0.16	0.12	0.07	**0.24**	-0.05	0.17	**0.46**
N patients per working week	-0.02	**0.25**	-0.18	**-0.20**	0.06	**0.23**	**0.21**	-0.11	0.07	**0.32**
Diagnostic techniques - assessment of visceral mobility	-0.16	0.11	**0.27**	**-0.23**	-0.05	-0.04	**-0.28**	-0.14	0.11	0.04
Diagnostic techniques - assessment of the cranium (neuro- and viscerocranium)	-0.04	**0.21**	**0.35**	-0.04	0.10	0.03	-0.17	-0.01	-0.02	-0.05
Diagnostic techniques - fascial testing	-0.11	0.17	**0.28**	**-0.20**	-0.09	0.15	-0.02	0.10	0.13	-0.04

Diagnostic techniques - inspection	-0.12	0.10	-0.05	0.02	0.04	-0.02	**-0.38**	-0.06	**-0.23**	**0.21**
Diagnostic techniques - muscle function testing	-0.16	0.18	-0.07	**0.29**	0.07	-0.01	-0.08	-0.13	-0.10	0.03
Diagnostic techniques - neurolymphatic reflex tests	**-0.20**	-0.08	-0.04	**-0.24**	0.04	0.02	-0.11	0.03	**-0.24**	-0.08
Diagnostic techniques - palpation of position/structures	-0.05	0.14	0.09	**0.20**	**0.23**	0.13	0.11	**0.20**	**-0.38**	-0.04
Diagnostic techniques - palpation of movement	-0.19	0.13	-0.06	0.17	0.01	-0.12	**-0.23**	0.04	0.16	0.03
Diagnostic techniques - percussion and auscultation	**-0.24**	-0.13	-0.11	0.05	-0.10	-0.04	0.17	0.13	**0.26**	-0.09
Diagnostic techniques - tender points and trigger points	**-0.24**	-0.12	-0.11	-0.07	**0.39**	0.11	-0.07	0.04	0.17	0.00
Diagnostic techniques - classic orthopedic tests	**-0.24**	-0.06	-0.12	-0.05	**0.39**	0.04	-0.09	0.02	0.18	0.00
Diagnostic techniques - classic neurologic tests	**-0.26**	-0.12	-0.12	0.02	**0.23**	0.10	0.00	0.11	0.10	-0.06
Diagnostic techniques - Range Of Motion (ROM)	**-0.20**	-0.14	-0.04	-0.06	**0.30**	0.13	0.00	0.06	-0.09	-0.01
Diagnostic techniques - Otoscopy	-0.09	0.18	-0.13	**0.23**	0.00	-0.12	-0.13	**-0.20**	0.13	-0.16
Diagnostic techniques - urine test	-0.05	0.13	-0.13	0.12	0.04	-0.13	**-0.22**	-0.16	**0.38**	-0.19
Treatment techniques - automatic shifting and fluid body approach	0.03	**0.28**	0.18	**0.22**	**0.22**	0.16	0.16	-0.02	-0.04	0.02
Treatment techniques - fascial techniques	-0.17	0.07	0.27	-0.04	-0.08	**0.25**	0.17	-0.01	0.12	-0.08
Treatment techniques - fluid techniques	-0.17	0.13	0.11	0.15	-0.03	0.17	**0.21**	0.15	0.06	-0.04
Treatment techniques - functional techniques	-0.15	0.09	0.18	0.04	0.08	0.06	0.14	-0.08	-0.08	-0.16
Treatment techniques - GOT/TBA	**-0.23**	-0.07	-0.04	-0.02	-0.12	0.01	0.09	0.03	**-0.27**	-0.08
Treatment techniques - HVLA	**-0.23**	-0.10	-0.13	-0.17	-0.07	-0.06	-0.03	-0.09	**-0.27**	0.09
Treatment techniques - MET	**-0.22**	-0.12	-0.04	-0.05	-0.10	-0.10	-0.02	**0.22**	-0.15	-0.10
Treatment techniques - neurocranial and viscerocranial techniques	-0.16	0.12	**0.22**	-0.02	-0.07	0.00	-0.08	-0.01	-0.11	-0.03
Treatment techniques - neurovisceral and neurolymphatic reflex techniques	-0.17	**0.20**	-0.04	0.33	-0.13	-0.04	0.02	-0.03	-0.10	-0.06
Treatment techniques - percussion and vibration techniques	-0.18	0.15	0.00	0.12	**-0.22**	-0.09	-0.05	**0.21**	0.01	0.06
Treatment techniques - trigger points	**-0.23**	-0.13	-0.08	0.02	**-0.22**	-0.07	0.27	0.09	**0.21**	-0.04
Treatment techniques - Progressive Inhibition of Neuromuscular Structures (PINS)	**-0.20**	0.05	-0.05	0.16	-0.12	-0.14	0.16	0.00	-0.09	0.16
Treatment techniques - soft and connective tissue techniques	**-0.21**	-0.09	0.10	-0.12	-0.18	-0.06	0.12	-0.02	0.01	0.18
Treatment techniques - visceral manipulations	**-0.20**	0.01	**0.22**	**-0.25**	-0.16	-0.06	-0.16	-0.15	0.08	0.10
Treatment techniques - toggle-techniques	-0.16	0.03	-0.08	0.12	-0.10	-0.13	0.13	**-0.33**	-0.04	**0.29**

Factor loadings above 0.20 (positive or negative) are in bold

	PC1	PC2	PC3	PC4	PC5	PC6	PC7	PC8	PC9	PC10
Region	0.00	-0.03	0.28	-0.20	-0.08	-0.41	0.16	-0.35	0.01	0.30
Gender	0.00	-0.07	0.28	-0.01	0.06	-0.13	0.24	-0.09	0.14	-0.29
Age	0.07	0.30	-0.19	-0.13	-0.12	-0.07	0.01	0.30	0.07	-0.04
Training_type	-0.03	-0.23	0.13	0.07	0.12	-0.12	0.22	-0.56	-0.08	-0.03
Time for new patient	0.01	-0.11	0.24	0.11	0.20	-0.44	0.05	0.22	0.02	0.24
Time for returning patient	0.03	-0.08	0.26	0.08	0.20	-0.40	0.02	0.25	0.07	0.29
Fee first consultation	-0.02	0.30	-0.14	-0.25	0.12	-0.35	0.20	-0.06	-0.11	-0.22
Fee following consultation	0.00	0.29	-0.12	-0.31	0.16	-0.34	0.16	-0.03	-0.10	-0.24
Average waiting period	0.01	0.24	-0.10	-0.16	0.12	0.07	0.24	-0.05	0.17	0.46
N patients per working week	-0.02	0.25	-0.18	-0.20	0.06	0.23	0.21	-0.11	0.07	0.32
Diagnostic techniques – assessment of visceral mobility	-0.16	0.11	0.27	-0.23	-0.05	-0.04	-0.28	-0.14	0.11	0.04
Diagnostic techniques – assessment of the cranium (neuro- and viscerocranium)	-0.04	0.21	0.35	-0.04	0.10	0.03	-0.17	-0.01	-0.02	-0.05
Diagnostic techniques – fascial testing	-0.11	0.17	0.28	-0.20	-0.09	0.15	-0.02	0.10	0.13	-0.04
Diagnostic techniques – inspection	-0.12	0.10	-0.05	0.02	0.04	-0.02	-0.38	-0.06	-0.23	0.21
Diagnostic techniques – muscle function testing	-0.16	0.18	-0.07	0.29	0.07	-0.01	-0.08	-0.13	-0.10	0.03

**Diagnostic techniques – neurolymphatic reflex tests**	**-0.20**	-0.08	-0.04	**-0.24**	0.04	0.02	-0.11	0.03	**-0.24**	-0.08
**Diagnostic techniques – palpation of position/structures**	-0.05	0.14	0.09	**0.20**	**0.23**	0.13	0.11	**0.20**	**-0.38**	-0.04
**Diagnostic techniques – palpation of movement**	-0.19	0.13	-0.06	0.17	0.01	-0.12	**-0.23**	0.04	0.16	0.03
**Diagnostic techniques – percussion and auscultation**	**-0.24**	-0.13	-0.11	0.05	-0.10	-0.04	0.17	0.13	**0.26**	-0.09
**Diagnostic techniques – tender points and trigger points**	**-0.24**	-0.12	-0.11	-0.07	**0.39**	0.11	-0.07	0.04	0.17	0.00
**Diagnostic techniques – classic orthopedic tests**	**-0.24**	-0.06	-0.12	-0.05	**0.39**	0.04	-0.09	0.02	0.18	0.00
**Diagnostic techniques – classic neurologic tests**	**-0.26**	-0.12	-0.12	0.02	**0.23**	0.10	0.00	0.11	0.10	-0.06
**Diagnostic techniques – Range Of Motion (ROM)**	**-0.20**	-0.14	-0.04	-0.06	**0.30**	0.13	0.00	0.06	-0.09	-0.01
**Diagnostic techniques – Otoscopy**	-0.09	0.18	-0.13	**0.23**	0.00	-0.12	-0.13	**-0.20**	0.13	-0.16
**Diagnostic techniques – urine test**	-0.05	0.13	-0.13	0.12	0.04	-0.13	**-0.22**	-0.16	**0.38**	-0.19
**Treatment techniques – automatic shifting and fluid body approach**	0.03	**0.28**	0.18	**0.22**	**0.22**	0.16	0.16	-0.02	-0.04	0.02
**Treatment techniques – fascial techniques**	-0.17	0.07	**0.27**	-0.04	-0.08	**0.25**	0.17	-0.01	0.12	-0.08
**Treatment techniques – fluid techniques**	-0.17	0.13	0.11	0.15	-0.03	0.17	**0.21**	0.15	0.06	-0.04
**Treatment techniques – functional techniques**	-0.15	0.09	0.18	0.04	0.08	0.06	0.14	-0.08	-0.08	-0.16
**Treatment techniques – GOT/TBA**	**-0.23**	-0.07	-0.04	-0.02	-0.12	0.01	0.09	0.03	**-0.27**	-0.08
**Treatment techniques – HVLA**	**-0.23**	-0.10	-0.13	-0.17	-0.07	-0.06	-0.03	-0.09	**-0.27**	0.09

~~Treatment techniques - MET~~	**–0.22**	–0.12	–0.04	–0.05	–0.10	–0.10	–0.02	**0.22**	–0.15	–0.10
~~Treatment techniques - neurocranial and viscerocranial techniques~~	–0.16	0.12	**0.22**	–0.02	–0.07	0.00	–0.08	–0.01	–0.11	–0.03
~~Treatment techniques - neurovisceral and neurolymphatic reflex techniques~~	–0.17	**0.20**	–0.04	**0.33**	–0.13	–0.04	0.02	–0.03	–0.10	–0.06
~~Treatment techniques - percussion and vibration techniques~~	–0.18	0.15	0.00	0.12	**–0.22**	–0.09	–0.05	**0.21**	0.01	0.06
~~Treatment techniques - trigger points~~	**–0.23**	–0.13	–0.08	0.02	**–0.22**	–0.07	**0.27**	0.09	**0.21**	–0.04
~~Treatment techniques - Progressive Inhibition of Neuromuscular Structures (PINS)~~	**–0.20**	0.05	–0.05	0.16	–0.12	–0.14	0.16	0.00	–0.09	0.16
~~Treatment techniques - soft and connective tissue techniques~~	**–0.21**	–0.09	0.10	–0.12	–0.18	–0.06	0.12	–0.02	0.01	0.18
~~Treatment techniques - visceral manipulations~~	**–0.20**	0.01	**0.22**	**–0.25**	–0.16	–0.06	–0.16	–0.15	0.08	0.10
~~Treatment techniques - toggle techniques~~	–0.16	0.03	–0.08	0.12	–0.10	–0.13	0.13	**–0.33**	–0.04	**0.29**

~~Factor loadings above 0.20 (positive or negative) are in bold~~

Formatted: Justified, Line spacing: Double

~~Table 3. Principal Component Analysis results~~
Table 4. Logistic Analysis of the principal components

Coefficients	Estimated	Std. Error	z value	Pr(>|z|)	OR	95% CI
(intercept)	0.35	0.03	11.84	<0.01	1.42	1.34 - 1.51
PC1	0.07	0.01	6.39	<0.01	1.08	1.05 - 1.10
PC2	0.01	0.02	0.98	0.33	1.02	0.99 - 1.05
PC3	-0.10	0.02	-5.72	<0.01	0.91	0.88 - 0.94
PC4	0.03	0.02	1.22	0.22	1.03	0.98 - 1.07
PC5	-0.03	0.02	-1.24	0.21	0.97	0.93 - 1.02
PC6	-0.09	0.02	-3.51	<0.01	0.92	0.88 - 0.96
PC7	-0.12	0.03	-4.60	<0.01	0.89	0.84 - 0.93
PC8	0.13	0.03	4.91	<0.01	1.14	1.08 - 1.21
PC9	0.07	0.03	2.47	0.01	1.07	1.02 - 1.14
PC10	0.09	0.03	2.97	<0.01	1.09	1.03 - 1.16

OR=Odds Ratio, 95%CI= 95% confidence interval

Coefficients	Estimated	Std. Error	z value	Pr(>|z|)	OR	95% CI
**(intercept)**	0.35	0.03	11.84	**<0.01**	1.42	1.34 - 1.51
**PC1**	0.07	0.01	6.39	**<0.01**	1.08	1.05 - 1.10
**PC2**	0.01	0.02	0.98	0.33	1.02	0.99 - 1.05
**PC3**	-0.10	0.02	-5.72	**<0.01**	0.91	0.88 - 0.94
**PC4**	0.03	0.02	1.22	0.22	1.03	0.98 - 1.07
**PC5**	-0.03	0.02	-1.24	0.21	0.97	0.93 - 1.02
**PC6**	-0.09	0.02	-3.51	**<0.01**	0.92	0.88 - 0.96
**PC7**	-0.12	0.03	-4.60	**<0.01**	0.89	0.84 - 0.93
**PC8**	0.13	0.03	4.91	**<0.01**	1.14	1.08 - 1.21
**PC9**	0.07	0.03	2.47	**0.01**	1.07	1.02 - 1.14
**PC10**	0.09	0.03	2.97	**<0.01**	1.09	1.03 - 1.16

Table 4. Logistic Analysis of the principal components

Patients characteristics

The most represented age groups treated within a six months period prior to the census were 41-64 years old (n=4452; 92.4%) and 21-40 years old (n=4291; 89.1%). Similarly, the most reported new patients age groups were 41-64 years old (n=4221; 87.7%) and 21-40 years old (n=3364; 69.9%). Respondents reported that the majority of their patients were self-referred, whether this was based on advice from other patients or acquaintances. The most common body regions requiring osteopathic care were the cervical and lumbar spine. The most common presenting complaints were back pain, cervical pain, cervicobrachialgia, sciatica, shoulder pain, and headaches. The majority of respondents indicated to have no preference of specific patients groups to work with (e.g., paediatrics, athletes, artists) (n=4106; 85.26%).

Discussion

The variables studied are part of the OPERA questionnaire, which evaluates the characteristics of the osteopathic population. The number of respondents exceeded the theoretical estimate, therefore our sample can be considered in a representative national sample. After an initial evaluation of their geographical distribution (2), the resultsscores were modulated with a statistical analysis procedure, in order to further identify the profile of the osteopathic

Formatted: Font: (Default) Times New Roman, 12 pt

Formatted: Font: Not Bold

Formatted: Font:

Formatted: Font: Not Bold

practitioner with peculiar characteristics (components), which allowed better defining the profiles of the studied population.

The OPERA-IT was the first national census relevant to osteopathy in Italy [2]. Data provided by the participants might represent critical new findings relating to osteopathic practice and patients characteristics that have not been observed through other national healthcare data sets (e.g. *Istituto Nazionale di Statistica, Istituto Superiore di Sanità*). The results of this study provide a comparison between the osteopathic professionals who work alone and those who work in association with other professionals to highlight possible differences in terms of geographical distribution, age, gender, type of training, working place and modalities, patients per day, time of the treatment, fees, and average waiting period for booking an appointment. Moreover, it describes patients' characteristics in terms of age, referring modalities. Our results highlighted two different profiles between osteopaths who work as sole practitionersalone and those who work as part of a teamassociated with other professionals. The former have an increased probability of the 8% (PC-1; $p < 0.01$) to not deliver musculoskeletal related diagnostic and treatment techniques, in particular, tender and trigger points assessment, orthopaedic tests, neurologic tests, range of motion tests, General Osteopathic Treatment (GOT), High Velocity and Low Amplitude techniques (HVLA), Muscle Energy Techniques (MET) (table 3). Moreover, osteopaths who work alone are 9% more likely (PC-3; $p < 0.01$) to perform systemic diagnostic and treatment techniques such as the assessment of visceral mobility, cranium assessment, fascial testing, and cranial and visceral manipulations (table 3). Osteopaths who work in association with other professionals have a higher probability (PC-6; 8%; $p < 0.01$) to have a short duration of the treatment and low treatment fees and to have more average patients per week (table 3). Osteopaths who works as part of a teamin association with other professionals are significantly younger than their colleagues who work as sole practitionersalone. That might represent a trend of the new osteopathic generation to work as

an interprofessional team with the other healthcare professionals and to recognize the added value that interprofessional care provides to the patients. If this trend will continue, soon, osteopaths in Italy, might be integrated within the already existing healthcare professional teams.

-Emerging evidence on the added value of effective interprofessional healthcare teams has created new perspectives on interprofessional collaboration [264–286].

Interprofessional practice has been described as a process that can affect three domains in healthcare; namely, enhancing patient experience with treatment, improving population health and decreasing healthcare costs per capita [297].

Since the resources of the healthcare system are limited and since there is an increase ofCapacity shortages, an ageing population with numerous chronic conditions and new scientific discoveries, it is required thatthe cooperation of both clinicians and non-cliniciancal members of the healthcare team collaborate to optimize the cost/effectiveness of their intervention [OPTIMIZE 3028,31]29. That is particularly important since, as shown in the present study results, one of the most represented categoriescategory of professionals who collaborate with the osteopaths in Italy are medical specialists. physiciansphysician with speciality. However, our results showed that osteopaths who work as sole practitioners have a higher probability (PC-6; 8%; $p < 0.01$) to have a shorter duration of the treatment and lower treatment fees as well asand to have more average patients per week (table 3). This result suggests that further investigations on the cost/effectiveness ratio of interprofessional practice in the osteopathic field are requiredthe service.

Contemporary healthcare strategies accept interprofessional practice as an irremissible method to address complex issues. While interprofessional cooperation is beneficial to both practitioners and patients [320], it is still not fully in place [331]. In this respectOn that point of view, it couldmay be beneficial for patients, osteopaths andthe other stakeholders, ifthe policymakers

would~~ill~~ promote~~foster~~ the emerging trend of working as an interprofessional team during the~~osteopathy~~ transition of osteopathy~~in~~ to a healthcare~~into healthcare~~ profession. Whitehead [34]~~[42]~~ identified several advantages in applying interprofessional practice for the management of complex conditions. The author argued that interprofessional practice creates an environment in which the group exceeds the parts' number; common goals are set, and everyone is working towards common goals.~~;~~ T~~t~~he chance to discuss with peers highlights the strengths and weaknesses of the working group through the exchange of experiences and knowledge. This helps to break down distrust walls and reduces rivalry. Hierarchies become flatter and more accessible.

Moreover, various professional experiences offer the possibility of innovative and creative activities and to identify gaps in practice.~~;~~ P~~partnerships and Pp~~artnerships result in a more productive way to distribute and use resources effectively.~~;~~ P~~p~~atients can see a more positive, focused and coordinated approach to their health needs and have more faith in it. Finally, there is a higher likelihood of a more~~of more~~ intensive and holistic approach, which is particularly relevant to osteopathic practice. The difference in the clinical approach was one of the highlighted findings of the present study. In fact, osteopaths who work as sole practitioners have an increased probability of the 8% (PC-1; $p < 0.01$) to not deliver musculoskeletal related diagnostic and treatment techniques, in particular, tender and trigger points assessment, orthopaedic tests, neurologic tests, range of motion tests, articulatory/mobilisation techniques~~General Osteopathic Treatment (GOT)~~, High Velocity and Low Amplitude techniques, Muscle Energy Techniques (table 3). Moreover, osteopaths who work as sole practitioners are 9% more likely (PC-3; $p < 0.01$) to perform systemic diagnostic and treatment techniques such as the assessment of visceral mobility, cranium assessment, fascial testing, and cranial and visceral techniques~~manipulations~~ (table 3).

Whitehead [34~~2~~] also highlighted different disadvantages of not engaging in interprofessional practice. The author stated that sole~~lone~~ practitioners often act in an individualistic way. This means that weaknesses and mistakes are not solved, and probably they are perpetuated, there is no acknowledgement of good practice, and there are no opportunities to enhance practice. Environments are competitive in a destructive way, the hierarchies are strict, and the position of power is held through manipulative and aggressive behaviour. Perspectives and attitudes are kept isolated and limited. This suppresses the dissemination of information and ideas, fostering a practitioner centred practice. In lone practice, professional groups are protective, guarded, and mistrustful, and this may lead to professional disputes [35~~3~~]. The competitive climate fosters fights for resources. This might lead to a less efficient and less successful practice [34~~2~~]. Moreover, the author argues that in sole~~lone~~ practice, there is a greater likelihood of clinical, reductionist, and mechanistic treatment being provided, particularly in terms of health services. ~~Our findings confirmed a well-established trend among other relevant surveys (5,6,8,15–17,19) showing that primary reasons for osteopathic consultation are musculoskeletal disorders mostly related to the spine.~~

In general, although the scope of practice of the osteopathic~~of osteopathic~~ profession might be influenced by the regulation status, professional profile, and cultural factors related to the country, o~~ur study found several similarities with the other European and international surveys.~~ ~~O~~ur findings confirmed a well-established trend among other relevant surveys [5,6,8,15–17,19] showing that the primary reasons for osteopathic consultation are musculoskeletal disorders usually~~mostly~~ related to the spine. This can support~~help~~~~Our findings supported some of the already known trends about the scope of practice of osteopathy (e.g. , helping~~ the development of~~to strengthen~~ what might start to be considered an international shared descriptive framework of the profession.

Results from the OPERA-IT might help to define the profile of the osteopathic~~of osteopathic~~ profession~~als~~ through the perspective of Italian osteopaths. This could be of use in supporting the regulation process providing materials for constructive and informed discussions with policymakers and other stakeholders. Current data might be used to tailor regulatory strategies based on policy outcomes. Moreover, professional associations and registers may benefit from present study data in terms of understanding of the working modalities of their associates and to monitor the national trends of the primary reasons for the osteopathic consultation. ~~Lastl~~Finally, there are advantages for osteopath~~ic practitioner~~s~~:~~ to adapt~~be able to tailor~~ their continuous professional development to the need~~ing~~s of the Italian population and to assess their practice is up to date with the current trend of the profession on the national ground.

Strengths and weaknesses of this study

Formatted: Font:

Formatted: Heading 2

To the best of our knowledge, this study is the first to highlight the differences between the clinical profile of osteopath~~ic practitioner~~s who work as sole practitioners~~alone~~ and those who work as part of a team~~in association with other professionals~~ in Italy. However, it cannot be excluded that this study showed estimates that might not be completely representative of the osteopathic Italian population. Moreover, self-reporting data might be influenced by response bias. Furthermore, data reported is from a nation-wide survey and thus might not be generalisable to other socio-cultural contexts.

Conclusions

Formatted: Font:

Formatted: Heading 1

Osteopathic practice in Italy seems to be characterised by interprofessional collaboration, mostly with physiotherapists. Our results highlighted two different profiles in terms of

sociodemographic characteristics and work modalities between osteopaths who work as a sole practitioner~~alone~~ and those who work as part of a team~~associated with other professionals~~. Although according to the respondents, people of all ages consult Italian osteopaths, the majority of patients are adults. Most of them have been referred to osteopathy by other patients or acquaintances. Patients seek osteopathic care mostly for musculoskeletal related complaints. The findings of the present study provide valuable insights into the osteopathic profession in Italy, which might be taken into consideration during the regulation process about the professional profile of competencies of the osteopathic profession in Italy. Follow-up studies have been planned to track future changes within the osteopathic profession.

Acknowledgements

Formatted: Font:

Formatted: Heading 1

The authors sincerely thank Prof. Angelo Manfredi and Prof. Fabrizio Consorti for their help in reviewing the paper. The OPERA-IT group is composed by: Marcello Luca Marasco (ABEos), Alberto Maggiani (AIMO), Antonio Cavallaro (AISERCO), Dario Silvestri (ASOMI), Joseph Zurlo and Marco Petracca (CERDO), Mauro Fornari (CIO), Alessandro Rapisarda (CSDOI), Giacomo Lo Voi (CSOT), Liria Papa (ICOM), Sandro Tamagnini (ICOMM), Roberta Filipazzi (IEMO), Tatiana Stirpe (Meta Osteopatia), Saverio Colonna (OSCE), Alessandro Gavazzi (SOFI), Andrea Manzotti and Andrea Bergna (SOMA), Sbarbaro Marco (SSOI), Federico Franscini (APO), Guglielmo Donnaquio (Osteopatia per Bambini), Alessandro Parisi (SIOS), Massimo Valente (Tuttosteopatia), Emanuele Botti (Advanced Osteopathy), osteopatiriconosciuti.

Author Contributions

Conceptualization: Francesco Cerritelli, Patrick L. S. van Dun, Jorge E. Esteves, Paola Sciomachen, Eleonora Lacorte, Nicola Vanacore.

Data curation: Francesco Cerritelli, Giacomo Consorti.

Formal analysis: Francesco Cerritelli, Jorge E. Esteves, Giacomo Consorti.

Funding acquisition: Francesco Cerritelli.

Investigation: Francesco Cerritelli, Patrick L. S. van Dun, Jorge E. Esteves.

Methodology: Francesco Cerritelli, Patrick L. S. van Dun, Jorge E. Esteves, Giacomo Consorti, Paola Sciomachen, Eleonora Lacorte, Nicola Vanacore.

Project administration: Francesco Cerritelli, Patrick L. S. van Dun, Jorge E. Esteves, Eleonora Lacorte.

Resources: Francesco Cerritelli, Massimo Valente

Software: Francesco Cerritelli, Massimo Valente

Supervision: Francesco Cerritelli, Patrick L. S. van Dun, Jorge E. Esteves, Paola Sciomachen, Eleonora Lacorte, Nicola Vanacore, Massimo Valente

Validation: Francesco Cerritelli, Patrick L. S. van Dun, Jorge E. Esteves, Nicola Vanacore.

Visualization: Francesco Cerritelli, Jorge E. Esteves, Giacomo Consorti.

Writing – original draft: Francesco Cerritelli, Patrick L. S. van Dun, Jorge E. Esteves, Giacomo Consorti.

Writing – review & editing: Francesco Cerritelli, Patrick L. S. van Dun, Jorge E. Esteves, Giacomo Consorti, Eleonora Lacorte, Nicola Vanacore.

Formatted: Font: Italian (Italy)
Formatted: Heading 1

References

1. Eumetra Monterosa. L’opinione degli italiani sull’osteopatia. 2017.

Formatted: Font: Italian (Italy)
Formatted: Heading 1

2. Cerritelli F, van Dun PLS, Esteves JE, Consorti G, Sciomachen P, Lacorte E, Vanacore N; OPERA-IT Group. The Italian Osteopathic Practitioners Estimates and RAtes (OPERA) study: A cross sectional survey. PLoS One. 2019 Jan 25;14(1):e0211353.

~~Cerritelli F, van Dun PLS, Esteves JE, Consorti G, Sciomachen P, Lacorte E, et al. The Italian Osteopathic Practitioners Estimates and RAtes (OPERA) study: A cross sectional survey. PloS one [Internet]. 2019 [cited 2019 Dec 20];14(1):e0211353. Available from: http://www.ncbi.nlm.nih.gov/pubmed/30682169~~

3. GOsC. Results – snapshot survey 2001. London; 2001.

4. KPMG. Report A: how do osteopaths practice? Zurich; 2011.

5. Burke SR, Myers R, Zhang AL. A profile of osteopathic practice in Australia 2010-2011: a cross sectional survey. BMC Musculoskelet Disord. 2013 Aug 1;14:227.

~~Burke SR, Myers R, Zhang AL. A profile of osteopathic practice in Australia 2010-2011: a cross sectional survey. BMC musculoskeletal disorders [Internet]. 2013 Aug 1 [cited 2019 Dec 20];14:227. Available from: http://www.ncbi.nlm.nih.gov/pubmed/23915239~~

6. Van Dun, P. L., Nicolaie, M. A., & Van Messem, A. State of affairs of osteopathy in the Benelux: Benelux Osteosurvey 2013. International Journal of Osteopathic Medicine, 2016; 20, 3-17.~~Dun P van, Nicolaie M, of AVM-IJ, 2016 undefined. State of affairs of osteopathy in the Benelux: Benelux Osteosurvey 2013. Elsevier [Internet]. [cited 2019 Dec 20]; Available from: https://www.sciencedirect.com/science/article/pii/S1746068916000043~~

7. Ciulla, L., Foucart, J., Lepers, Y., Cullus, P., & Milabi, A. K. Enquête d'opinions sur la perception de l'ostéopathie au sein de la population belge francophone. Mémoire présenté en vue de l'obtention du grade de Master en Sciences de la motricité, à finalité Ostéopathie, ULB, Belgique. 2014~~CIULLA L, FOUCART J, LEPERS Y, CULLUS P. Enquête d'opinions sur la perception de l'ostéopathie au sein de la population belge francophone.~~

~~mainslibres.ch [Internet]. [cited 2019 Dec 20]; Available from: http://www.mainslibres.ch/pdf/sommaire2016Resumes/sommaire_3_2016/resume_ciullia.pdf~~

8. Alvarez Bustins G, López Plaza PV, Carvajal SR. Profile of osteopathic practice in Spain: results from a standardized data collection study. BMC Complement Altern Med. 2018 Apr 11;18(1):129.

~~Alvarez Bustins G, López Plaza P-V, Carvajal SR. Profile of osteopathic practice in Spain: results from a standardized data collection study. BMC complementary and alternative medicine [Internet]. 2018 Apr 11 [cited 2019 Dec 20];18(1):129. Available from: http://www.ncbi.nlm.nih.gov/pubmed/29642901~~

9. Vaucher P, Macdonald RJD, Carnes D. The role of osteopathy in the Swiss primary health care system: a practice review. BMJ Open. 2018 Sep 1;8(8):e023770.

~~Vaucher P, Macdonald RJD, Carnes D. The role of osteopathy in the Swiss primary health care system: A practice review. Vol. 8, BMJ Open. BMJ Publishing Group; 2018.~~

10. Carreiro J FC. Foundations of Osteopathic Medicine. Third. Chila A editor, editor. Philadelphia: Wolters Kluwer/Lippincott Williams & Wilkins; 2011. 46–52.

11. Forum for Osteopathic Regulation in Europe (FORE). Regulation. 2018.

12. Senate of Republic of Italy. Law 3/2018. Rome: Senate of Republic of Italy ; 2018.

13. Sciomachen, P., Arienti, C., Bergna, A., Castagna, C., Consorti, G., Lotti, A., ... & Longobardi, M. Core competencies in osteopathy: Italian register of osteopaths proposal. International Journal of Osteopathic Medicine, 2018, 27, 1-5.~~Sciomachen P, … CA-I, 2018 undefined. Core competencies in osteopathy: Italian register of osteopaths proposal. journalofosteopathicmedicine.com [Internet]. [cited 2019 Dec 20]; Available from:~~ https://www.journalofosteopathicmedicine.com/article/S1746-0689(18)30035-X/abstract

14. Johnson SM, Kurtz ME. Conditions and diagnoses for which osteopathic primary care physicians and specialists use osteopathic manipulative treatment. Journal of the American Osteopathic Association. 2002;102(10):527–40.

15. Sibbritt D, Adams J, Young AF. A profile of middle-aged women who consult a chiropractor or osteopath: findings from a survey of 11,143 Australian women. J Manipulative Physiol Ther. 2006 Jun;29(5):349-53. ~~Sibbritt D, Adams J, Young AF. A profile of middle-aged women who consult a chiropractor or osteopath: findings from a survey of 11,143 Australian women. Journal of manipulative and physiological therapeutics [Internet]. 2006 Jun [cited 2019 Dec 20];29(5):349–53. Available from: http://www.ncbi.nlm.nih.gov/pubmed/16762661~~

16. Snider KT, Snider EJ, DeGooyer BR, Bukowski AM, Fleming RK, Johnson JC. Retrospective medical record review of an osteopathic manipulative medicine hospital consultation service. J Am Osteopath Assoc. 2013 Oct;113(10):754-67.~~Snider KT, Snider EJ, DeGooyer BR, Bukowski AM, Fleming RK, Johnson JC. Retrospective medical record review of an osteopathic manipulative medicine hospital consultation service. The Journal of the American Osteopathic Association [Internet]. 2013 Oct [cited 2019 Dec 20];113(10):754–67. Available from: http://www.ncbi.nlm.nih.gov/pubmed/24084802~~

17. Fawkes CA, Leach CMJ, Mathias S, Moore AP. A profile of osteopathic care in private practices in the United Kingdom: A national pilot using standardised data collection. Manual Therapy. 2014;19(2):125–30.

18. Morin C, Aubin A. Primary reasons for osteopathic consultation: a prospective survey in Quebec. PLoS One. 2014 Sep 3;9(9):e106259. Erratum in: PLoS One. 2015;10(3):e0121180.

~~Morin C, Aubin A. Primary reasons for osteopathic consultation: A prospective survey in Quebec. Vol. 9, PLoS ONE. Public Library of Science; 2014.~~

19. Channell MK, Wang Y, McLaughlin MH, Ciesielski J, Pomerantz SC. Osteopathic Manipulative Treatment for Older Patients: A National Survey of Osteopathic Physicians. J Am Osteopath Assoc. 2016 Mar;116(3):136-43.

Channell MK, Wang Y, McLaughlin MH, Ciesielski J, Pomerantz SC. Osteopathic Manipulative Treatment for Older Patients: A National Survey of Osteopathic Physicians. The Journal of the American Osteopathic Association [Internet]. 2016 Mar [cited 2019 Dec 20];116(3):136–43. Available from: http://www.ncbi.nlm.nih.gov/pubmed/26927907

20. dams J, Sibbritt D, Steel A, Peng W. A workforce survey of Australian osteopathy: analysis of a nationally-representative sample of osteopaths from the Osteopathy Research and Innovation Network (ORION) project. BMC Health Serv Res. 2018 May 10;18(1):352.

Adams J, Sibbritt D, Steel A, Peng W. A workforce survey of Australian osteopathy: analysis of a nationally-representative sample of osteopaths from the Osteopathy Research and Innovation Network (ORION) project. BMC health services research [Internet]. 2018 [cited 2019 Dec 20];18(1):352. Available from: http://www.ncbi.nlm.nih.gov/pubmed/29747647

21. Rosen MA, DiazGranados D, Dietz AS, Benishek LE, Thompson D, Pronovost PJ, Weaver SJ. Teamwork in healthcare: Key discoveries enabling safer, high-quality care. Am Psychol. 2018 May-Jun;73(4):433-450.

Rosen, M. A., DiazGranados, D., Dietz, A. S., Benishek, L. E., Thompson, D., Pronovost, P. J., & Weaver, S. J.. Teamwork in healthcare: Key discoveries enabling safer, high-quality care. American Psychologist, 73(4), 433.Rosen MA, DiazGranados D, Dietz AS, Benishek LE, Thompson D, Pronovost PJ, et al. Teamwork in healthcare: Key discoveries enabling safer, high-quality care. The American psychologist [Internet]. [cited 2019 Dec 20];73(4):433–50. Available from: http://www.ncbi.nlm.nih.gov/pubmed/29792459

22. Hammick M, Olckers L, Campion-Smith C. Learning in interprofessional teams: AMEE Guide no 38. Med Teach. 2009 Jan;31(1):1-12.

23. Hean S, Craddock D, Hammick M, Hammick M. Theoretical insights into interprofessional education: AMEE Guide No. 62. Med Teach. 2012;34(2):e78-101.

24. Bennett C, Khangura S, Brehaut JC, Graham ID, Moher D, Potter BK, Grimshaw JM. Reporting guidelines for survey research: an analysis of published guidance and reporting practices. PLoS Med. 2010 Aug;8(8):e1001069.

~~Bennett C, Khangura S, Brehaut JC, Graham ID, Moher D, Potter BK, et al. Reporting guidelines for survey research: An analysis of published guidance and reporting practices. Vol. 8, PLoS Medicine. 2011.~~

25~~3~~. Giusti R. Glossary of Osteopathic Terminology. Third. American association of colleges of osteopathic medicine, editor. American association of colleges of osteopathic medicine; 2017. 15.

26~~4~~. Institute of Medicine (US). Educating for the Health Team: Report of the Conference on the Interrelationships of Educational Programs for Health Professionals, 1972 Oct 2-3. National Academies. ~~(US) I of M. Educating for the Health Team: Report of the Conference on the Interrelationships of Educational Programs for Health Professionals, October 2-3, 1972. 1972 [cited 2019 Dec 20]; Available from: https://books.google.com/books?hl=it&lr=&id=8GArAAAAYAAJ&oi=fnd&pg=PP10&dq=%E2%97%8F%09Institute+of+Medicine.+(1972).+Educating+for+the+health+team.+Washington,+DC:+National+Academy+of+Sciences&ots=BXtJVenum7&sig=dtHtWQmQE6CFmWDOg5ri8J4iF1Q~~

27~~5~~. Institute of Medicine Committee on the Health Professions Education. The core competencies needed for health care professionals. In: A. C. Greiner & E. Knebel (Eds.), editor. Health professions education: A bridge to quality. Washington, DC: Washington, DC: National Academies Press.; 2003. p. 45–73.

28~~6~~. World Health Organization. Framework for action on interprofessional education and collaborative practice. Geneva, Switzerland; 2010.

29~~7~~. Berwick DM, Nolan TW, Whittington J. The triple aim: Care, health, and cost. Vol. 27, Health Affairs. 2008. p. 759–69.

30~~28~~. Schmitt M, Blue A, Aschenbrener CA, Viggiano TR. Core competencies for interprofessional collaborative practice: reforming health care by transforming health professionals' education. Acad Med. 2011 Nov;86(11):1351. ~~Interprofessional Education Collaborative (2011). Core Competencies for Interprofessional Collaborative Practice [Internet]. 2011 [cited 2019 Nov 10]. Available from: https://www.aacom.org/docs/default-source/insideome/ccrpt05-10-11.pdf?sfvrsn=77937f97_2~~

31~~29~~. Nandan M, Scott P. An innovative interprofessional education model to engage community and nonclinical participants. J Interprof Care. 2014 Jul;28(4):376-8.

~~Nandan M, Scott P. An innovative interprofessional education model to engage community and nonclinical participants. Journal of interprofessional care [Internet]. 2014 Jul [cited 2019 Dec 20];28(4):376–8. Available from: http://www.ncbi.nlm.nih.gov/pubmed/24617846~~

32~~30~~. Miccolo MA, Spanier AH. Critical care management in the 1990s. Making collaborative practice work. Crit Care Clin. 1993 Jul;9(3):443-53.

~~Miccolo MA, Spanier AH. Critical care management in the 1990s. Making collaborative practice work. Critical care clinics [Internet]. 1993 Jul [cited 2019 Dec 20];9(3):443–53. Available from: http://www.ncbi.nlm.nih.gov/pubmed/8353783~~

33~~1~~. Hojat M, Fields SK, Veloski JJ, Griffiths M, Cohen MJM, Plumb JD. Psychometric properties of an attitude scale measuring physician-nurse collaboration. Evaluation and the Health Professions. 1999 Jun;22(2):208–20.

34~~2~~. Whitehead D. Applying collaborative practice to health promotion. Nurs Stand. 2001 Jan 31-Feb 6;15(20):33-7.

Whitehead D. Applying collaborative practice to health promotion. Vol. 15, Nursing standard (Royal College of Nursing (Great Britain) : 1987). 2001. p. 33–7.

353. King O, Nancarrow SA, Borthwick AM, Grace S. Contested professional role boundaries in health care: A systematic review of the literature. Journal of Foot and Ankle Research. 2015 Feb 5;8(1).

Formatted: Font: (Default) Times New Roman, 12 pt, Bold

Formatted: Font:

Review of the manuscript

Manuscript number PONE-D-19-35428, entitled "The Italian Osteopathic Practitioners Estimates and RAtes (OPERA) study: how osteopaths work"

Dear editor,
Dear reviewers,

We greatly appreciate your readiness to have read our paper and to provide us with relevant feedback and useful suggestions to further improve the quality of our paper. A detailed description of all changes has been provided below.

For any further information, please do not hesitate to contact us.

Editor's comments

Journal Requirements:

When submitting your revision, we need you to address these additional requirements:

1. Please ensure that your manuscript meets PLOS ONE's style requirements, including those for file naming. The PLOS ONE style templates can be found at http://www.plosone.org/attachments/PLOSOne_formatting_sample_main_body.pdf and http://www.plosone.org/attachments/PLOSOne_formatting_sample_title_authors_affiliations.pdf

Response: Thank you, done

2. We note that you have indicated that data from this study are available upon request. PLOS only allows data to be available upon request if there are legal or ethical restrictions on sharing data publicly. For information on unacceptable data access restrictions, please see http://journals.plos.org/plosone/s/data-availability#loc-unacceptable-data-access-restrictions.

In your revised cover letter, please address the following prompts:

a) If there are ethical or legal restrictions on sharing a de-identified data set, please explain them in detail (e.g., data contain potentially identifying or sensitive patient information) and who has imposed them (e.g., an ethics committee). Please also provide contact information for a data access committee, ethics committee, or other institutional body to which data requests may be sent.

b) If there are no restrictions, please upload the minimal anonymized data set necessary to replicate your study findings as either Supporting Information files or to a stable, public repository and provide us with the relevant URLs, DOIs, or accession numbers. Please see http://www.bmj.com/content/340/bmj.c181.long for guidelines on how to de-identify and prepare clinical data for publication. For a list of acceptable repositories, please see http://journals.plos.org/plosone/s/data-availability#loc-recommended-repositories.

Response: There was an error during the submission process, all the relevant data are available in the manuscript. In any case please refer to the revised version of the cover letter.

3. One of the noted authors is a group or consortium OPERA-IT group. In addition to naming the author group, please list the individual authors and affiliations within this group in the acknowledgments section of your manuscript. Please also indicate clearly a lead author for this group along with a contact email address.

Response: The list of authors are presented in the Acknowledgments. The lead author is Francesco Cerritelli and was included as suggested

4. Please include captions for your Supporting Information files at the end of your manuscript, and update any in-text citations to match accordingly. Please see our Supporting Information guidelines for more information: http://journals.plos.org/plosone/s/supporting-information.

Response: All the relevant data are available in the manuscript.

Reviewer 2

Reviewer #2: Thank you for the opportunity to review this important research. I have some suggestions to strengthen the article.
1. In general, it is best to avoid starting a sentence with numeric characters (e.g. 4916 or 90%). Instead, you would need to restructure the sentence so it does not start with a numeric value or write the number in words (e.g. ninety percent).

Response: Thank you for your suggestion. The manuscript has been changed accordingly.

2. Line 71: What is osteopathy "growing' from? What is the evidence it is "growing"? Are you referring to an increase in number of practitioners? Or consultations? or something else?

Response: Thank you for your suggestion. We rephrased the sentence to make it less prone to interpretation as follow: "*Osteopathy is a widespread health profession in Italy*".

3. Lines 152-158: the recruitment process needs to be clarified. What are the 12 steps? What 'other sources of information' are you referring to? Other contacts for osteopaths?

Response: Thank you for your comments. We added an example of what we meant with "different sources "*(e.g. promotional databases for healthcare professionals)*" and we rephrased the promotional strategy sentence as follow: *"The promotion strategy consisted of the dispatch of the e-flyer to all the different mailing lists".*

4. Results: this entire section needs English editing - particularly the first section "Comparison between osteopaths who work alone and associated". The term 'associated' is not self-evident and should probably be revised.

Response:
Thank you for the comment. The section has been reviewed and improved for clarity

5. There is no need to dedicate so much of the discussion to repeating the results. Further, the attempts to contextualise the discussion with external research is evident, but not entirely successful. Lines 331-338 are sentences without a paragraph. While Lines 341-365 appear to be dedicated to one body of work. Meanwhile, the significance of many of the key findings (as outlined in your conclusion) have not been positioned alongside existing relevant research.

Response: Thank you for your comment. Discussion have been changed accordingly.

Reviewer 3

Reviewer #3: Thank you for the opportunity to review this manuscript. Overall the premise of the work is interesting however there are some significant limitations with the statistical analysis and

the description of the results. Further, there is little discussion of the work in the Discussion section of the manuscript. I have made comments and suggestions throughout the attached version of the manuscript and I hope that the authors find these useful in revising the work.

The outcomes of the previous OPERA study should be described in the Introduction as they appear to be pertinent to the current study. There also needs to be greater consideration of other European studies and what they describe as the profile of osteopaths in those countries.

Response: Thanks for your advice. We provided more detailed information both on OPERA and on the other EU and international studies.

It would be valuable to describe who these participants are. Are they member of the general public?

Response: Thank you for your comment. The missing information has been added as follow “*In a recent national opinion survey conducted on a sample of 800 participants from the general public by Eumetra Monterosa* “

It is not clear here as to the purpose of this sentence. It makes reference to a previous study by describes the current work as the "present study". It may be better to remove this sentence however.

Response: Thank you for your comment, the term “present study” has been replaced by “*OPERA study”*

Regulation is also in New Zealand and Australia.

Response: Thank you for your comment, we listed just the European countries since it gives a more accurate picture of the specific context.

Please clarify what is meant by "proper" in this context.

Response: Thank you for your comment, the term “proper” has been replaced by “*official”*

What do these studies generally suggest are the main reasons for consultation with an osteopath? Other common characteristics across jurisdictions?

Response: Thank you for your comment, a brief report of the primary reasons for osteopathic consultation reported in those studies has been added.

Please provide some examples of the type of health professional they work with

Response: Thank you for your comment, an example has been added.

Additional references here would also be useful. One reference for a fundamental aspect of healthcare is likely insufficient.

Response: Thank you for your comment, more references supporting the concept have been added.

Please clarify is this in relation to practicing alone or with others.

Response: Thank you for your comment, the sentence has been removed because it was not pertinent.

Please provide additional detail here about the recrutiment of participants to the OPERA-IT study population. How was it determined that this was a representative sample?
It would also be valuable to clarify if the recruitment is different to the 2019 OPERA study. At present, the manuscript reads as though there is a different recruitment strategy for the current work.

Response: Thank you for your comment. We clarified that the data were collected from the same database used in the previous study. So the data collection was only 1 for both studies. Furthermore, we specified that “*the theoretical representativeness*” were addressed through the eligibility criteria.

It would appear that this is the entire OPERA-IT sample? Please clarify how these would be inclusion criteria for the current work.

Response: Thank for your comment. As per the comment above we clarified that the database was the same.

Were these people eligible to be in the OPERA-IT database?

Response: Thank for your question. Those criteria are the very same of the OPERA-IT study. We added few examples to clarify the statement.

Please ensure that the terminology is consistent throughout. Osteopath, osteopathic practitioner, osteopathic professional.

Response: Thanks for your comment. done

Please clarify what this abbreviation refers to.

Response: Thanks for your comment. done

Please provide the dates for this here.

Response: Thanks for your comment. done

Not necessarily "validated" but has been used and reported on previously. This does not constitute validation.

Response: Thanks for your comment. We rephrased accordingly

Assuming this is the World Health Organisation?

Response: Good guess! We added an explanation of the abbreviation.

Please provide a rationale for the use of relative risk over an odds ratio - the latter being more common in study designs such as the current one, particularly if logistic regression is used. RRs are not able to be used in logistic regression.

Response: Thank you for this comment. Erroneously the relative risk was included in the methods section but then in the results the odds ratio was used as suggested. Thus, we corrected the methods accordingly.

The purpose of the PCA in relation to the study is not entirely clear here. What was the purpose of identifying the components that comprised the questionnaire given that a number of variables are reported here? How was a score created for each component to be entered into the regression model?

Response: The following sentence was added in the methods section **"**PCA was used as a method to reduce the number of variables by extracting important elements from the large pool of variables we collected. This process aims to retain as much information as possible bringing out strong patterns in a dataset. The patterns were, then, identified in the three major areas based on similarities of variables." Concerning the score, the explanation was detailed in the section PCA and logistic regression.

It would be valuable to provide a rationale for the use of the components in the logistic regression versus the individual items on the questionnaire. The process of the logistic regression also need to be described so readers can understand how the model was built.

Response: A detailed description was added and summarised as follows: by transforming a large set of variables into a smaller one that still contains most of the information of the large set, we could include, indeed, the majority of the variables into the logistic regression. On the contrary, if we did not use this approach, this process could not have been taken as the excessive number of variables would not be statistically appropriate to be included in the analysis.
The logistic model was also included

Please clarify the purpose of these groupings given that a PCA is to be performed.

Response: Thank you, PCA and logistic regression section was improved accordingly.

This would just be missing data rather than attrition.

Response: Well, actually the 196 questionnaires that were incomplete, that is participants started but then not finished, can be referred to as attrition, or better respondent attrition.

They also appear to be reported in Table 2?

Response: Thank you for your comment. The sentence has been deleted

It would be useful to ensure that the terminology is consistent throughout. Either 'collaborations' or 'associated'

Response: Thanks for your comment. done

Given this, a reader may ask about the value of the PCA. The components being used in the logistic regression may lose the nuance in the data.

Response: Thank you for the comment. Please refer to the previous amendments. Hopefully we improved the methods section in order to clarify better this point

Relative risk was described in the statistical analysis section however ORs are reported here. Please clarify.

Response: Thank you. Correction made

This doesn't appear to be a complete sentence.

Response: Thanks for your comment. The sentence has been rephrased

Why was 'north-west' chosen as the exposure variable?

Response: It was arbitrarily chosen but based on the rationale that the north-west region was the most representative in terms of number of osteopaths

It may not be necessary to report the ORs that are not significant and where the CI crosses 1

Response: Thank you for the comment. However, it might be useful to have a full spectrum of the data as they might be useful for further studies. Indeed, it is true that we need to refer to the statistically significant values, but the direction of effect might be a useful element to report.

Please clarify the meaning of T1 and T2 here as most readers will not understand this.

Response: Thanks for your comment. done

Assuming this should be 6?

Response: Thanks for your comment. Well...yes. My apologies.

How do these relate to the working relationship with other health professionals? If this is background for the reader, it may be better placed either in the beginning of the results.

Response: Thank you for your comment. The paragraph has been moved at the beginning of results.

Please clarify the basis on which the sample is considered to be nationally representative.

Response: Thank for your comment. We added an explanatory sentence in the method to clarify why we address the sample as "representative".
"*The sample size was arbitrarily estimated and measured summing all practitioners owning a Diploma in Osteopathy or equivalent released from an Italian or an international osteopathic educational institution up to December 2016. That provided an estimated 5,100 osteopaths sample. Considering a standard deviation of 10%, the number of osteopaths in Italy was expected to range from 4,600 to 5,600. Assuming a response rate between 10 and 60 percent of those receiving the questionnaire the number of osteopaths taking part in the survey was estimated to be between 460 and 3,300.*"

Please clarify this part of the sentence. Is it referring to geographical distribution?

Response: Thank you for your comment. Done.

This aspect of the paragraph is likely not required as it is already part of the Methods.

Response: Thank you for your comment. The sentence has been deleted.

it would be useful to include the reference to the original study here.

Response: Thank you for your comment. Done.

Not sure if 'might' is the best word here. The work certainly contributes to the understanding of Italian osteopathic practice.

Response: Thank you for your comment. Changed accordingly.

These sentences could be removed as the essentially restating what is already in the Introduction and Method

Response: Thank you for your comment. Deleted.

Please clarify if the exposure variable is 'alone'? If so, then these osteopaths are 8% more likely. It would be difficult to categorically state they are not delivering these aspects of practice.

Response: The exposure variable is type of practice (sole practitioner vs group of practice), thus the discussion focuses on the comparison between the two groups. Therefore, the 8% is relative to the group of practice as compared to the alone [which was considered the reference category]. Then it is more likely that they are using those aspects but it does not imply they do not use them.

As per the comment above about the exposure variable, the descriptions should be in relation to the exposure variable.

Response: Please see the comment above

These are all reasonable statements but they need to be described in the context of the current work.

Response: Thank you for your comment. Discussion has been changed accordingly.

As above, these paragraphs need to be described in the context of the findings of the study.

Response: Thank you for your comment. Discussion have been changed accordingly.

This should be related to working alone or with

Response: Thank you for your comment. The reported data refers to the whole sample.

Which findings of the current study are relevant here?

Response: Thank you for your comment. We specified.

These are reasonable comments however it is not clear how they relate to the current study.

Response: Thank you for your comment. Discussion have been changed accordingly.

We hope that our answers and the revision of our manuscript is meeting your expectations. We want to thank the reviewers again for providing us with the feedback and useful suggestions.

Sincerely,

The authors
