# Supplementary material for: The Italian Osteopathic Practitioners Estimates and RAtes (OPERA) study: How osteopaths work
Source: PLoS One. 2020 Jul 2;15(7):e0235539. doi: 10.1371/journal.pone.0235539 (PMC7332305; doi:10.1371/journal.pone.0235539)
Supplement: Attachment — Submitted filename: Response to reviewers.docx [file pone.0235539.s003.docx]

**Review of the manuscript**

**Manuscript number PONE-D-19-35428, entitled “The Italian Osteopathic Practitioners Estimates and RAtes (OPERA) study: how osteopaths work”**

Dear editor,

Dear reviewers,

We greatly appreciate your readiness to have read our paper and to provide us with relevant feedback and useful suggestions to further improve the quality of our paper. A detailed description of all changes has been provided below.

For any further information, please do not hesitate to contact us.

**Editor’s comments**

Journal Requirements:

When submitting your revision, we need you to address these additional requirements:

1. Please ensure that your manuscript meets PLOS ONE's style requirements, including those for file naming. The PLOS ONE style templates can be found at <http://www.plosone.org/attachments/PLOSOne_formatting_sample_main_body.pdf> and <http://www.plosone.org/attachments/PLOSOne_formatting_sample_title_authors_affiliations.pdf>

**Response:** Thank you, done

2. We note that you have indicated that data from this study are available upon request. PLOS only allows data to be available upon request if there are legal or ethical restrictions on sharing data publicly. For information on unacceptable data access restrictions, please see <http://journals.plos.org/plosone/s/data-availability#loc-unacceptable-data-access-restrictions>.

In your revised cover letter, please address the following prompts:

a) If there are ethical or legal restrictions on sharing a de-identified data set, please explain them in detail (e.g., data contain potentially identifying or sensitive patient information) and who has imposed them (e.g., an ethics committee). Please also provide contact information for a data access committee, ethics committee, or other institutional body to which data requests may be sent.

b) If there are no restrictions, please upload the minimal anonymized data set necessary to replicate your study findings as either Supporting Information files or to a stable, public repository and provide us with the relevant URLs, DOIs, or accession numbers. Please see <http://www.bmj.com/content/340/bmj.c181.long> for guidelines on how to de-identify and prepare clinical data for publication. For a list of acceptable repositories, please see <http://journals.plos.org/plosone/s/data-availability#loc-recommended-repositories>.

**Response:** There was an error during the submission process, all the relevant data are available in the manuscript. In any case please refer to the revised version of the cover letter.

3. One of the noted authors is a group or consortium OPERA-IT group. In addition to naming the author group, please list the individual authors and affiliations within this group in the acknowledgments section of your manuscript. Please also indicate clearly a lead author for this group along with a contact email address.

**Response:** The list of authors are presented in the Acknowledgments. The lead author is Francesco Cerritelli and was included as suggested

4. Please include captions for your Supporting Information files at the end of your manuscript, and update any in-text citations to match accordingly. Please see our Supporting Information guidelines for more information: <http://journals.plos.org/plosone/s/supporting-information>.

**Response:** All the relevant data are available in the manuscript.

**Reviewer 2**

Reviewer #2: Thank you for the opportunity to review this important research. I have some suggestions to strengthen the article.
1. In general, it is best to avoid starting a sentence with numeric characters (e.g. 4916 or 90%). Instead, you would need to restructure the sentence so it does not start with a numeric value or write the number in words (e.g. ninety percent).

**Response:** Thank you for your suggestion. The manuscript has been changed accordingly.

2. Line 71: What is osteopathy "growing' from? What is the evidence it is "growing"? Are you referring to an increase in number of practitioners? Or consultations? or something else?

**Response:** Thank you for your suggestion. We rephrased the sentence to make it less prone to interpretation as follow: “*Osteopathy is a widespread health profession in Italy*”.

3. Lines 152-158: the recruitment process needs to be clarified. What are the 12 steps? What 'other sources of information' are you referring to? Other contacts for osteopaths?

**Response:** Thank you for your comments. We added an example of what we meant with “different sources ”*(e.g. promotional databases for healthcare professionals)*” and we rephrased the promotional strategy sentence as follow: *“The promotion strategy consisted of the dispatch of the e-flyer to all the different mailing lists”.*

4. Results: this entire section needs English editing - particularly the first section "Comparison between osteopaths who work alone and associated". The term 'associated' is not self-evident and should probably be revised.

**Response:**

Thank you for the comment. The section has been reviewed and improved for clarity

5. There is no need to dedicate so much of the discussion to repeating the results. Further, the attempts to contextualise the discussion with external research is evident, but not entirely successful. Lines 331-338 are sentences without a paragraph. While Lines 341-365 appear to be dedicated to one body of work. Meanwhile, the significance of many of the key findings (as outlined in your conclusion) have not been positioned alongside existing relevant research.

**Response:** Thank you for your comment. Discussion have been changed accordingly.

**Reviewer 3**

Reviewer #3: Thank you for the opportunity to review this manuscript. Overall the premise of the work is interesting however there are some significant limitations with the statistical analysis and the description of the results. Further, there is little discussion of the work in the Discussion section of the manuscript. I have made comments and suggestions throughout the attached version of the manuscript and I hope that the authors find these useful in revising the work.

The outcomes of the previous OPERA study should be described in the Introduction as they appear to be pertinent to the current study. There also needs to be greater consideration of other European studies and what they describe as the profile of osteopaths in those countries.

**Response:** Thanks for your advice. We provided more detailed information both on OPERA and on the other EU and international studies.

It would be valuable to describe who these participants are. Are they member of the general public?

**Response:** Thank you for your comment. The missing information has been added as follow “*In a recent national opinion survey conducted on a sample of 800 participants from the general public by Eumetra Monterosa* “

It is not clear here as to the purpose of this sentence. It makes reference to a previous study by describes the current work as the "present study". It may be better to remove this sentence however.

**Response:** Thank you for your comment, the term “present study” has been replaced by “*OPERA study”*

Regulation is also in New Zealand and Australia.

**Response:** Thank you for your comment, we listed just the European countries since it gives a more accurate picture of the specific context.

Please clarify what is meant by "proper" in this context.

**Response:** Thank you for your comment, the term “proper” has been replaced by “*official”*

What do these studies generally suggest are the main reasons for consultation with an osteopath? Other common characteristics across jurisdictions?

**Response:** Thank you for your comment, a brief report of the primary reasons for osteopathic consultation reported in those studies has been added.

Please provide some examples of the type of health professional they work with

**Response:** Thank you for your comment, an example has been added.

Additional references here would also be useful. One reference for a fundamental aspect of healthcare is likely insufficient.

**Response:** Thank you for your comment, more references supporting the concept have been added.

Please clarify is this in relation to practicing alone or with others.

**Response:** Thank you for your comment, the sentence has been removed because it was not pertinent.

Please provide additional detail here about the recrutiment of participants to the OPERA-IT study population. How was it determined that this was a representative sample?

It would also be valuable to clarify if the recruitment is different to the 2019 OPERA study. At present, the manuscript reads as though there is a different recruitment strategy for the current work.

**Response:** Thank you for your comment. We clarified that the data were collected from the same database used in the previous study. So the data collection was only 1 for both studies. Furthermore, we specified that “*the theoretical representativeness*” were addressed through the eligibility criteria.

It would appear that this is the entire OPERA-IT sample? Please clarify how these would be inclusion criteria for the current work.

**Response:** Thank for your comment. As per the comment above we clarified that the database was the same.

Were these people eligible to be in the OPERA-IT database?

**Response:** Thank for your question. Those criteria are the very same of the OPERA-IT study. We added few examples to clarify the statement.

Please ensure that the terminology is consistent throughout. Osteopath, osteopathic practitioner, osteopathic professional.

**Response:** Thanks for your comment. done

Please clarify what this abbreviation refers to.

**Response:** Thanks for your comment. done

Please provide the dates for this here.

**Response:** Thanks for your comment. done

Not necessarily "validated" but has been used and reported on previously. This does not constitute validation.

**Response:** Thanks for your comment. We rephrased accordingly

Assuming this is the World Health Organisation?

**Response:** Good guess! We added an explanation of the abbreviation.

Please provide a rationale for the use of relative risk over an odds ratio - the latter being more common in study designs such as the current one, particularly if logistic regression is used. RRs are not able to be used in logistic regression.

**Response:** Thank you for this comment. Erroneously the relative risk was included in the methods section but then in the results the odds ratio was used as suggested. Thus, we corrected the methods accordingly.

The purpose of the PCA in relation to the study is not entirely clear here. What was the purpose of identifying the components that comprised the questionnaire given that a number of variables are reported here? How was a score created for each component to be entered into the regression model?

**Response:** The following sentence was added in the methods section **“**PCA was used as a method to reduce the number of variables by extracting important elements from the large pool of variables we collected. This process aims to retain as much information as possible bringing out strong patterns in a dataset. The patterns were, then, identified in the three major areas based on similarities of variables.” Concerning the score, the explanation was detailed in the section PCA and logistic regression.

It would be valuable to provide a rationale for the use of the components in the logistic regression versus the individual items on the questionnaire. The process of the logistic regression also need to be described so readers can understand how the model was built.

**Response:** A detailed description was added and summarised as follows: by transforming a large set of variables into a smaller one that still contains most of the information of the large set, we could include, indeed, the majority of the variables into the logistic regression. On the contrary, if we did not use this approach, this process could not have been taken as the excessive number of variables would not be statistically appropriate to be included in the analysis.

The logistic model was also included

Please clarify the purpose of these groupings given that a PCA is to be performed.

**Response:** Thank you, PCA and logistic regression section was improved accordingly.

This would just be missing data rather than attrition.

**Response:** Well, actually the 196 questionnaires that were incomplete, that is participants started but then not finished, can be referred to as attrition, or better respondent attrition.

They also appear to be reported in Table 2?

**Response:** Thank you for your comment. The sentence has been deleted

It would be useful to ensure that the terminology is consistent throughout. Either 'collaborations' or 'associated'

**Response:** Thanks for your comment. done

Given this, a reader may ask about the value of the PCA. The components being used in the logistic regression may lose the nuance in the data.

**Response:** Thank you for the comment. Please refer to the previous amendments. Hopefully we improved the methods section in order to clarify better this point

Relative risk was described in the statistical analysis section however ORs are reported here. Please clarify.

**Response:** Thank you. Correction made

This doesn't appear to be a complete sentence.

**Response:** Thanks for your comment. The sentence has been rephrased

Why was 'north-west' chosen as the exposure variable?

**Response:** It was arbitrarily chosen but based on the rationale that the north-west region was the most representative in terms of number of osteopaths

It may not be necessary to report the ORs that are not significant and where the CI crosses 1

**Response:** Thank you for the comment. However, it might be useful to have a full spectrum of the data as they might be useful for further studies. Indeed, it is true that we need to refer to the statistically significant values, but the direction of effect might be a useful element to report.

Please clarify the meaning of T1 and T2 here as most readers will not understand this.

**Response:** Thanks for your comment. done

Assuming this should be 6?

**Response:** Thanks for your comment. Well...yes. My apologies.

How do these relate to the working relationship with other health professionals? If this is background for the reader, it may be better placed either in the beginning of the results.

**Response:** Thank you for your comment. The paragraph has been moved at the beginning of results.

Please clarify the basis on which the sample is considered to be nationally representative.

**Response:** Thank for your comment. We added an explanatory sentence in the method to clarify why we address the sample as “representative”.
“*The sample size was arbitrarily estimated and measured summing all practitioners owning a Diploma in Osteopathy or equivalent released from an Italian or an international osteopathic educational institution up to December 2016. That provided an estimated 5,100 osteopaths sample. Considering a standard deviation of 10%, the number of osteopaths in Italy was expected to range from 4,600 to 5,600. Assuming a response rate between 10 and 60 percent of those receiving the questionnaire the number of osteopaths taking part in the survey was estimated to be between 460 and 3,300.*”

Please clarify this part of the sentence. Is it referring to geographical distribution?

**Response:** Thank you for your comment. Done.

This aspect of the paragraph is likely not required as it is already part of the Methods.

**Response:** Thank you for your comment. The sentence has been deleted.

it would be useful to include the reference to the original study here.

**Response:** Thank you for your comment. Done.

Not sure if 'might' is the best word here. The work certainly contributes to the understanding of Italian osteopathic practice.

**Response:** Thank you for your comment. Changed accordingly.

These sentences could be removed as the essentially restating what is already in the Introduction and Method

**Response:** Thank you for your comment. Deleted.

Please clarify if the exposure variable is 'alone'? If so, then these osteopaths are 8% more likely. It would be difficult to categorically state they are not delivering these aspects of practice.

**Response:** The exposure variable is type of practice (sole practitioner vs group of practice), thus the discussion focuses on the comparison between the two groups. Therefore, the 8% is relative to the group of practice as compared to the alone [which was considered the reference category]. Then it is more likely that they are using those aspects but it does not imply they do not use them.

As per the comment above about the exposure variable, the descriptions should be in relation to the exposure variable.

**Response:** Please see the comment above

These are all reasonable statements but they need to be described in the context of the current work.

**Response:** Thank you for your comment. Discussion has been changed accordingly.

As above, these paragraphs need to be described in the context of the findings of the study.

**Response:** Thank you for your comment. Discussion have been changed accordingly.

This should be related to working alone or with

**Response:** Thank you for your comment. The reported data refers to the whole sample.

Which findings of the current study are relevant here?

**Response:** Thank you for your comment. We specified.

These are reasonable comments however it is not clear how they relate to the current study.

**Response:** Thank you for your comment. Discussion have been changed accordingly.

We hope that our answers and the revision of our manuscript is meeting your expectations. We want to thank the reviewers again for providing us with the feedback and useful suggestions.

Sincerely,

The authors
