# Supplementary material for: The Italian Osteopathic Practitioners Estimates and RAtes (OPERA) study: How osteopaths work
Source: PLoS One. 2020 Jul 2;15(7):e0235539. doi: 10.1371/journal.pone.0235539 (PMC7332305; doi:10.1371/journal.pone.0235539)
Supplement: Attachment — Submitted filename: Response to reviewers.docx [file pone.0235539.s005.docx]

**Review of the manuscript**

**Manuscript number PONE-D-19-35428, entitled “The Italian Osteopathic Practitioners Estimates and RAtes (OPERA) study: how osteopaths work”**

Dear editor,

Dear reviewers,

We greatly appreciate your readiness to have read our paper and to provide us with relevant feedback and useful suggestions to further improve the quality of our paper. A detailed description of all changes has been provided below.

For any further information, please do not hesitate to contact us.

**Reviewer 2**

Reviewer #2: Discussion:

P18 - the higher number of new osteopaths in team environments may also reflect an increased integration acceptability of the osteopathic profession in the Italian health system and openness from other health professionals to collaborate with them. The fact that this is more common among younger osteos may be because older osteos are already established in a clinical environment. The attitude of new graduates may still play a role, but the relationship may also be changing from the other direction as well.

**Response:** Thank you for this insight, we added your consideration to the discussion.

P18-19 - this is a very long paragraph. I feel you could reduce it down to make your point more succinctly. I also think the paragraph could end with a call for more research examining the structural factors that may impact on the efficiency of osteopaths' inclusion in team environments. Are they charging more when operating as a team because the clinical environments are in more costly locations with more infrastructure? (e.g. reception staff). What does this mean for equity and access of osteopathy?

**Response:** Thank you for this suggestion, we added your consideration to the discussion.

P19 - the smaller second paragraph here could be moved to the beginning of the discussion as I think this is an overall finding of the study. It gets lost where it is. The points about team vs solo practice are secondary to this, from my perspective.

**Response:** Thank you for your suggestion. Done.

**Reviewer 3**

Please clarify what is meant by 'widespread'? Is it that practitioners are geographically spread? Or that it is widely utilised by the population?

**Response:** Thank you for your comment. it has been rephrased as follow: “*Osteopathy is a widely used health profession in Italy.*”

This level of detail is not required. If it is to be included, then other profile studies should be described here also. AND This discussion is more relevant and means that the section from the Beneleux study can be removed above.

**Response:** Thank you for your comment, the upper section has been removed

Best to clarify if it is the actual survey tool being described here or the OPERA study overall.

**Response:** Thank you for your comment, we rephrased as follow “*Arguably, OPERA study is a relevant project for all the stakeholders interested in obtaining up-to-date…*”

There is significant overlap between the text and the table. The text should only list the key findings and reference made to the table for all other components.

**Response:** Thank you for the comment. As you correctly suggested, the text reported only the key findings. Indeed, the table reports many more data compared to the text.

It is not clear what is meant by these sentences. Please clarify.

**Response:** We tried to implement but the way in which is described appears to be in line with other publications using the same statistical methods.

This is not increased if the OR is 0.91. It is 8% less likely.

**Response:** Correct, but it is less likely for team practitioners as the reference category is sole. So it depends on how the data is read and we feel that the sentence seems to be correct

It would be useful for the reader to name these components.

**Response:** Again thank you for suggesting. To the best of our knowledge, the PCA produced a given number of components that are numbered numerically. Therefore, PC-3 appears to be comprehensible (also looking at table 3) and in line with the standard terminology.

As per the comment above.

**Response:** as per answer as above

It may also represent an emphasis in education programs on interprofessional care.

**Response:** Thank you for this suggestion, we added your consideration to the discussion.

How does this information relate to the results of the current study?

**Response:** Thank you for your question. That sentence is an opening statement to discuss in the following paragraphs the pros and cons of working in an interprofessional team which is particularly relevant to the present study since the majority of the sample declared to work in one and since it was the main criteria we used to compose the 2 groups.

is this referring to Italy or more broadly. Please provide a reference or two too support this statement.

**Response:** Thank you for your question. That sentence is supported by references 30 and 31 reported at the end of the paragraph. If needed we will report them as well at the end of the first sentence.

The link between this sentence and the previous one is not clear. Please clarify for the reader the relevance of collaborating with medical specialists in particular.

**Response:** Thank you for your comment, the sentence has been removed.

This sentence appears to be talking about a different finding completely. This is more related to cost rather than interprofessional care.

**Response:** Thank you for your comment. As you correctly pointed out this paragraph examines the costs and quality of service related to interprofessional care rather than interprofessional care as a whole, indeed, few lines upper it has been reported: “*it is required that both clinicians and non-clinician members of the healthcare team collaborate to optimize the cost/effectiveness of their intervention ^30,31^*”. In the sentence you highlighted we reported that our findings seem to be in contrast to that previous statement.

Again, it is not quite clear what is meant here. Is that that the cost effectiveness of interprofessional care where osteopaths are involved is required?

**Response:** Thank you for your comment. We deleted that sentence and we added a new one highlighting the need of research in both clinical effectiveness and cost/efficacy of integrating osteopathy into an interprofessional team. I report the new sentence: “*Future research focused on examining the structural factors that may impact on the efficiency of osteopaths' inclusion in team environments is needed. In particular, it can be beneficial to investigate the reasons for the difference in the cost related to the osteopathic services and the impact it might have on the equity and access of osteopathic care for the general population.”*

This detail is not required as it does not appear to be put in the context of the current findings.

**Response:** Thank you for your opinion. However, we believe that reporting what it’s known on the pros and cons of interprofessional practice is particularly relevant to the understanding of the differences between the two groups and it is a possible interpretative key of the reported findings.

So what might this mean? Is it that because of their training they are more likely to use OCF? Or that use of these techniques may be problematic in interprofessional care? Evidence-base for the techniques?

**Response:** Thanks for your questions. Our findings allow us only to state that the two groups appear to use different approaches. The fact that the approaches might differ from sole practitioners and team members is supported by the previous sentence “*Patients can see a more positive, focused and coordinated approach to their health needs and have more faith in it. Finally, there is a higher likelihood of a more intensive and holistic approach, which is particularly relevant to osteopathic practice.*” Every sort of answer we could try to give to your questions, unfortunately, would be completely speculative since we have no data to support any of the possible answers so we preferred avoiding being speculative and we reported the data contextualized within the pertinent literature.

Again, this detail is useful but needs to be contextualised. How does it relate to the current study?

**Response:** Thank you for your comment. We are not sure how defining the pros and cons of working as a team might not relate to the present study since we are highlighting the differences between osteopaths working interprofessional teams and those working as sole practitioners. As we reported in a previous answer we think that it is particularly relevant to the present study and it gives context and ground to the results.

How does this relate to the research question about sole versus team practice?

**Response:** Thank you for your comment, the part of the aim of the study stating “*Moreover, patients’ characteristics and primary reasons for consultation will be presented*” which was present in the first draft might have been lost in the editing. We added it back where it was. Thanks again for noticing, it was a big miss.

The focus in the Discussion is on medical specialists. Please clarify.

**Response:** Thank you for your comment, we deleted the sentence linking the discussion to the sole medical profession.

These weren't findings related to the research question. How do they relate to sole versus team practice?

**Response:**Thank you for your comment, the part of the aim of the study stating “*Moreover, patients’ characteristics and primary reasons for consultation will be presented*” which was present in the first draft might have been lost in the editing. We added it back where it was. Thanks again for noticing, it was a big miss.

The emphasis here should be on interprofessional care rather than an 'osteopathy-centric' discussion.

**Response:** Interprofessional practice is usually a facet of the professional profile (e.g. “collaborator” in the CanMed framework) so we highlighted the endpoint which leads us to the definition of the scope of practice.

We hope that our answers and the revision of our manuscript is meeting your expectations. We want to thank the reviewers again for providing us with the feedback and useful suggestions.

Sincerely,

The authors
